# Glucocorticoid use is associated with an increased risk of hypertension

**DOI:** 10.1093/rheumatology/keaa209

**Published:** 2020-06-27

**Authors:** Ruth E Costello, Belay B Yimer, Polly Roads, Meghna Jani, William G Dixon

**Affiliations:** 1 Centre for Epidemiology Versus Arthritis, Centre for Musculoskeletal Research, Manchester Academic Health Science Centre, University of Manchester, Manchester; 2 Department of Rheumatology, Salford Royal NHS Foundation Trust, Salford, UK

**Keywords:** rheumatoid arthritis, cardiovascular, epidemiology, immunosuppressants, primary care rheumatology

## Abstract

**Objectives:**

Patients with RA are frequently treated with glucocorticoids (GCs), but evidence is conflicting about whether GCs are associated with hypertension. The aim of this study was to determine whether GCs are associated with incident hypertension in patients with RA.

**Methods:**

A retrospective cohort of patients with incident RA and without hypertension was identified from UK primary care electronic medical records (Clinical Practice Research Datalink). GC prescriptions were used to determine time-varying GC use, dose and cumulative dose, with a 3 month attribution window. Hypertension was identified through either: blood pressure measurements >140/90 mmHg, or antihypertensive prescriptions and a Read code for hypertension. Unadjusted and adjusted Cox proportional hazards regression models were fitted to determine whether there was an association between GC use and incident hypertension.

**Results:**

There were 17 760 patients in the cohort. A total of 7421 (42%) were prescribed GCs during follow-up. The incident rate of hypertension was 64.1 per 1000 person years (95% CI: 62.5, 65.7). The Cox proportional hazards model indicated that recent GC use was associated with a 17% increased hazard of hypertension (hazard ratio 1.17; 95% CI: 1.10, 1.24). When categorized by dose, only doses above 7.5 mg were significantly associated with hypertension. Cumulative dose did not indicate a clear pattern.

**Conclusion:**

Recent GC use was associated with incident hypertension in patients with RA, in particular doses ≥7.5 mg were associated with hypertension. Clinicians need to consider cardiovascular risk when prescribing GCs, and ensure blood pressure is regularly monitored and treated where necessary.

**Rheumatology key messages**

- Glucocorticoid use increases the risk of hypertension in patients with RA.
- Glucocorticoid doses of ≥7.5 mg in particular are associated with hypertension.
- Blood pressure should be monitored in patients with RA prescribed glucocorticoids.

## Introduction

RA is a chronic inflammatory condition, affecting around 1% of the general population [1]. Patients with RA are at an increased risk of all-cause mortality compared with the general population [2]. Cardiovascular (CV) disease is a major driver of this: a meta-analysis showed that patients with RA have a 50% increased risk of CV mortality compared with the general population [3]. This increased risk of CV disease [4] is due not only to traditional risk factors such as smoking and hypertension, but also to disease-related factors such as disease activity, which increases inflammation [5, 6], and potentially to medication used to manage RA, for example NSAIDs [7] or glucocorticoids (GCs).

GCs are frequently prescribed in RA, with up to two-thirds of patients with RA ever prescribed GCs [8, 9]. This reflects their powerful anti-inflammatory effects, yet their use is associated with a wide range of adverse effects, such as fractures, infections, insomnia and weight gain [10]. Another less well studied but widely cited side effect of GCs is hypertension. Hypertension has been captured as one of many adverse events in clinical trials [11–14]. In placebo controlled trials of patients with a variety of rheumatic conditions (RA, polymyalgia rheumatica, GCA) there were 3–28 hypertension events per 100 patient years in those using chronic medium dose GCs (7.5 to <30 mg/day). However, the range of reported hypertension events is wide compared with other GC adverse events [15]. There have been very few studies focussed specifically on GC-induced hypertension in RA. Observational studies specifically investigating hypertension and GC use have had conflicting results: some studies have described medium to high dose GCs being associated with hypertension [16, 17], while other studies found no association [18, 19]. As hypertension may further increase CV risk, it is important to evaluate whether GCs increase the risk of hypertension and if so, how this might relate to dose. Therefore, the aim of this study was to determine whether GCs are associated with increased risk of incident hypertension in a cohort of patients with incident RA.

## Methods

### Design

This was a retrospective cohort study using data from the Clinical Practice Research Datalink (CPRD), a database of UK primary care electronic medical records. The data covers around 7% of the UK population and it has been shown to be broadly representative of the general population [20]. This study used only data from practices that were considered up to research standard (a CPRD measure indicating when practice data is up to research quality based on mortality rates and continuity of data). The study period was from 1 January 1992 until 31 June 2019. The protocol for this study has been approved by the Independent Scientific Advisory Committee (Protocol number: 11_113RA6).

### Study population

All patients with incident RA diagnosed during the study period were identified using a validated algorithm [21]. Patients were excluded if they had a diagnosis of hypertension (criteria for diagnosis described in the outcome section below) before the RA diagnosis date or were aged <18 years at RA diagnosis. Patients were followed up from RA diagnosis until leaving the practice, death or the end of the study period.

### Exposure

Oral GC prescriptions were identified through product codes. The data were prepared using a published algorithm [22] and the assumptions made are described in Supplemental Data S1 available at *Rheumatology* online. People were considered GC users for the duration of each prescription. GC dose for each prescription was converted to prednisolone equivalent doses [23]. Dose was then categorized as non-use, >0–4.9, 5–7.4, 7.5–14.9 and ≥15 mg/day. Cumulative dose was calculated by multiplying daily GC dose by the number of days prescribed, and then summing this value for all prescriptions up to that time point. Values were divided by 1000 to give cumulative dose in grams (g) rather than milligrams (mg). Categories of cumulative dose were then defined as non-use, >0 to <2.5, 2.5 to <5, 5 to <10 and ≥10 g.

### Outcome

A validated definition of hypertension was used [24] where a person was considered to have hypertension from the earliest of either: (i) two consecutive systolic blood pressure (SBP) readings ≥140 mmHg within a year, (ii) two consecutive diastolic blood pressure (DBP) readings ≥90 mmHg within a year, (iii) a hypertension Read code (see [25] and Supplemental Data S1, available at *Rheumatology* online), and on therapy with antihypertensive medications (angiotensin-converting enzyme inhibitors, alpha blockers, angiotensin receptor blockers, beta blockers, calcium channel blockers and diuretics) prescribed on at least two different dates within 6 months either side of the Read code. For criteria (i) and (ii), a person was considered hypertensive from the second BP reading as a person would not be considered hypertensive based on one BP reading. For criteria (iii), a person was considered hypertensive from the earliest of Read code or antihypertensive prescription start date. Follow-up was censored at the point of hypertension diagnosis.

### Confounders

The following covariates were included in the analyses: baseline age; gender; baseline BMI calculated using height and nearest weight measurement (if present within 5 years prior to baseline); baseline smoking status, classified as ever or never using Read codes and smoking cessation prescription codes; time-varying conventional synthetic DMARD use and time-varying prescribed NSAID use, identified using product codes where patients were considered exposed for the duration of their prescription; and Charlson comorbidity index at baseline, determined using a validated algorithm [26], where patients were considered to have the comorbidity if they had a Read code at any point from registration with the practice or up to research standard date, whichever was latest, until baseline. All these covariates were considered *a priori* confounders and were included in the analysis. All code lists can be found in Supplemental Data S1, available at *Rheumatology* online.

### Missing data

Baseline BMI and smoking status had 43% and 17% missing data, respectively. Data were imputed using multiple imputation with 47 imputations, this number was based on the fraction of missing information.

### Risk attribution model

A risk attribution model was used whereby a person was considered at risk of hypertension for 3 months after the estimated GC, DMARD and NSAID prescription end dates. This allowed for uncertainty around the start and stop dates, infrequent BP assessment and for potential long lasting effects of these drugs. All GC exposure models used this risk attribution model, therefore GC use and GC dose will be described as recent GC use and recent GC dose. In sensitivity analyses the attribution model was explored by running the same analyses with a GC exposure risk attribution model of 1 month and then 6 months, to see if this affected the results.

### Analysis

The baseline characteristics of the cohort were described stratified by whether GC was ever prescribed during follow-up. Incidence rates overall and by GC status were calculated. Cox proportional hazards regression models (unadjusted, age and gender adjusted, and adjusted for all confounders) were used to examine whether recent GC use, categories of GC dose and categories of cumulative GC dose were associated with incident hypertension.

### Accounting for possible surveillance bias

As hypertension is a potential side effect of GCs, it is plausible that people prescribed GCs may have their BP measured more often than people not prescribed GCs and therefore may have more opportunity for hypertension to be identified (a surveillance bias). To investigate this, the frequency of BP measurements was compared in the first 2 years since diagnosis stratified by the level of GC exposure. As follow-up length varied, follow-up was censored at 2 years or at hypertension diagnosis if this was prior to 2 years to allow comparison between groups. As GC use had been measured in a time-varying manner a summary variable was created to describe level of GC use over the 2 years. GC exposure was classified as ‘no GC use’, ‘intermittent GC use’, if they had <80% of follow-up with GC use in the first 2 years since diagnosis or ‘continuous GC use’ if they had ≥80% GC use in the first 2 years.

### Sensitivity analyses

CPRD data can be linked to secondary care data and area-based datasets where practices consent to linkage, with 58% of all practices currently consenting to linkage [20]. For those practices, data were linked to Hospital Episodes Statistics outpatient data and practice level deprivation data. This allowed additional adjustment for healthcare utilization and socioeconomic status in a subpopulation. Healthcare utilization was measured as a proxy for disease severity where a person was considered to have high disease activity if they had more than three rheumatology outpatient visits per year. Socioeconomic status was measured using quintiles of English Index of Multiple Deprivation (IMD) 2015. Further sensitivity analyses using a stricter definition of hypertension were conducted, where only those with a Read code for hypertension and at least two antihypertensive medication prescriptions within 6 months either side of the Read code were considered hypertensive.

### Patient and public involvement

Patients were not involved in the design, conduct or reporting of this study.

## Results

### Cohort characteristics

Of 31 657 patients with a diagnosis of RA, 13 897 (44%) had hypertension prior to RA diagnosis, resulting in 17 760 patients who were included in this cohort (supplementary Fig. S1, available at *Rheumatology* online). Those included in the cohort had a mean age 56.3 years (s.d. 12.7) and were predominantly female (68%, *N* = 12 101). Of those, 41.8% (*N* = 7421) were prescribed GCs during follow-up, and these patients were slightly older (mean age 57.7 *vs* 55.3 years of those never prescribed GCs), were predominantly female, had a history of smoking and had more comorbidities compared with those not prescribed GCs during follow-up (Table 1).

**Table 1:** Baseline characteristics of cohort overall and stratified by glucocorticoid use during follow-up

	Overall	Never prescribed GCs during follow-up	Ever prescribed GCs during follow-up
*N*	17 760	10 339 (%)	7421 (%)
Baseline age [mean (s.d.)]	56.31 (12.7)	55.31 (12.4)	57.72 (13.1)
Female gender (%)	12 101 (68.1)	7139 (69.0)	4962 (66.9)
Baseline ever smoker (%)^a^	8817 (60.0)	4936 (57.5)	3881 (63.4)
Baseline BMI [mean (s.d.)]^a^	26.89 (5.45)	26.95 (5.44)	26.79 (5.47)
Baseline BMI category (%)			
Underweight	219 (2.2)	104 (1.8)	115 (2.7)
Normal	3864 (38.8)	2238 (38.7)	1626 (38.8)
Overweight	3541 (35.5)	2072 (35.8)	1469 (35.1)
Obese	2084 (20.9)	1217 (21.0)	867 (20.7)
Morbidly obese	261 (2.6)	152 (2.6)	109 (2.6)
Baseline Charlson comorbidity index (%)			
0	13 760 (77.5)	8435 (81.6)	5325 (71.8)
1	2845 (16.0)	1333 (12.9)	1512 (20.4)
2	786 (4.4)	388 (3.8)	398 (5.4)
3 or more	369 (2.1)	183 (1.8)	186 (2.5)
IMD quintile (%)^a^			
1	1415 (15.4)	755 (15.1)	660 (15.7)
2	1765 (19.2)	960 (19.2)	805 (19.1)
3	1872 (20.3)	1009 (20.2)	863 (20.5)
4	1920 (20.9)	1059 (21.2)	861 (20.4)
5	2233 (24.3)	1206 (24.2)	1027 (24.4)
GC use prior to RA diagnosis (%)	3383 (19.0)	628 (6.1)	2755 (37.1)
Cumulative GC dose in year prior to baseline [mean (s.d.)]	334.75 (1242.3)	55.80 (366.6)	723.37 (1802.0)

a There were missing data for the following variables: ever smoking: *N* = 3057 (17.2%); baseline BMI: *N* = 7791 (43.9%); IMD 2010: *N* = 8555 (48.2%). GC: glucocorticoid; IMD: English Index of Multiple Deprivation.

**Fig. 1:**
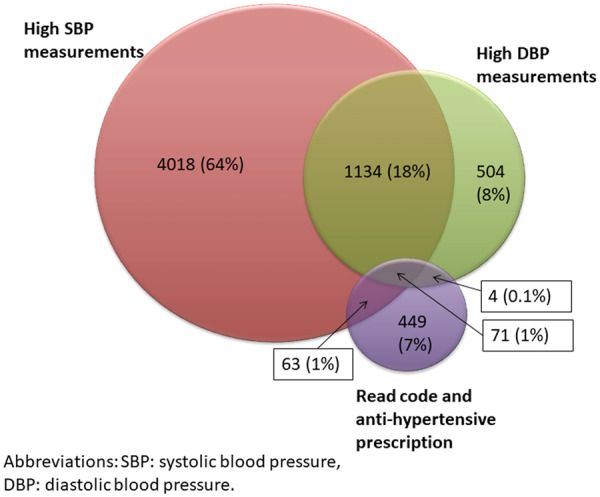
Venn diagram showing how hypertension was identified

There were 6243 cases of incident hypertension over 97 547 person years (pyrs) of follow-up, giving an incident rate of 64.1 per 1000 pyrs (95% CI: 62.5, 65.7). Cases were most frequently first identified through consecutive high SBP measurements alone (*N* = 4018, 64%), followed by consecutive high SBP and DBP measurements (*N* = 1134, 18%) and consecutive high DBP measurements alone (*n* = 504, 8%). Only 7% (*N* = 449) were identified first through antihypertensive prescriptions and Read codes alone (Fig. 1). Of those identified through high BP measurements, 60% (*N* = 3396/5656) were subsequently prescribed antihypertensive medication.

### Glucocorticoid association with hypertension

In those exposed to GCs there were 1321 cases of incident hypertension with an incidence rate of 87.6 per 1000 pyrs. In those unexposed there were 4922 cases with an incidence rate of 59.7 per 1000 pyrs. (Table 2).

**Table 2:** Number of cases and rate of hypertension by GC status

	Exposed to GCs	Unexposed to GCs	Overall
Total number^a^	7421	16 850	17 760
Follow-up time (days)	15 076	82 382	97 457
Cases of hypertension	1321	4922	6243
Incident rate, per 1000 person-years (95% CI)	87.6 (83.0, 92.4)	59.7 (58.1, 61.4)	64.1 (62.5, 65.7)

a As GC use is time-varying people could be in both categories, therefore total number across both categories is greater than the total number of people in the study. GC: glucocorticoid.

The unadjusted Cox proportional hazards model for recent GC use showed GC use was associated with a 44% increased hazard of hypertension [hazard ratio (HR) 1.44; 95% CI: 1.35, 1.53]; when fully adjusted this was attenuated to 17% increased hazard but remained statistically significant (HR 1.17; 95% CI: 1.10, 1.24). The unadjusted model for categories of recent exposure dosage showed all GC dosage categories were associated with hypertension. When fully adjusted, only doses of ≥7.5 mg were statistically significant, indicating increased hazard of hypertension (7.5–14.9 mg: HR 1.18; 95% CI: 1.08, 1.29; ≥15 mg: HR 1.36; 95% CI: 1.18, 1.56). Doses <7.5 mg had increased hazard but were not statistically significant. The unadjusted model for categories of cumulative dose showed all categories were significantly associated with hypertension, but when fully adjusted there was no clear pattern. Only the category of 5–9.99 g was statistically significant, though ≥10 g had a similar point estimate (Table 3). Point estimates for the covariates in the adjusted models were in the expected direction, with leflunomide having the biggest effect and NSAIDs having a similar magnitude of effect on hypertension as recent GC use (supplementary Table S1, available at *Rheumatology* online).

**Table 3:** Unadjusted and adjusted Cox proportional hazards regression model

	Unadjusted [HR (95% CI)]	Age and gender adjusted [HR (95% CI)]	Fully adjusted^a^ [HR (95% CI)]
Recent GC use	1.44 (1.35, 1.53)	1.23 (1.16, 1.31)	1.17 (1.10, 1.24)
Recent GC dose			
No GC use	Reference	Reference	Reference
>0–4.9 mg	1.35 (1.21, 1.53)	1.13 (1.01, 1.28)	1.10 (0.98, 1.24)
5–7.4 mg	1.40 (1.22, 1.60)	1.11 (0.97, 1.27)	1.07 (0.93, 1.23)
7.5–14.9 mg	1.44 (1.33, 1.57)	1.26 (1.16, 1.38)	1.18 (1.08, 1.29)
≥15 mg	1.60 (1.40, 1.84)	1.45 (1.27, 1.66)	1.36 (1.18, 1.56)
Cumulative dose			
No GC use	Reference	Reference	Reference
>0–2.49 g	1.14 (1.05, 1.23)	1.04 (0.96, 1.12)	1.00 (0.92, 1.08)
2.5–4.99 g	1.16 (1.06, 1.27)	1.04 (0.95, 1.13)	0.99 (0.90, 1.08)
5–9.99 g	1.36 (1.24, 1.48)	1.18 (1.08, 1.30)	1.12 (1.02, 1.22)
≥10 g	1.35 (1.24, 1.49)	1.16 (1.06, 1.27)	1.07 (0.97, 1.17)

a Adjusted for baseline age, gender, baseline BMI, baseline ever smoking, Charlson comorbidity index, time-varying synthetic DMARD use and time-varying NSAID use. HR: hazard ratio; GC: glucocorticoid.

### Possible surveillance bias

**Fig. 2:**
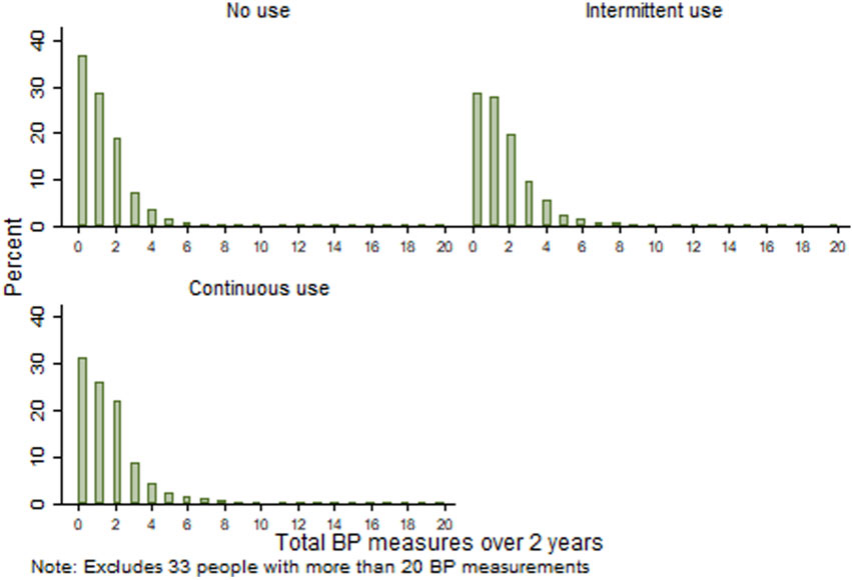
Number of blood pressure measurements over 2 years, by glucocorticoid use category

When the cohort follow-up was censored to 2 years, most patients (73%) had at least 2 years’ follow-up. The majority of the cohort did not use GCs during this period (*n* = 12 124, 68.3%), 3461 (19.5%) had intermittent use and 2175 (12.3%) had continuous use. There were no differences in the frequency of BP measurements between the groups (Table 4 and Fig. 2), suggesting that surveillance bias was not present.

**Table 4:** Frequency of blood pressure measurements by categories of GC use over 2 years

GC use category	*N* (%)	At least 1 BP measurement [*n* (%)]	Median number of measurements (IQR)	More than 2 BP measurements [*n* (%)]	Maximum number of measurements
No use	12 124 (68.3)	7714 (65.6)	1 (0–2)	1995 (16.5)	34
Intermittent use	3461 (19.5)	2477 (71.6)	1 (0–2)	841 (24.3)	39
Continuous use	2175 (12.3)	1492 (68.6)	1 (0–2)	448 (20.6)	25

GC: glucocorticoid; BP: blood pressure; IQR: interquartile range.

### Sensitivity analyses

There were 5860 patients with linkage to Hospital Episodes Statistics outpatient data, of whom 1487 developed incident hypertension giving an incident rate of 59.9 per 1000 pyrs (95% CI: 57.0, 63.0). Additional adjustment for our proxy for disease activity and IMD 2015 did not substantively change the results: the recent GC use HR was slightly lower (HR 1.14; 95% CI: 1.00, 1.29) and only doses ≥15 mg were statistically significant. Though the dose category 7.5–14.9 mg just missed significance, this was the same regardless of the additional adjustment for disease activity and IMD 2015 (supplementary Table S2, available at *Rheumatology* online). When the attribution window was increased to 6 months the results were broadly similar (supplementary Table S3, available at *Rheumatology* online). When the attribution window was reduced to 1 month the results were broadly similar, though the lowest category of GC dose (>0–4.9 mg) was just statistically significant (HR:1.16; 95% CI: 1.02, 1.31) (supplementary Table S4, available at *Rheumatology* online). There were 2002 cases of hypertension using the strict hypertension definition (two or more antihypertensive prescriptions within 6 months either side of a Read code). Although there were only 449 patients initially identified through this strict definition, many of those who were first identified through BP measurements alone later went on to meet the criteria using the strict definition. The results using this strict definition of hypertension were similar, the HR was slightly lower for recent GC use (HR 1.13; 95% CI: 1.01, 1.27). Doses >7.5 mg were not statistically significant, although they remained in the direction of increased risk (supplementary Table S5, available at *Rheumatology* online).

## Discussion

This study found that GC use was associated with a 17% overall increased risk of hypertension in patients with incident RA and without hypertension at RA diagnosis. When GC use was stratified by dose categories, doses <7.5 mg were not found to be associated with hypertension, indicating that low doses were less of a concern, although the point estimates were in the direction of increased risk for all categories of GC dose. There was no clear pattern seen for cumulative dose, but this may be due to the nature of the measure itself, as a small cumulative dose may represent a person prescribed a low dose for a long period or a person prescribed a high dose for a short period, making it difficult to draw conclusions in terms of the entire exposed period. Additionally, 40% of patients prescribed GCs with hypertension (defined by consecutive high SBP or DBP readings) were not prescribed an antihypertensive at any point during the study duration. Whilst some may have been offered lifestyle advice, left untreated this has important implications in terms of addressing modifiable risk factors in an RA population already at increased risk of CV disease.

Differences in the frequency of BP measurement by GC exposure were not seen, providing reassurance that surveillance bias does not explain the findings. Importantly, around 30% of the cohort did not have their BP measured during the first 2 years after diagnosis. EULAR recommends monitoring and treatment of CV risk factors in RA [27] and hypertension in GC-treated patients [15]. This study highlights that this may not be the case overall in RA with regards to monitoring and treating high BP in primary care. Given this finding, it is important for primary care physicians (and rheumatologists) to be aware that GCs increase the risk of hypertension, and to monitor patients’ BP more vigilantly while GCs are prescribed.

### Previous studies

These results concur with a single-centre cross-sectional study, where long-term (<6 months use) medium dose (≤7.5 mg) prednisolone was associated with hypertension [16], and a study of patients in a German registry where patients who were prescribed GC doses >7.5 mg for >6 months had higher proportions of self-reported ‘increase in blood pressure’ [17]. However, our results do not concur with another study that used CPRD data to investigate adverse effects associated with GC use, including hypertension. They did not find an association between GC use and hypertension; however, only a Read code was used to identify hypertension, so cases may have been missed and may explain why their results were different from this study [18].

### Incidence of GC-associated hypertension

This study provides an estimate of incidence of hypertension associated with GC use, which allows more informed decisions for the patient. A UK study using primary care electronic records has estimated the incidence of hypertension in patients with RA [28]. This study found a lower incident rate of hypertension, 336.2 per 10 000 pyrs, and a higher proportion being treated (85%) compared with our study (60%). However, this study only identified hypertension using Read codes and/or antihypertensive prescriptions, which means patients with high BP but not coded or treated are missed, which may explain the differences found compared with our study.

### Strengths and limitations

This was a large retrospective cohort study using routinely collected data with a number of strengths. The use of prescription data allowed more precise measurement of time-varying GC use, and a variety of attribution models were used to test the impact of our assumptions when preparing the data. Hypertension diagnosis has not been consistently defined across the few studies using CPRD data, and in our study hypertension was identified through BP measurements or a Read code and antihypertensive prescriptions. This definition has been validated in Spanish primary care electronic health records [24] and allowed a more robust identification of the outcome. As anti-hypertensive medication can be prescribed for other indications, it was important to use both Read code for hypertension and antihypertensive medication prescriptions to ensure antihypertensive medication was not prescribed for another indication.

Alongside these strengths there are some limitations. Misclassification of medication use is a possibility; as CPRD data only contains prescriptions, we do not know if these medications were dispensed. However, we used a number of attribution models to allow for potential differences in when prescriptions would be dispensed. This study was designed specifically to examine incident hypertension and thus included only patients without prior hypertension. Further work is needed to understand how GCs may affect BP in those already diagnosed with hypertension. Although we need to be careful of over-interpretation of covariate point estimates [29], the variables adjusted for were in the expected direction. However, there are some variables that cannot be measured in CPRD: disease severity is not available. However, currently there is no evidence that high disease activity is associated with high BP, suggesting that confounding by indication is less of a concern [30, 31]. There is not a validated proxy for disease severity in CPRD; however, we have conducted a sensitivity analysis using a pragmatic proxy for disease severity and this did not alter the results. As biologics are prescribed in secondary care this is not well captured in CPRD. TNF inhibitors have been shown to reduce BP [11]; however, it has been shown that those prescribed biologics are more likely to have received GCs [32]. As we would expect GCs to increase BP, if TNF inhibitors are prescribed more frequently in those prescribed GCs we would expect the effect of GCs on BP to be underestimated. Therefore any unmeasured confounding would not explain our positive findings.

### Conclusions

This study found that GC use was associated with incident hypertension in patients with RA, and in particular doses >7.5 mg were associated with hypertension. There was an incidence rate of 64.1 per 1000 pyrs. BP was not frequently monitored in primary care and a large proportion of RA patients on GCs with high BP readings were untreated. Given that patients with RA are already at increased risk of CV disease, it is important that these patients should have their BP checked regularly and treated appropriately.

*Funding*: This work was supported by the Centre for Epidemiology Versus Arthritis (Grant number 21755) and supported by the National Institute for Health Research Manchester Biomedical Research Centre.

*Disclosure statement*: W.G.D. has received consultancy fees from Google and Beyer unrelated to this work. The other authors have declared no conflicts of interest.

## Supplementary data

Supplementary data are available at *Rheumatology* online.

## Supplementary Material

keaa209_Supplementary_Data
