# Supplementary material for: Glucocorticoid use is associated with an increased risk of hypertension
Source: Rheumatology (Oxford). 2020 Jun 27;60(1):132–9. doi: 10.1093/rheumatology/keaa209 (PMC7785301; doi:10.1093/rheumatology/keaa209)
Supplement: keaa209_Supplementary_Data [file keaa209_supplementary_data.zip › keaa209-suppl_data/rhe-19-2175-File004.docx]

## SUPPLEMENTARY MATERIAL

## S1 Codelists for disease and drug definitions

Rheumatoid arthritis - medcodes

| **Medcode** | **Description** |
| --- | --- |
| 844 | Rheumatoid arthritis |
| 5723 | Rheumatoid nodule |
| 6639 | H/O: rheumatoid arthritis |
| 6916 | Seronegative rheumatoid arthritis |
| 8350 | Flare of rheumatoid arthritis |
| 9707 | Seropositive errosive rheumatoid arthritis |
| 9954 | Rheumatoid lung |
| 12019 | Seropositive rheumatoid arthritis, unspecified |
| 17412 | Rheumatoid arthrit. monitoring |
| 21358 | Rheumatoid arthritis of shoulder |
| 23552 | Felty's syndrome |
| 23834 | Adult Still's Disease |
| 27603 | Rheumatoid arthritis and other inflammatory polyarthropathy |
| 28853 | Fibrosing alveolitis associated with rheumatoid arthritis |
| 30548 | Rheumatoid vasculitis |
| 31054 | Rheumatoid arthritis - multiple joint |
| 31209 | Myopathy due to rheumatoid arthritis |
| 31724 | Rheumatoid lung |
| 32001 | Adult-onset Still's disease |
| 37431 | Rheumatoid arthropathy + visceral/systemic involvement NOS |
| 41941 | Rheumatoid arthritis of PIP joint of finger |
| 42299 | Rheumatoid arthritis of MCP joint |
| 43816 | Rheumatoid carditis |
| 44203 | Other rheumatoid arthritis of spine |
| 44743 | Rheumatoid arthritis of cervical spine |
| 46436 | Rheumatoid lung disease |
| 48832 | Rheumatoid arthritis of wrist |
| 49067 | Rheumatoid arthritis of hip |
| 49227 | Other rheumatoid arthropathy + visceral/systemic involvement |
| 49787 | Rheumatoid myocarditis |
| 50863 | Rheumatoid arthritis of knee |
| 51238 | Rheumatoid arthritis of 1st MTP joint |
| 51239 | Rheumatoid arthritis of ankle |
| 53621 | Rheumatoid nodule |
| 56202 | [X]Seropositive rheumatoid arthritis, unspecified |
| 56838 | Caplan's syndrome |
| 59738 | Rheumatoid arthritis of elbow |
| 62401 | Polyneuropathy in rheumatoid arthritis |
| 63198 | Rheumatoid arthritis of DIP joint of finger |
| 63365 | Rheumatoid arthritis of distal radio-ulnar joint |
| 70221 | [X]Other specified rheumatoid arthritis |
| 70658 | Rheumatoid arthritis of talonavicular joint |
| 71784 | Rheumatoid arthritis of other tarsal joint |
| 73619 | Rheumatoid arthritis of subtalar joint |
| 93715 | [X]Other seropositive rheumatoid arthritis |
| 99414 | Rheumatoid arthritis of lesser MTP joint |
| 100187 | Disease activity score in rheumatoid arthritis |
| 100776 | Rheumatoid arthritis of sacro-iliac joint |
| 100914 | Rheumatoid arthritis of acromioclavicular joint |
| 102088 | Delivery of rehabilitation for rheumatoid arthritis |

Glucocorticoids – product codes

| **prodcode** | **Product name** |
| --- | --- |
| 44 | Prednisolone 5mg gastro-resistant tablets |
| 95 | Prednisolone 5mg tablets |
| 186 | Dexamethasone 500micrograms/5ml oral solution |
| 229 | Cortisone 25mg tablets |
| 557 | Prednisolone 2.5mg gastro-resistant tablets |
| 578 | Prednisolone 1mg tablets |
| 955 | Prednisolone 5mg soluble tablets |
| 1063 | Prednesol 5mg Tablet (Sovereign Medical Ltd) |
| 1280 | Dexamethasone 2mg tablets |
| 1380 | Entocort CR 3mg capsules (AstraZeneca UK Ltd) |
| 1971 | Betnesol 500microgram soluble tablets (Focus Pharmaceuticals Ltd) |
| 2130 | Methylprednisolone 4mg tablets |
| 2368 | Prednisolone 2.5mg tablet |
| 2704 | Prednisolone 25mg tablets |
| 2949 | Prednisone 5mg tablets |
| 3345 | Sintisone Tablet (Pharmacia Ltd) |
| 3418 | Hydrocortisone 10mg tablets |
| 3557 | Prednisone 1mg tablets |
| 3898 | Budesonide 3mg gastro-resistant modified-release capsules |
| 3992 | Deflazacort 6mg tablets |
| 4535 | Hydrocortisone 20mg tablets |
| 4779 | Dexamethasone 500microgram tablets |
| 4943 | Dexamethasone 2mg/5ml oral solution sugar free |
| 5157 | Dexamethasone 2mg/5ml oral solution |
| 5490 | Deltacortril 5mg gastro-resistant tablets (Alliance Pharmaceuticals Ltd) |
| 5913 | Deltacortril 2.5mg gastro-resistant tablets (Alliance Pharmaceuticals Ltd) |
| 6095 | Budesonide 3mg gastro-resistant capsules |
| 6098 | Hydrocortone 10mg tablets (Auden McKenzie (Pharma Division) Ltd) |
| 6339 | Hydrocortisone 2.5mg muco-adhesive buccal tablets sugar free |
| 7286 | Betamethasone 500microgram soluble tablets sugar free |
| 7548 | Cortisone 5mg capsules |
| 8261 | Medrone 16mg tablets (Pfizer Ltd) |
| 9375 | Deflazacort 1mg tablets |
| 9727 | Prednisolone 50mg tablets |
| 9994 | Decadron 500microgram tablets (Merck Sharp & Dohme Ltd) |
| 10552 | Methylprednisolone 16mg tablets |
| 10574 | Cortisone acetate 5mg tablets |
| 10683 | Medrone 2mg tablets (Pfizer Ltd) |
| 10684 | Methylprednisolone 2mg tablets |
| 10754 | Hydrocortistab 20mg Tablet (Waymade Healthcare Plc) |
| 10864 | Betamethasone 500microgram tablets |
| 11149 | Betnelan 500microgram tablets (Focus Pharmaceuticals Ltd) |
| 12398 | Cortelan 25mg Tablet (Glaxo Laboratories Ltd) |
| 12400 | Cortisyl 25mg Tablet (Aventis Pharma) |
| 13043 | Hydrocortone 20mg tablets (Auden McKenzie (Pharma Division) Ltd) |
| 14076 | Hydrocortisone 5mg/5ml Oral solution |
| 14172 | Methylprednisolone 100mg tablets |
| 15555 | Medrone 4mg tablets (Pfizer Ltd) |
| 15617 | Ledercort 4mg Tablet (Wyeth Pharmaceuticals) |
| 16525 | Budenofalk 3mg gastro-resistant capsules (Dr. Falk Pharma UK Ltd) |
| 17410 | Deflazacort 30mg tablets |
| 18042 | Medrone 100mg tablets (Pfizer Ltd) |
| 18637 | Cortistab 25mg Tablet (Waymade Healthcare Plc) |
| 19141 | Prednisolone 5mg soluble tablets (AMCo) |
| 19908 | Triamcinolone 2mg Tablet |
| 20095 | Precortisyl forte 25mg Tablet (Aventis Pharma) |
| 20577 | Calcort 6mg Tablet (Shire Pharmaceuticals Ltd) |
| 21218 | Dexsol 2mg/5ml oral solution (Rosemont Pharmaceuticals Ltd) |
| 21417 | Prednisolone 5mg tablets (A A H Pharmaceuticals Ltd) |
| 21833 | Decortisyl 5mg Tablet (Roussel Laboratories Ltd) |
| 21903 | Oradexon-organon 2mg Tablet (Organon Laboratories Ltd) |
| 22555 | Calcort 1mg tablets (Shire Pharmaceuticals Ltd) |
| 23111 | Triamcinolone 4mg Tablet |
| 23210 | Cortistab 5mg Tablet (Waymade Healthcare Plc) |
| 23512 | Precortisyl 5mg Tablet (Hoechst Marion Roussel) |
| 24014 | Ledercort 2mg Tablet (Wyeth Pharmaceuticals) |
| 25272 | Precortisyl 1mg Tablet (Hoechst Marion Roussel) |
| 27962 | Deltastab 1mg Tablet (Waymade Healthcare Plc) |
| 28375 | Prednisolone 2.5mg gastro-resistant tablets (A A H Pharmaceuticals Ltd) |
| 28376 | Prednisolone 2.5mg Gastro-resistant tablet (Biorex Laboratories Ltd) |
| 28859 | Deltastab 5mg Tablet (Waymade Healthcare Plc) |
| 29112 | Calcort 30mg tablets (Shire Pharmaceuticals Ltd) |
| 29333 | Prednisolone 5mg tablets (Actavis UK Ltd) |
| 31327 | Prednisolone steaglate 6.65mg tablet |
| 31532 | Prednisolone 5mg gastro-resistant tablets (A A H Pharmaceuticals Ltd) |
| 32803 | Prednisolone 5mg gastro-resistant tablets (Actavis UK Ltd) |
| 32835 | Prednisolone 5mg tablets (Wockhardt UK Ltd) |
| 33691 | Prednisolone 5mg Gastro-resistant tablet (Biorex Laboratories Ltd) |
| 33988 | Prednisolone 5mg Tablet (Co-Pharma Ltd) |
| 33990 | Prednisolone 5mg Tablet (IVAX Pharmaceuticals UK Ltd) |
| 34109 | Prednisolone 5 mg gastro-resistant tablet |
| 34393 | Prednisolone 5mg gastro-resistant tablets (Teva UK Ltd) |
| 34404 | Prednisolone 1mg tablets (Actavis UK Ltd) |
| 34452 | Prednisolone 1mg tablets (A A H Pharmaceuticals Ltd) |
| 34461 | Prednisolone 2.5mg gastro-resistant tablets (Actavis UK Ltd) |
| 34631 | Prednisolone 1mg Tablet (Co-Pharma Ltd) |
| 34660 | Prednisolone 1mg tablets (Kent Pharmaceuticals Ltd) |
| 34748 | Prednisolone 1mg tablets (Teva UK Ltd) |
| 34781 | Prednisolone 5mg tablets (Kent Pharmaceuticals Ltd) |
| 34801 | Dexamethasone 0.5mg/5ml Oral solution (Rosemont Pharmaceuticals Ltd) |
| 34880 | Dexamethasone 2mg tablets (Aspen Pharma Trading Ltd) |
| 34914 | Prednisolone 1mg Tablet (Celltech Pharma Europe Ltd) |
| 34915 | Dexamethasone 500microgram tablets (Organon Laboratories Ltd) |
| 34978 | Prednisolone 1mg tablets (Wockhardt UK Ltd) |
| 36055 | Dexamethasone 2mg Tablet (Hillcross Pharmaceuticals Ltd) |
| 37203 | Beclometasone 5mg gastro-resistant modified-release tablets |
| 38022 | Hydrocortisone 10mg/5ml oral suspension |
| 38054 | Hydrocortisone Tablet |
| 38407 | Prednisolone 20mg tablet |
| 39067 | Clipper 5mg gastro-resistant modified-release tablets (Chiesi Ltd) |
| 41335 | Calcort 6mg tablets (Sanofi) |
| 41515 | Prednisolone 5mg tablets (Teva UK Ltd) |
| 41745 | Prednisolone 25mg tablets (Zentiva) |
| 43544 | Prednisone 5mg Tablet (Knoll Ltd) |
| 44380 | Prednisone 1mg modified-release tablets |
| 44723 | Prednisone 5mg modified-release tablets |
| 44802 | Lodotra 5mg modified-release tablets (Napp Pharmaceuticals Ltd) |
| 44803 | Lodotra 2mg modified-release tablets (Napp Pharmaceuticals Ltd) |
| 45234 | Dexamethasone 100microgram capsules |
| 45302 | Prednisolone 5mg Tablet (Biorex Laboratories Ltd) |
| 46280 | Hydrocortisone 2.5mg muco-adhesive buccal tablets sugar free (Auden McKenzie (Pharma Division) Ltd) |
| 46711 | Prednisone 2mg modified-release tablets |
| 47142 | Prednisolone 5mg Soluble tablet (Amdipharm Plc) |
| 47225 | Budesonide 9mg gastro-resistant granules sachets |
| 48088 | Budenofalk 9mg gastro-resistant granules sachets (Dr. Falk Pharma UK Ltd) |
| 50225 | Betnesol 500microgram soluble tablets (Waymade Healthcare Plc) |
| 51722 | Hydrocortisone 5mg/5ml oral suspension |
| 51753 | Prednisolone 1mg tablets (Co-Pharma Ltd) |
| 51824 | Hydrocortisone 5mg/5ml oral suspension sugar free |
| 51849 | Hydrocortisone 1mg/5ml oral suspension |
| 51871 | Hydrocortisone 2mg capsules |
| 51872 | Hydrocortisone 2.5mg capsules |
| 51997 | Budesonide 9mg gastro-resistant granules sachets |
| 52053 | Hydrocortisone 3mg/5ml oral suspension |
| 52396 | Dexamethasone 1mg/5ml oral solution |
| 53143 | Cortisone 25mg tablets (A A H Pharmaceuticals Ltd) |
| 53207 | Dexamethasone tablets |
| 53313 | Prednisolone 20mg/5ml oral suspension |
| 53336 | Prednisolone 25mg tablets (A A H Pharmaceuticals Ltd) |
| 53705 | Cortisone acetate 5mg Capsule (Martindale Pharmaceuticals Ltd) |
| 53953 | Hydrocortisone 5mg modified-release tablets |
| 54118 | Prednisolone 25mg/5ml oral suspension |
| 54432 | Lodotra 1mg modified-release tablets (Napp Pharmaceuticals Ltd) |
| 54434 | Prednisolone 2.5mg/5ml oral suspension |
| 54793 | Dexamethasone 2mg/5ml oral suspension |
| 54794 | Hydrocortisone 20mg modified-release tablets |
| 55024 | Prednisolone 5mg/5ml oral solution |
| 55401 | Dexamethasone 500microgram tablets (A A H Pharmaceuticals Ltd) |
| 55480 | Prednisolone 2.5mg gastro-resistant tablets (Alliance Pharmaceuticals Ltd) |
| 56144 | Budenofalk 9mg gastro-resistant granules sachets (Dr. Falk Pharma UK Ltd) |
| 56319 | Hydrocortisone 2.5mg muco-adhesive buccal tablets sugar free (A A H Pharmaceuticals Ltd) |
| 56347 | Dexamethasone 5mg/5ml oral solution |
| 56443 | Dexamethasone 10mg/5ml oral solution |
| 56891 | Prednisolone 1mg tablets (Waymade Healthcare Plc) |
| 57931 | Hydrocortisone 20mg tablets (Teva UK Ltd) |
| 58000 | Prednisolone 5mg tablets (Almus Pharmaceuticals Ltd) |
| 58061 | Prednisone 50mg tablets |
| 58234 | Prednisolone 10mg/5ml oral solution |
| 58369 | Prednisolone 5mg tablets (Boston Healthcare Ltd) |
| 58384 | Prednisolone 1mg tablets (Almus Pharmaceuticals Ltd) |
| 58474 | Dexamethasone 2mg/5ml oral solution sugar free (A A H Pharmaceuticals Ltd) |
| 58592 | Plenadren 20mg modified-release tablets (ViroPharma Ltd) |
| 58987 | Prednisolone 5mg gastro-resistant tablets (Phoenix Healthcare Distribution Ltd) |
| 59229 | Dilacort 5mg gastro-resistant tablets (Auden McKenzie (Pharma Division) Ltd) |
| 59283 | Dilacort 2.5mg gastro-resistant tablets (Auden McKenzie (Pharma Division) Ltd) |
| 59338 | Prednisolone 1mg/5ml oral solution |
| 59418 | Plenadren 5mg modified-release tablets (ViroPharma Ltd) |
| 59912 | Prednisolone 5mg gastro-resistant tablets (Waymade Healthcare Plc) |
| 60064 | Dexamethasone 10mg/5ml oral solution sugar free |
| 60120 | Dexamethasone 2mg tablets (Alliance Healthcare (Distribution) Ltd) |
| 60421 | Prednisolone 5mg tablets (Co-Pharma Ltd) |
| 60946 | Entocort CR 3mg capsules (Waymade Healthcare Plc) |
| 61132 | Prednisolone 1mg tablets (Boston Healthcare Ltd) |
| 61162 | Prednisolone 5mg tablets (Waymade Healthcare Plc) |
| 61689 | Prednisolone 5mg soluble tablets (A A H Pharmaceuticals Ltd) |
| 61791 | Hydrocortisone 2.5mg muco-adhesive buccal tablets sugar free (Waymade Healthcare Plc) |
| 62656 | Prednisone 5mg Tablet (Hillcross Pharmaceuticals Ltd) |
| 62909 | Dexamethasone 2mg tablets (A A H Pharmaceuticals Ltd) |
| 63066 | Prednisolone 2.5mg tablets |
| 63082 | Prednisolone 20mg tablets |
| 63138 | Hydrocortisone 5mg/5ml oral solution |
| 63172 | Prednisolone 10mg tablets |
| 63214 | Prednisolone 5mg soluble tablets (Alliance Healthcare (Distribution) Ltd) |
| 63549 | Prednisolone 1mg/ml oral solution (Logixx Pharma Solutions Ltd) |
| 63791 | Prednisolone 5mg/5ml oral solution unit dose |
| 63893 | Budesonide 9mg modified-release tablets |
| 64007 | Pevanti 10mg tablets (AMCo) |
| 64008 | Pevanti 2.5mg tablets (AMCo) |
| 64009 | Pevanti 20mg tablets (AMCo) |
| 64050 | Martapan 2mg/5ml oral solution (Martindale Pharmaceuticals Ltd) |
| 64059 | Hydrocortisone 2.5mg/5ml oral suspension |
| 64128 | Pevanti 5mg tablets (AMCo) |
| 64221 | Prednisolone 5mg/5ml oral suspension |
| 64235 | Betamethasone 500microgram soluble tablets sugar free (Alliance Healthcare (Distribution) Ltd) |
| 64416 | Prednisolone 10mg/ml oral solution sugar free |
| 64557 | Cortiment 9mg modified-release tablets (Ferring Pharmaceuticals Ltd) |
| 64747 | Dexamethasone 2mg/5ml oral solution |
| 64766 | Dexamethasone 20mg/5ml oral solution sugar free |
| 64787 | Hydrocortisone 10mg tablets (Almus Pharmaceuticals Ltd) |

Hypertension Read codes

| Read code | Description |
| --- | --- |
| G2...00 | Hypertensive disease |
| G20..00 | Essential hypertension |
| G200.00 | Malignant essential hypertension |
| G201.00 | Benign essential hypertension |
| G20..11 | High blood pressure |
| G202.00 | Systolic hypertension |
| G203.00 | Diastolic hypertension |
| G20z.00 | Essential hypertension NOS |
| G20z.11 | Hypertension NOS |
| G24..00 | Secondary hypertension |
| G240.00 | Secondary malignant hypertension |
| G240000 | Secondary malignant renovascular hypertension |
| G240z00 | Secondary malignant hypertension NOS |
| G241.00 | Secondary benign hypertension |
| G241000 | Secondary benign renovascular hypertension |
| G241z00 | Secondary benign hypertension NOS |
| G244.00 | Hypertension secondary to endocrine disorders |
| G24z.00 | Secondary hypertension NOS |
| G24z000 | Secondary renovascular hypertension NOS |
| G24z100 | Hypertension secondary to drug |
| G24zz00 | Secondary hypertension NOS |
| G2y..00 | Other specified hypertensive disease |
| G2z..00 | Hypertensive disease NOS |
| Gyu2.00 | [X]Hypertensive diseases |
| Gyu2000 | [X]Other secondary hypertension |

Antihypertensive medication

| **prodcode** | **Product name** |
| --- | --- |
| 119 | Doxazosin 1mg tablets |
| 445 | Prazosin 1mg tablets and Prazosin 500microgram tablets |
| 493 | Doxazosin 2mg tablets |
| 582 | Doxazosin 4mg modified-release tablets |
| 591 | Prazosin 1mg tablets |
| 726 | Prazosin 2mg tablets |
| 755 | Cardura XL 4mg tablets (Pfizer Ltd) |
| 1292 | Hypovase 1mg tablets (Pfizer Ltd) |
| 1294 | Doxazosin 4mg tablets |
| 1455 | Prazosin 500microgram tablets |
| 2117 | Doralese Tiltab 20mg tablets (Chemidex Pharma Ltd) |
| 2345 | Hytrin BPH tablets starter pack (Amdipharm Plc) |
| 2346 | Hytrin 5mg Tablet (Abbott Laboratories Ltd) |
| 2347 | Hytrin 10mg Tablet (Abbott Laboratories Ltd) |
| 2348 | Hytrin bph 10mg Tablet (Amdipharm Plc) |
| 2816 | Indoramin 20mg tablets |
| 3470 | Terazosin 1mg tablets |
| 3715 | Prazosin 5mg tablets |
| 3923 | Terazosin BPH starter pack 7x1mg with 14x2mg with 7x5mg |
| 3924 | Terazosin 5mg tablets |
| 4111 | Hypovase 500microgram tablets (Pfizer Ltd) |
| 4449 | Cardura 1mg tablets (Pfizer Ltd) |
| 4637 | Terazosin 2mg tablets |
| 4694 | Terazosin 2mg tablets and Terazosin 1mg tablets |
| 4802 | Cardura 2mg tablets (Pfizer Ltd) |
| 4875 | Terazosin 10mg tablets |
| 5183 | Hypovase 2mg tablets (Pfizer Ltd) |
| 5337 | Hytrin bph 5mg Tablet (Amdipharm Plc) |
| 5496 | Doxazosin 8mg modified-release tablets |
| 5618 | Cardura XL 8mg tablets (Pfizer Ltd) |
| 5815 | Indoramin 25mg tablets |
| 7547 | Doxadura 2mg tablets (Discovery Pharmaceuticals Ltd) |
| 7549 | Doxadura 1mg tablets (Discovery Pharmaceuticals Ltd) |
| 7759 | Phenoxybenzamine 10mg capsules |
| 8076 | Hytrin tablets starter pack (Amdipharm Plc) |
| 8077 | Hytrin 2mg Tablet (Abbott Laboratories Ltd) |
| 8086 | Cardura 4mg Tablet (Pfizer Ltd) |
| 8198 | Hypovase 5mg Tablet (Pfizer Ltd) |
| 8942 | Dibenyline 10mg capsules (Mercury Pharma Group Ltd) |
| 9019 | Indoramin 50mg Tablet |
| 10088 | Doxadura 4mg tablets (Discovery Pharmaceuticals Ltd) |
| 11394 | Baratol 25mg Tablet (Shire Pharmaceuticals Ltd) |
| 12518 | Hypovase tablets B.D. starter pack (Pfizer Ltd) |
| 12545 | Phentolamine 10mg/1ml solution for injection ampoules |
| 16201 | Hytrin bph 2mg Tablet (Amdipharm Plc) |
| 19193 | Doxazosin 2mg tablets (Teva UK Ltd) |
| 19216 | Doxazosin 4mg tablets (IVAX Pharmaceuticals UK Ltd) |
| 20369 | Doxazosin 1mg/5ml oral suspension |
| 23010 | Rogitine 10mg/1ml solution for injection ampoules (Alliance Pharmaceuticals Ltd) |
| 25487 | Cascor 2mg tablets (Ranbaxy (UK) Ltd) |
| 25551 | Cascor 4mg tablets (Ranbaxy (UK) Ltd) |
| 34553 | Doxazosin 4mg tablets (Generics (UK) Ltd) |
| 34715 | Doxazosin 1mg tablets (A A H Pharmaceuticals Ltd) |
| 35272 | Doxadura XL 4mg tablets (Discovery Pharmaceuticals Ltd) |
| 35603 | Doxazosin 4mg/5ml oral suspension |
| 36023 | Cardozin xl 4mg Tablet (Hillcross Pharmaceuticals Ltd) |
| 36649 | Hytrin 2mg tablets (Amdipharm Plc) |
| 36780 | Hytrin 5mg tablets (Amdipharm Plc) |
| 37428 | Hytrin 10mg tablets (Amdipharm Plc) |
| 40256 | Baratol 25mg tablets (Amdipharm Plc) |
| 40678 | Doxazosin 4mg tablets (Teva UK Ltd) |
| 40891 | Doxazosin 2mg tablets (IVAX Pharmaceuticals UK Ltd) |
| 41543 | Doxazosin 1mg tablets (IVAX Pharmaceuticals UK Ltd) |
| 41651 | Prazosin 500microgram Tablet (Approved Prescription Services Ltd) |
| 41652 | Prazosin 500microgram tablets (A A H Pharmaceuticals Ltd) |
| 46066 | Cardozin XL 4mg tablets (Almus Pharmaceuticals Ltd) |
| 47807 | Doxazosin xl 4mg Tablet (Hillcross Pharmaceuticals Ltd) |
| 55826 | Prazosin 5mg tablets (A A H Pharmaceuticals Ltd) |
| 520 | Losartan 25mg tablets |
| 529 | Candesartan 2mg tablets |
| 531 | Candesartan 4mg tablets |
| 575 | Valsartan 40mg capsules |
| 624 | Losartan 100mg tablets |
| 764 | Co-Diovan 80mg/12.5mg tablets (Novartis Pharmaceuticals UK Ltd) |
| 828 | Irbesartan 75mg tablets |
| 1293 | Irbesartan 150mg tablets |
| 1780 | Losartan 50mg tablets |
| 2971 | Irbesartan 300mg tablets |
| 3222 | Valsartan 80mg capsules |
| 4155 | Amias 2mg tablets (Takeda UK Ltd) |
| 4226 | Cozaar 25mg tablets (Merck Sharp & Dohme Ltd) |
| 4540 | Cozaar-Comp 50mg/12.5mg tablets (Merck Sharp & Dohme Ltd) |
| 4645 | Valsartan 160mg capsules |
| 4685 | Amias 4mg tablets (Takeda UK Ltd) |
| 4741 | Candesartan 16mg tablets |
| 4818 | Candesartan 8mg tablets |
| 5013 | Amias 8mg tablets (Takeda UK Ltd) |
| 5117 | Amias 16mg tablets (Takeda UK Ltd) |
| 5723 | Cozaar 50mg tablets (Merck Sharp & Dohme Ltd) |
| 5988 | Telmisartan 40mg tablets |
| 6217 | Olmesartan medoxomil 10mg tablets |
| 6243 | Telmisartan 20mg tablets |
| 6285 | Olmesartan medoxomil 20mg tablets |
| 6351 | Olmesartan medoxomil 40mg tablets |
| 6437 | Losartan 50mg / Hydrochlorothiazide 12.5mg tablets |
| 6518 | Diovan 160mg capsules (Novartis Pharmaceuticals UK Ltd) |
| 6877 | Co-Diovan 160mg/12.5mg tablets (Novartis Pharmaceuticals UK Ltd) |
| 6939 | Eprosartan 300mg tablets |
| 7043 | Candesartan 32mg tablets |
| 7338 | Aprovel 75mg tablets (Sanofi) |
| 9196 | Aprovel 150mg tablets (Sanofi) |
| 9745 | Teveten 300mg tablets (Abbott Healthcare Products Ltd) |
| 10316 | CoAprovel 150mg/12.5mg tablets (Sanofi) |
| 10323 | Losartan 100mg / Hydrochlorothiazide 25mg tablets |
| 11251 | Diovan 40mg capsules (Novartis Pharmaceuticals UK Ltd) |
| 11252 | Diovan 80mg capsules (Novartis Pharmaceuticals UK Ltd) |
| 11348 | Aprovel 300mg tablets (Sanofi) |
| 11448 | Irbesartan 150mg / Hydrochlorothiazide 12.5mg tablets |
| 11469 | Irbesartan 300mg / Hydrochlorothiazide 12.5mg tablets |
| 11526 | CoAprovel 300mg/12.5mg tablets (Sanofi) |
| 11864 | Valsartan 160mg / Hydrochlorothiazide 12.5mg tablets |
| 12836 | Eprosartan 600mg tablets |
| 12874 | Telmisartan 80mg tablets |
| 13123 | Eprosartan 400mg tablets |
| 13821 | Micardis 40mg tablets (Boehringer Ingelheim Ltd) |
| 14283 | Valsartan 160mg / Hydrochlorothiazide 25mg tablets |
| 14738 | Hydrochlorothiazide with losartan 12.5mg with 50mg Tablet |
| 14870 | Telmisartan 40mg / Hydrochlorothiazide 12.5mg tablets |
| 14943 | Valsartan 40mg tablets |
| 14965 | Cozaar 100mg tablets (Merck Sharp & Dohme Ltd) |
| 14983 | Olmetec 10mg tablets (Daiichi Sankyo UK Ltd) |
| 16060 | Valsartan 80mg / Hydrochlorothiazide 12.5mg tablets |
| 16161 | Telmisartan 80mg / Hydrochlorothiazide 12.5mg tablets |
| 16285 | Teveten 400mg tablets (Abbott Healthcare Products Ltd) |
| 16371 | Teveten 600mg tablets (Abbott Healthcare Products Ltd) |
| 17545 | Micardis 80mg tablets (Boehringer Ingelheim Ltd) |
| 17686 | Micardis 20mg tablets (Boehringer Ingelheim Ltd) |
| 17689 | MicardisPlus 80mg/12.5mg tablets (Boehringer Ingelheim Ltd) |
| 18200 | Olmesartan medoxomil 20mg / Hydrochlorothiazide 12.5mg tablets |
| 18202 | MicardisPlus 40mg/12.5mg tablets (Boehringer Ingelheim Ltd) |
| 18903 | Olmesartan medoxomil 20mg / Hydrochlorothiazide 25mg tablets |
| 18910 | Olmetec 20mg tablets (Daiichi Sankyo UK Ltd) |
| 20117 | Olmetec 40mg tablets (Daiichi Sankyo UK Ltd) |
| 21423 | Cozaar-Comp 100mg/25mg tablets (Merck Sharp & Dohme Ltd) |
| 23456 | Hydrochlorothiazide with valsartan 25mg with 160mg Tablet |
| 24268 | Hydrochlorothiazide with valsartan 12.5mg with 80mg Tablet |
| 24484 | Hydrochlorothiazide with valsartan 12.5mg with 160mg Tablet |
| 25382 | Co-Diovan 160mg/25mg tablets (Novartis Pharmaceuticals UK Ltd) |
| 27520 | Olmetec Plus 20mg/25mg tablets (Daiichi Sankyo UK Ltd) |
| 29634 | Olmetec Plus 20mg/12.5mg tablets (Daiichi Sankyo UK Ltd) |
| 31072 | Amias 32mg tablets (Takeda UK Ltd) |
| 35196 | CoAprovel 300mg/25mg tablets (Sanofi) |
| 35380 | Hydrochlorothiazide with olmesartan medoxomil 12.5mg with 20mg tablet |
| 35481 | Irbesartan 300mg / Hydrochlorothiazide 25mg tablets |
| 36939 | Irbesartan 300mg/5ml oral suspension |
| 37573 | Valsartan 320mg tablets |
| 37650 | Losartan 100mg / Hydrochlorothiazide 12.5mg tablets |
| 37747 | Cozaar-Comp 100mg/12.5mg tablets (Merck Sharp & Dohme Ltd) |
| 38367 | Hydrochlorothiazide with losartan 12.5mg with 100mg Tablet |
| 38395 | Valsartan 80mg tablets |
| 38459 | Telmisartan 80mg / Hydrochlorothiazide 25mg tablets |
| 38889 | MicardisPlus 80mg/25mg tablets (Boehringer Ingelheim Ltd) |
| 39199 | Diovan 320mg tablets (Novartis Pharmaceuticals UK Ltd) |
| 39944 | Losartan 12.5mg tablets |
| 40571 | Cozaar 12.5mg tablets (Merck Sharp & Dohme Ltd) |
| 40711 | Losartan 2.5mg/ml oral suspension sugar free |
| 41232 | Cozaar 2.5mg/ml oral suspension (Merck Sharp & Dohme Ltd) |
| 43322 | Olmesartan medoxomil 40mg / Hydrochlorothiazide 12.5mg tablets |
| 43915 | Olmetec Plus 40mg/12.5mg tablets (Daiichi Sankyo UK Ltd) |
| 44778 | Valsartan 160mg tablets |
| 51368 | Azilsartan medoxomil 80mg tablets |
| 58646 | Candesartan 4mg tablets (Actavis UK Ltd) |
| 65 | Lisinopril 10mg tablets |
| 69 | Lisinopril 20mg tablets |
| 78 | Lisinopril 5mg tablets |
| 80 | Ramipril 5mg capsules |
| 82 | Ramipril 10mg capsules |
| 97 | Perindopril erbumine 4mg tablets |
| 147 | Ramipril 1.25mg capsules |
| 196 | Enalapril 5mg tablets |
| 277 | Lisinopril 2.5mg tablets |
| 448 | Enalapril 2.5mg tablets |
| 593 | Perindopril erbumine 2mg tablets |
| 633 | Fosinopril 10mg tablets |
| 654 | Ramipril 2.5/5mg/10mg capsule |
| 709 | Ramipril 2.5mg capsules |
| 756 | Ramipril 10mg tablets |
| 761 | Ramipril 1.25mg tablets |
| 1021 | Innozide 20mg/12.5mg tablets (Merck Sharp & Dohme Ltd) |
| 1121 | Captopril 12.5mg tablets |
| 1143 | Captopril 25mg tablets |
| 1144 | Capoten 25mg tablets (Bristol-Myers Squibb Pharmaceuticals Ltd) |
| 1299 | Enalapril 10mg tablets |
| 1520 | Capozide 25mg/50mg tablets (Bristol-Myers Squibb Pharmaceuticals Ltd) |
| 1807 | Captopril 50mg tablets |
| 1904 | Enalapril 20mg tablets |
| 2927 | PERINDOPRIL/TERT-BUTYLAMINE 2 MG TAB |
| 2982 | Zestoretic 20- 20mg+12.5mg Tablet (AstraZeneca UK Ltd) |
| 3069 | Acepril 25mg tablets (Bristol-Myers Squibb Pharmaceuticals Ltd) |
| 3203 | Capozide LS Tablet (E R Squibb and Sons Ltd) |
| 3310 | Capoten 12.5mg tablets (Bristol-Myers Squibb Pharmaceuticals Ltd) |
| 3509 | ENALAPRIL MALEATE 40 MG TAB |
| 3720 | Zestril 2.5mg tablets (AstraZeneca UK Ltd) |
| 3839 | Capoten 50mg tablets (Bristol-Myers Squibb Pharmaceuticals Ltd) |
| 3929 | Quinapril 10mg tablets |
| 4103 | Trandolapril 1mg capsules |
| 4571 | Staril 10mg tablets (Bristol-Myers Squibb Pharmaceuticals Ltd) |
| 5047 | Trandolapril 2mg capsules |
| 5159 | Quinapril 20mg tablets |
| 5189 | Enalapril 20mg / Hydrochlorothiazide 12.5mg tablets |
| 5275 | Tritace 2.5mg capsules (Sanofi) |
| 5612 | Coversyl 2mg tablets (Servier Laboratories Ltd) |
| 5735 | Tritace 5mg capsules (Sanofi) |
| 5800 | Coversyl 4mg tablets (Servier Laboratories Ltd) |
| 5861 | Fosinopril 20mg tablets |
| 6078 | Perindopril erbumine 8mg tablets |
| 6200 | Tritace titration pack capsules (Sanofi) |
| 6261 | Tritace 1.25mg tablets (Sanofi) |
| 6288 | Ramipril 5mg tablets |
| 6314 | Ramipril 2.5mg tablets |
| 6359 | Zestoretic 10- 10mg+12.5mg Tablet (AstraZeneca UK Ltd) |
| 6362 | Tritace 5mg tablets (Sanofi) |
| 6364 | Tritace 2.5mg tablets (Sanofi) |
| 6408 | Tanatril 5mg tablets (Chiesi Ltd) |
| 6468 | Lisinopril 20mg / Hydrochlorothiazide 12.5mg tablets |
| 6765 | Quinapril 5mg tablets |
| 6786 | Lisinopril 10mg / Hydrochlorothiazide 12.5mg tablets |
| 6794 | Perindopril erbumine 4mg / Indapamide 1.25mg tablets |
| 6806 | Zestril 10mg tablets (AstraZeneca UK Ltd) |
| 6807 | Zestril 5mg tablets (AstraZeneca UK Ltd) |
| 7314 | Accupro 5mg tablets (Pfizer Ltd) |
| 7419 | Trandolapril 500microgram capsules |
| 8025 | Gopten 1mg capsules (Abbott Laboratories Ltd) |
| 8026 | Gopten 2mg capsules (Abbott Laboratories Ltd) |
| 8105 | Innovace 20mg tablets (Merck Sharp & Dohme Ltd) |
| 8106 | Innovace 2.5mg tablets (Merck Sharp & Dohme Ltd) |
| 8268 | Zestril 20mg tablets (AstraZeneca UK Ltd) |
| 8800 | Innovace 5mg tablets (Merck Sharp & Dohme Ltd) |
| 8830 | Innovace 10mg tablets (Merck Sharp & Dohme Ltd) |
| 9646 | Tritace 1.25mg capsules (Aventis Pharma) |
| 9693 | Tritace 10mg capsules (Sanofi) |
| 9731 | Quinapril 40mg tablets |
| 9764 | Carace 20 Tablet (Bristol-Myers Squibb Pharmaceuticals Ltd) |
| 9915 | Tritace 10mg tablets (Sanofi) |
| 9948 | Trandolapril 4mg capsules |
| 10882 | Carace 2.5mg tablets (Bristol-Myers Squibb Pharmaceuticals Ltd) |
| 10902 | Captopril 50mg with Hydrochlorothiazide 25mg tablets |
| 11133 | Hydrochlorothiazide with captopril 25mg with 50mg Tablet |
| 11197 | Innovace melt 5mg Wafer (Merck Sharp & Dohme Ltd) |
| 11351 | Co-zidocapt 25mg/50mg tablets |
| 11561 | Co-zidocapt 12.5mg/25mg tablets |
| 11641 | Captopril 25mg with Hydrochlorothiazide 12.5mg tablets |
| 11937 | Ramipril 2.5mg/5ml oral suspension |
| 11983 | Perindopril erbumine 4mg/5ml oral suspension |
| 11987 | Lisinopril 5mg/5ml oral solution |
| 12313 | Carace 20mg tablets (Bristol-Myers Squibb Pharmaceuticals Ltd) |
| 12411 | Cilazapril 500microgram tablets |
| 12412 | Cilazapril 2.5mg tablets |
| 12574 | Cilazapril 1mg tablets |
| 12815 | Tanatril 10mg tablets (Chiesi Ltd) |
| 12858 | Imidapril 10mg tablets |
| 13026 | Cilazapril 5mg tablets |
| 13589 | Staril 20mg tablets (Bristol-Myers Squibb Pharmaceuticals Ltd) |
| 13755 | Enalapril 10mg wafer |
| 14228 | Coversyl Plus tablets (Servier Laboratories Ltd) |
| 14387 | Carace 5mg tablets (Bristol-Myers Squibb Pharmaceuticals Ltd) |
| 14477 | Accupro 10mg tablets (Pfizer Ltd) |
| 14478 | Accupro 20mg tablets (Pfizer Ltd) |
| 14960 | Coversyl 8mg tablets (Servier Laboratories Ltd) |
| 15031 | Accuretic 12.5mg/10mg tablets (Pfizer Ltd) |
| 15085 | Innovace Titration pack (Merck Sharp & Dohme Ltd) |
| 15096 | Accupro 40mg tablets (Pfizer Ltd) |
| 15108 | Quinapril 10mg / Hydrochlorothiazide 12.5mg tablets |
| 15135 | Hydrochlorothiazide with captopril 12.5mg with 25mg Tablet |
| 15605 | Cilazapril 250micrograms tablets |
| 15958 | Captopril 2mg tablets |
| 16196 | Vascace 5mg tablets (Roche Products Ltd) |
| 16197 | Vascace 2.5mg tablets (Roche Products Ltd) |
| 16212 | Vascace 1mg tablets (Roche Products Ltd) |
| 16701 | Carace 10mg tablets (Bristol-Myers Squibb Pharmaceuticals Ltd) |
| 16708 | Enalapril titration pack |
| 16710 | Gopten 500microgram capsules (Abbott Laboratories Ltd) |
| 16924 | Imidapril 5mg tablets |
| 17120 | Moexipril 15mg tablets |
| 17655 | Carace 10 Tablet (Bristol-Myers Squibb Pharmaceuticals Ltd) |
| 18219 | Imidapril 20mg tablets |
| 18263 | Acezide 25mg/50mg tablets (Bristol-Myers Squibb Pharmaceuticals Ltd) |
| 18269 | Acepril 12.5mg tablets (Bristol-Myers Squibb Pharmaceuticals Ltd) |
| 18325 | Acepril 50mg tablets (Bristol-Myers Squibb Pharmaceuticals Ltd) |
| 19198 | Lisinopril 20mg tablets (Teva UK Ltd) |
| 19204 | Lisinopril 5mg tablets (Teva UK Ltd) |
| 19223 | Lisinopril 10mg tablets (Teva UK Ltd) |
| 20188 | Enalapril 2.5mg wafer |
| 20975 | Lisinopril 7.5mg/5ml oral suspension |
| 21053 | Vascace 500microgram tablets (Roche Products Ltd) |
| 22439 | Ednyt 20mg Tablet (Dominion Pharma) |
| 22708 | Enalapril 5mg wafer |
| 23382 | CARACE (SPECIAL COMPLIANCE PACK) |
| 24693 | CARACE (SPECIAL COMPLIANCE PACK) |
| 26995 | Kaplon 25mg tablets (Teva UK Ltd) |
| 27871 | Innovace melt 10mg Wafer (Merck Sharp & Dohme Ltd) |
| 28586 | Lopace 5mg capsules (Discovery Pharmaceuticals Ltd) |
| 28725 | Perdix 15mg tablets (UCB Pharma Ltd) |
| 28902 | Odrik 2mg capsules (Aventis Pharma) |
| 29130 | Gopten 4mg capsules (Abbott Laboratories Ltd) |
| 29627 | Lopace 2.5mg capsules (Discovery Pharmaceuticals Ltd) |
| 30921 | Lisinopril 2.5mg tablets (Teva UK Ltd) |
| 32241 | Enalapril 10mg tablets (A A H Pharmaceuticals Ltd) |
| 32560 | Tanatril 20mg tablets (Chiesi Ltd) |
| 32857 | Ramipril 1.25mg capsules (Teva UK Ltd) |
| 32934 | Lopace 10mg capsules (Discovery Pharmaceuticals Ltd) |
| 33078 | Enalapril 20mg tablets (A A H Pharmaceuticals Ltd) |
| 33095 | Perindopril erbumine 4mg tablets (A A H Pharmaceuticals Ltd) |
| 33894 | Ramipril 10mg capsules (Teva UK Ltd) |
| 33977 | Lisinopril 10mg tablets (Generics (UK) Ltd) |
| 34357 | Ramipril 10mg capsules (Genus Pharmaceuticals Ltd) |
| 34390 | Ramipril 5mg capsules (Genus Pharmaceuticals Ltd) |
| 34412 | Ramipril 5mg capsules (Teva UK Ltd) |
| 34429 | Ramipril 5mg capsules (Generics (UK) Ltd) |
| 34432 | Ramipril 2.5mg capsules (Genus Pharmaceuticals Ltd) |
| 34490 | Ramipril 2.5mg capsules (Teva UK Ltd) |
| 34505 | Ramipril 2.5mg capsules (Sandoz Ltd) |
| 34528 | Ramipril 2.5mg capsules (A A H Pharmaceuticals Ltd) |
| 34539 | Ramipril 5mg capsules (Sandoz Ltd) |
| 34540 | Ramipril 5mg capsules (A A H Pharmaceuticals Ltd) |
| 34562 | Captopril 25mg Tablet (IVAX Pharmaceuticals UK Ltd) |
| 34583 | Ramipril 10mg Capsule (Dexcel-Pharma Ltd) |
| 34657 | Ramipril 10mg capsules (Zentiva) |
| 34696 | Lisinopril 20mg tablets (Generics (UK) Ltd) |
| 34799 | Lisinopril 20mg tablets (Zentiva) |
| 34877 | Ramipril 10mg Capsule (Sovereign Medical Ltd) |
| 35007 | Ramipril 10mg/5ml oral suspension |
| 35302 | Captopril 12.5mg/5ml oral suspension |
| 37080 | Enalapril 5mg/5ml oral solution |
| 37087 | Enalapril 5mg/5ml oral suspension |
| 37778 | Lisinopril 5mg/5ml oral suspension |
| 37908 | Coversyl Arginine Plus 5mg/1.25mg tablets (Servier Laboratories Ltd) |
| 37930 | Perindopril arginine 5mg tablets |
| 37964 | Perindopril arginine 2.5mg tablets |
| 37965 | Coversyl Arginine 5mg tablets (Servier Laboratories Ltd) |
| 37971 | Perindopril arginine 10mg tablets |
| 37978 | Perindopril arginine 5mg / Indapamide 1.25mg tablets |
| 38026 | Coversyl Arginine 10mg tablets (Servier Laboratories Ltd) |
| 38034 | Coversyl Arginine 2.5mg tablets (Servier Laboratories Ltd) |
| 38308 | Ramipril 2.5/5mg/10mg tablet |
| 38995 | Zestoretic 20 tablets (AstraZeneca UK Ltd) |
| 39137 | Zestoretic 10 tablets (AstraZeneca UK Ltd) |
| 39147 | Carace 20 Plus tablets (Merck Sharp & Dohme Ltd) |
| 39227 | Capozide LS 12.5mg/25mg tablets (Bristol-Myers Squibb Pharmaceuticals Ltd) |
| 39242 | Carace 10 Plus tablets (Merck Sharp & Dohme Ltd) |
| 39421 | Tritace titration pack tablets (Sanofi) |
| 41417 | Enalapril 2.5mg tablets (A A H Pharmaceuticals Ltd) |
| 41522 | Lisopress 20mg tablets (Teva UK Ltd) |
| 41532 | Lisopress 5mg tablets (Teva UK Ltd) |
| 41538 | Lisopress 2.5mg tablets (Teva UK Ltd) |
| 41573 | Lisopress 10mg tablets (Teva UK Ltd) |
| 41617 | Captopril 25mg tablets (Actavis UK Ltd) |
| 41633 | Captopril 12.5mg tablets (Actavis UK Ltd) |
| 41746 | Enalapril 10mg tablets (Sandoz Ltd) |
| 42894 | Enalapril 10mg tablets (Teva UK Ltd) |
| 42901 | Enalapril 5mg tablets (Teva UK Ltd) |
| 42902 | Enalapril 20mg tablets (Teva UK Ltd) |
| 42908 | Enalapril 5mg tablets (IVAX Pharmaceuticals UK Ltd) |
| 43411 | Enalapril 5mg tablets (Sandoz Ltd) |
| 43412 | Lisinopril 2.5mg tablets (A A H Pharmaceuticals Ltd) |
| 43413 | Lisinopril 20mg tablets (A A H Pharmaceuticals Ltd) |
| 43416 | Lisinopril 10mg tablets (A A H Pharmaceuticals Ltd) |
| 43418 | Lisinopril 5mg tablets (A A H Pharmaceuticals Ltd) |
| 45217 | Enalapril 5mg tablets (Kent Pharmaceuticals Ltd) |
| 45554 | Ramipril 5mg/5ml oral solution |
| 46890 | Ramipril 5mg/5ml oral suspension |
| 47021 | Ramipril 2.5mg/5ml oral solution sugar free |
| 54986 | Perindopril erbumine 8mg/5ml oral suspension |
| 56079 | Perindopril tosilate 10mg tablets |
| 56472 | Perindopril erbumine 4mg tablets (Kent Pharmaceuticals Ltd) |
| 56473 | Perindopril erbumine 2mg tablets (Sigma Pharmaceuticals Plc) |
| 56505 | Zestril 5mg tablets (Lexon (UK) Ltd) |
| 56506 | Coversyl 2mg tablets (Dowelhurst Ltd) |
| 56508 | Coversyl 4mg tablets (Dowelhurst Ltd) |
| 56510 | Zestril 20mg tablets (Sigma Pharmaceuticals Plc) |
| 57539 | Zestoretic 10 tablets (Sigma Pharmaceuticals Plc) |
| 57944 | Perindopril tosilate 2.5mg tablets |
| 5 | Atenolol 50mg tablets |
| 24 | Atenolol 100mg tablets |
| 26 | Atenolol 25mg tablets |
| 197 | Atenolol 5mg/10ml solution for injection ampoules |
| 297 | Propranolol 10mg tablets |
| 472 | Bisoprolol 5mg tablets |
| 581 | Atenolol 50mg with Chlortalidone 12.5mg tablets |
| 594 | Bisoprolol 2.5mg tablets |
| 599 | Bisoprolol 1.25mg tablets |
| 707 | Propranolol 40mg tablets |
| 739 | Metoprolol 50mg tablets |
| 751 | Nebivolol 5mg tablets |
| 753 | Metoprolol 100mg tablets |
| 769 | Propranolol 80mg modified-release capsules |
| 786 | Sotalol 40mg tablets |
| 817 | Carvedilol 3.125mg tablets |
| 822 | Bisoprolol 1.5mg/5ml oral suspension |
| 940 | Propranolol 80mg tablets |
| 1006 | Half Inderal LA 80mg capsules (AstraZeneca UK Ltd) |
| 1048 | Inderal 80mg tablets (AstraZeneca UK Ltd) |
| 1050 | Inderal 40mg tablets (AstraZeneca UK Ltd) |
| 1124 | Tenoretic 100mg/25mg tablets (AstraZeneca UK Ltd) |
| 1288 | Tenoret 50mg/12.5mg tablets (AstraZeneca UK Ltd) |
| 1290 | Bisoprolol 10mg tablets |
| 1295 | Labetalol 400mg tablets |
| 1333 | Oxprenolol 40mg tablets |
| 1334 | Oxprenolol 160mg modified-release tablets |
| 1448 | Propranolol 160mg modified-release capsules |
| 1572 | Sotalol 80mg tablets |
| 1597 | Labetalol 100mg tablets |
| 1684 | Beta-Adalat modified-release capsules (Bayer Plc) |
| 1788 | Atenolol 100mg with Chlortalidone 25mg tablets |
| 2361 | Trasicor 80mg Tablet (Novartis Pharmaceuticals UK Ltd) |
| 2414 | Inderal 10mg tablets (AstraZeneca UK Ltd) |
| 2432 | Tenormin LS 50mg tablets (AstraZeneca UK Ltd) |
| 2432 | Tenormin LS 50mg tablets (AstraZeneca UK Ltd) |
| 2499 | Nadolol 80mg tablets |
| 2587 | Tenormin 100mg tablets (AstraZeneca UK Ltd) |
| 2587 | Tenormin 100mg tablets (AstraZeneca UK Ltd) |
| 2590 | Tenormin 25mg tablets (AstraZeneca UK Ltd) |
| 2629 | Carvedilol 12.5mg tablets |
| 2775 | Labetalol 200mg tablets |
| 2780 | Oxprenolol 80mg tablets |
| 3005 | Inderal LA 160mg capsules (AstraZeneca UK Ltd) |
| 3041 | SOTALOL HCl 40 MG INJ |
| 3087 | Propranolol 40mg/5ml oral solution sugar free |
| 3167 | Propranolol 160mg tablets |
| 3344 | Betaloc 100mg tablets (AstraZeneca UK Ltd) |
| 3474 | Betaloc-SA 200mg tablets (AstraZeneca UK Ltd) |
| 3516 | Oxprenolol 20mg tablets |
| 3526 | Amiloride with atenolol with hydrochlorothiazide capsules |
| 3588 | Monocor 5mg tablets (Wyeth Pharmaceuticals) |
| 3691 | Sotalol 160mg with hydrochlorothiazide 25mg tablet |
| 3748 | Oxprenolol 160mg Tablet |
| 3827 | Propanix 40mg Tablet (Ashbourne Pharmaceuticals Ltd) |
| 4004 | Sotacor 80mg tablets (Bristol-Myers Squibb Pharmaceuticals Ltd) |
| 4021 | PROPRANOLOL 20 MG TAB |
| 4025 | Slow-Trasicor 160mg tablets (Amdipharm Plc) |
| 4265 | Celectol 200mg Tablet (Pantheon Healthcare Ltd) |
| 4410 | Carvedilol 6.25mg tablets |
| 4429 | Trasidrex modified-release tablets (Mercury Pharma Group Ltd) |
| 4542 | Atenolol 50mg / Nifedipine 20mg modified-release capsules |
| 4588 | Visken 5mg Tablet (Sovereign Medical Ltd) |
| 4605 | Moducren tablets (Merck Sharp & Dohme Ltd) |
| 4725 | Labetalol 50mg tablets |
| 4771 | Emcor LS 5mg tablets (Merck Serono Ltd) |
| 4796 | Inderetic 80mg/2.5mg capsules (AstraZeneca UK Ltd) |
| 4983 | Atenolol with amiloride and hydrochlorothiazide capsules |
| 5284 | Pindolol 5mg tablets |
| 5330 | Corgaretic 40mg tablets (Sanofi-Synthelabo Ltd) |
| 5478 | Propranolol 10mg/5ml oral solution sugar free |
| 5713 | Bisoprolol 7.5mg tablets |
| 5721 | Co-tenidone 100mg/25mg tablets |
| 5858 | Beta-Cardone 40mg tablets (Focus Pharmaceuticals Ltd) |
| 5968 | Monocor 10mg tablets (Wyeth Pharmaceuticals) |
| 6066 | Atenolol 25mg/5ml oral solution sugar free |
| 6751 | Beta-Cardone 80mg tablets (Focus Pharmaceuticals Ltd) |
| 7049 | Carvedilol 25mg tablets |
| 7066 | Metoprolol 100mg / Hydrochlorothiazide 12.5mg tablets |
| 7091 | Bisoprolol 3.75mg tablets |
| 7429 | Tenormin 5mg/10ml solution for injection ampoules (AstraZeneca UK Ltd) |
| 7474 | Trasicor 20mg Tablet (Novartis Pharmaceuticals UK Ltd) |
| 7491 | LABETALOL TAB |
| 7528 | Nebilet 5mg tablets (A. Menarini Farmaceutica Internazionale SRL) |
| 7543 | Kalten capsules (M & A Pharmachem Ltd) |
| 7620 | Acebutolol 400mg tablets |
| 7852 | Blocadren 10mg Tablet (Merck Sharp & Dohme Ltd) |
| 7853 | Timolol 10mg tablets |
| 7974 | Celiprolol 400mg tablets |
| 8023 | Sectral 400mg tablets (Sanofi) |
| 8061 | Sotalol 80mg with hydrochlorothiazide 12.5mg tablet |
| 8068 | Metoprolol 200mg modified-release tablets |
| 8071 | Betaloc 50mg tablets (AstraZeneca UK Ltd) |
| 8113 | Acebutolol 200mg capsules |
| 8147 | Lopresoretic Tablet (Novartis Pharmaceuticals UK Ltd) |
| 8172 | Acebutolol 100mg capsules |
| 8189 | Secadrex 200mg/12.5mg tablets (Sanofi) |
| 8262 | Celiprolol 200mg tablets |
| 8290 | Trasicor 40mg Tablet (Novartis Pharmaceuticals UK Ltd) |
| 8331 | Inderal 160mg Tablet (AstraZeneca UK Ltd) |
| 8369 | Inderex 160mg/5mg modified-release capsules (AstraZeneca UK Ltd) |
| 8555 | Sectral 200mg capsules (Sanofi) |
| 8623 | Prestim Tablet (ICN Pharmaceuticals France S.A.) |
| 8642 | Tenif 50mg/20mg modified-release capsules (AstraZeneca UK Ltd) |
| 8673 | Oxprenolol with cyclopenthiazide 160mg+0.25mg Modified-release tablet |
| 8707 | Trandate 200mg tablets (Focus Pharmaceuticals Ltd) |
| 8765 | ATENOLOL/CHLORTHALIDONE 50 MG TAB |
| 8788 | TIMOLOL 10MG/BENDROFLUAZIDE 2.5MG TAB |
| 8807 | Trandate 400mg tablets (Focus Pharmaceuticals Ltd) |
| 8935 | Nadolol 40mg tablets |
| 8987 | Propranolol 160mg modified-release / Bendroflumethiazide 5mg capsules |
| 9016 | Trandate 100mg tablets (Focus Pharmaceuticals Ltd) |
| 9143 | Viskaldix tablets (Amdipharm Plc) |
| 9178 | Atenolol 25mg / Bendroflumethiazide 1.25mg capsules |
| 9185 | Propranolol 80mg/5ml oral solution |
| 9273 | Trandate 50mg tablets (Focus Pharmaceuticals Ltd) |
| 9292 | Sotalol 160mg tablets |
| 9783 | Co-tenidone 50mg/12.5mg tablets |
| 10191 | Atenix 50 tablets (Ashbourne Pharmaceuticals Ltd) |
| 10429 | Lopresor 50mg Tablet (Novartis Pharmaceuticals UK Ltd) |
| 10627 | Co-Betaloc tablets (Pfizer Ltd) |
| 10716 | Corgard 80mg tablets (Sanofi) |
| 10777 | Trasicor 160mg Tablet (Novartis Pharmaceuticals UK Ltd) |
| 10892 | Emcor 10mg tablets (Merck Serono Ltd) |
| 11380 | Sotacor 160mg tablets (Bristol-Myers Squibb Pharmaceuticals Ltd) |
| 11711 | Propranolol 50mg/5ml oral solution |
| 11793 | Metoprolol 50mg/5ml oral suspension |
| 12037 | Betim 10mg Tablet (ICN Pharmaceuticals France S.A.) |
| 12054 | Propranolol 80mg / Bendroflumethiazide 2.5mg capsules |
| 12119 | SOTALOL HCl S/R 80 MG TAB |
| 12141 | Betaxolol 20mg tablets |
| 12296 | Sectral 100mg capsules (Sanofi) |
| 12456 | Sotazide Tablet (Bristol-Myers Squibb Pharmaceuticals Ltd) |
| 12495 | Berkolol 10mg Tablet (Berk Pharmaceuticals Ltd) |
| 12517 | Timolol maleate with bendroflumethiazide 20mg + 5mg Tablet |
| 12519 | Kerlone 20mg tablets (Sanofi-Synthelabo Ltd) |
| 12651 | Timolol 10mg / Bendroflumethiazide 2.5mg tablets |
| 13051 | Sotalol 200mg tablets |
| 13394 | Tenormin 25mg/5ml syrup (AstraZeneca UK Ltd) |
| 13415 | Corgard 40mg tablets (Sanofi-Synthelabo Ltd) |
| 13499 | Lopresor 100mg Tablet (Novartis Pharmaceuticals UK Ltd) |
| 13526 | Atenix Co 100 tablets (Ashbourne Pharmaceuticals Ltd) |
| 13871 | Co-prenozide 160mg/0.25mg modified-release tablets |
| 14030 | Cardicor 2.5mg tablets (Merck Serono Ltd) |
| 14057 | Pindolol 10mg / Clopamide 5mg tablets |
| 14058 | Cardicor 1.25mg tablets (Merck Serono Ltd) |
| 14117 | Eucardic 3.125mg tablets (Roche Products Ltd) |
| 14126 | Acebutolol 200mg / Hydrochlorothiazide 12.5mg tablets |
| 14146 | Eucardic 6.25mg tablets (Roche Products Ltd) |
| 14438 | Corgaretic 80mg tablets (Sanofi-Synthelabo Ltd) |
| 14552 | Propanix 10mg Tablet (Ashbourne Pharmaceuticals Ltd) |
| 14673 | Pindolol 15mg tablets |
| 14808 | Bedranol SR 80mg capsules (Sandoz Ltd) |
| 15042 | Tolerzide Tablet (Bristol-Myers Squibb Pharmaceuticals Ltd) |
| 15117 | Nifedipine with atenolol 20mg + 50mg Capsule |
| 15176 | Totamol 50mg Tablet (C P Pharmaceuticals Ltd) |
| 15488 | Metoprolol tartrate with chlortalidone Tablet |
| 15619 | Half-betadur cr 80mg Capsule (Monmouth Pharmaceuticals Ltd) |
| 15730 | Totamol 100mg Tablet (C P Pharmaceuticals Ltd) |
| 16645 | Labrocol 400mg Tablet (Lagap) |
| 16669 | LOPRESOR SR 200 MG TAB |
| 16776 | Celectol 400mg Tablet (Pantheon Healthcare Ltd) |
| 16786 | Chlortalidone 25mg with Atenolol 100mg tablets |
| 17149 | Monozide 10 tablets (Wyeth Pharmaceuticals) |
| 17322 | Atenix 25 tablets (Ashbourne Pharmaceuticals Ltd) |
| 17462 | Bisoprolol 10mg / Hydrochlorothiazide 6.25mg tablets |
| 17615 | Cardicor 5mg tablets (Merck Serono Ltd) |
| 18185 | Cardicor 7.5mg tablets (Merck Serono Ltd) |
| 18287 | Co-Betaloc SA tablets (Pfizer Ltd) |
| 18414 | Eucardic 12.5mg tablets (Roche Products Ltd) |
| 18743 | Tenben 25mg/1.25mg capsules (Galen Ltd) |
| 18950 | Totamol 25mg Tablet (C P Pharmaceuticals Ltd) |
| 19055 | Chlortalidone 12.5mg with Atenolol 50mg tablets |
| 19142 | Bendroflumethiazide 2.5mg with Timolol maleate 10mg tablets |
| 19172 | Atenolol 25mg tablets (IVAX Pharmaceuticals UK Ltd) |
| 19178 | Bisoprolol 10mg tablets (Ranbaxy (UK) Ltd) |
| 19182 | Atenolol 50mg tablets (IVAX Pharmaceuticals UK Ltd) |
| 19191 | Atenolol 100mg tablets (Teva UK Ltd) |
| 19437 | Eucardic 25mg tablets (Roche Products Ltd) |
| 19687 | MODUCREN |
| 19853 | Cardicor 3.75mg tablets (Merck Serono Ltd) |
| 19858 | Cardicor 10mg tablets (Merck Serono Ltd) |
| 19998 | Trandate 100mg/20ml solution for injection ampoules (Focus Pharmaceuticals Ltd) |
| 20012 | Visken 15mg Tablet (Sovereign Medical Ltd) |
| 20082 | Lopresor SR 200mg tablets (Recordati Pharmaceuticals Ltd) |
| 20093 | Metoprolol 200mg modified-release / Hydrochlorothiazide 25mg tablets |
| 20468 | Half Beta-Prograne 80mg modified-release capsules (Tillomed Laboratories Ltd) |
| 20502 | Atenix 100 tablets (Ashbourne Pharmaceuticals Ltd) |
| 20728 | Atenamin 25mg Tablet (OPD Pharm) |
| 21025 | Prestim forte Tablet (LEO Pharma) |
| 21133 | Atenamin 50mg Tablet (OPD Pharm) |
| 21182 | Hydrochlorothiazide with timolol and amiloride 25mg with 10mg with 2.5mg Tablet |
| 21838 | Propanix 80mg Tablet (Ashbourne Pharmaceuticals Ltd) |
| 21839 | Berkolol 80mg Tablet (Berk Pharmaceuticals Ltd) |
| 21866 | Berkolol 40mg Tablet (Berk Pharmaceuticals Ltd) |
| 21873 | Atenix Co 50 tablets (Ashbourne Pharmaceuticals Ltd) |
| 22208 | Half propanix la 80mg Modified-release capsule (Ashbourne Pharmaceuticals Ltd) |
| 22634 | PROPRANOLOL 10 MG SUS |
| 23134 | Nadolol 40mg / Bendroflumethiazide 5mg tablets |
| 23326 | Betadur cr 160mg Modified-release capsule (Monmouth Pharmaceuticals Ltd) |
| 24083 | Bisoprolol 5mg tablets (Teva UK Ltd) |
| 24094 | Trasicor 40mg tablets (Amdipharm Plc) |
| 24191 | Antipressan 50mg tablets (Teva UK Ltd) |
| 24195 | Antipressan 100mg tablets (Teva UK Ltd) |
| 24461 | Betaloc I.V. 5mg/5ml solution for injection ampoules (AstraZeneca UK Ltd) |
| 25359 | Rapranol SR 160mg capsules (Ranbaxy (UK) Ltd) |
| 25363 | Prestim tablets (Meda Pharmaceuticals Ltd) |
| 25367 | Rapranol SR 80mg capsules (Ranbaxy (UK) Ltd) |
| 26211 | Antipressan 25mg tablets (Teva UK Ltd) |
| 26228 | Propanix LA 160mg Modified-release capsule (Ashbourne Pharmaceuticals Ltd) |
| 26229 | Beta-Prograne 160mg modified-release capsules (Tillomed Laboratories Ltd) |
| 26248 | Tenchlor 100mg/25mg tablets (Teva UK Ltd) |
| 27700 | Propranolol 40mg tablets (Actavis UK Ltd) |
| 27727 | Sotalol 2mg/ml injection |
| 28128 | Propranolol 80mg Modified-release capsule (Actavis UK Ltd) |
| 28177 | Hydrochlorothiazide with atenolol and amiloride Capsule |
| 29180 | Trasicor 80mg tablets (Amdipharm Plc) |
| 29368 | Atenolol 25mg tablets (Teva UK Ltd) |
| 29398 | Atenamin 100mg Tablet (OPD Pharm) |
| 29610 | Betim 10mg tablets (Meda Pharmaceuticals Ltd) |
| 29762 | Mepranix 50mg Tablet (Ashbourne Pharmaceuticals Ltd) |
| 31708 | Co-tenidone 50mg/12.5mg tablets (Actavis UK Ltd) |
| 31934 | Atenolol 100mg tablets (IVAX Pharmaceuticals UK Ltd) |
| 32114 | Bisoprolol 5mg tablets (Generics (UK) Ltd) |
| 32552 | Congescor 2.5mg tablets (Tillomed Laboratories Ltd) |
| 32836 | Metoprolol 50mg tablets (Generics (UK) Ltd) |
| 33079 | Atenolol 100mg tablets (Generics (UK) Ltd) |
| 33085 | Atenolol 100mg tablets (A A H Pharmaceuticals Ltd) |
| 33092 | Atenolol 50mg tablets (A A H Pharmaceuticals Ltd) |
| 33650 | Atenolol 50mg tablets (Generics (UK) Ltd) |
| 33657 | Atenolol 25mg tablets (A A H Pharmaceuticals Ltd) |
| 33839 | Bisoprolol 10mg tablets (Actavis UK Ltd) |
| 33850 | Atenolol 50mg tablets (Actavis UK Ltd) |
| 33909 | Congescor 1.25mg tablets (Tillomed Laboratories Ltd) |
| 34012 | Co-tenidone 100mg/25mg tablets (IVAX Pharmaceuticals UK Ltd) |
| 34034 | Co-tenidone 50mg/12.5mg tablets (IVAX Pharmaceuticals UK Ltd) |
| 34092 | Metoprolol 100mg tablets (Teva UK Ltd) |
| 34094 | Metoprolol 50mg tablets (A A H Pharmaceuticals Ltd) |
| 34125 | Metoprolol 100mg tablets (A A H Pharmaceuticals Ltd) |
| 34177 | Labetalol 100mg tablets (A A H Pharmaceuticals Ltd) |
| 34365 | Atenolol 50mg tablets (Teva UK Ltd) |
| 34378 | Propranolol 10mg tablets (A A H Pharmaceuticals Ltd) |
| 34407 | Metoprolol 50mg tablets (Teva UK Ltd) |
| 34430 | Metoprolol 50mg tablets (Actavis UK Ltd) |
| 34443 | Atenolol 50mg tablets (Wockhardt UK Ltd) |
| 34520 | Sotalol 80mg tablets (Generics (UK) Ltd) |
| 34585 | Atenolol 25mg tablets (Sandoz Ltd) |
| 34740 | Carvedilol 6.25mg tablets (Actavis UK Ltd) |
| 34754 | Atenolol 100mg tablets (Sandoz Ltd) |
| 34783 | Propranolol 10mg tablets (Actavis UK Ltd) |
| 34804 | Propranolol 10mg tablets (Teva UK Ltd) |
| 34821 | Bisoprolol 10mg tablets (Generics (UK) Ltd) |
| 34825 | Co-tenidone 50mg/12.5mg tablets (Teva UK Ltd) |
| 34854 | Metoprolol 100mg tablets (Actavis UK Ltd) |
| 34867 | Propranolol 80mg Capsule (IVAX Pharmaceuticals UK Ltd) |
| 34890 | Metoprolol 50mg Tablet (Berk Pharmaceuticals Ltd) |
| 34899 | Co-tenidone 100mg/25mg tablets (A A H Pharmaceuticals Ltd) |
| 34925 | Metoprolol 50mg tablets (Sandoz Ltd) |
| 34963 | Bisoprolol 5mg tablets (Actavis UK Ltd) |
| 35054 | Celectol 200mg tablets (Zentiva) |
| 35062 | Trasicor 20mg tablets (Amdipharm Plc) |
| 35695 | Visken 5mg tablets (Amdipharm Plc) |
| 35940 | Celectol 400mg tablets (Zentiva) |
| 36261 | Atenolol 50mg tablets (Tillomed Laboratories Ltd) |
| 36576 | Propranolol 10mg tablets (Generics (UK) Ltd) |
| 39646 | Bisoprolol 0.625mg/5ml oral solution |
| 40167 | Metoprolol 100mg tablets (IVAX Pharmaceuticals UK Ltd) |
| 40761 | Nebivolol 2.5mg tablets |
| 41555 | Propranolol 40mg tablets (A A H Pharmaceuticals Ltd) |
| 41572 | Co-tenidone 100mg/25mg tablets (Teva UK Ltd) |
| 41740 | Celiprolol 200mg tablets (Teva UK Ltd) |
| 42795 | Celiprolol 200mg tablets (Generics (UK) Ltd) |
| 44000 | Bisoprolol 2.5mg/5ml oral suspension |
| 44858 | Atenolol 25mg tablets (Actavis UK Ltd) |
| 45877 | Beta-Prograne 160mg modified-release capsules (Teva UK Ltd) |
| 46740 | Lopresor 100mg tablets (Recordati Pharmaceuticals Ltd) |
| 46935 | Carvedilol 3.125mg tablets (Actavis UK Ltd) |
| 46936 | Carvedilol 3.125mg tablets (A A H Pharmaceuticals Ltd) |
| 47107 | Carvedilol 5mg/5ml oral suspension |
| 47536 | Metoprolol tartrate 12.5mg/5ml Oral suspension |
| 47907 | Bedranol SR 160mg capsules (Almus Pharmaceuticals Ltd) |
| 51447 | Metoprolol 12.5mg/5ml oral suspension |
| 56486 | Monocor 10mg tablets (Dowelhurst Ltd) |
| 57063 | Bedranol SR 80mg capsules (Almus Pharmaceuticals Ltd) |
| 57573 | Celectol 200mg tablets (Dowelhurst Ltd) |
| 58109 | Bisoprolol 1.25mg/5ml oral suspension |
| 29 | Amlodipine besilate 5mg tablets |
| 71 | Amlodipine besilate 10mg tablets |
| 219 | Diltiazem 120mg modified-release tablets |
| 269 | Nifedipine 5mg capsules |
| 410 | Nifedipine 10mg modified-release tablets |
| 452 | Nifedipine 10mg capsules |
| 491 | Felodipine 2.5mg modified-release tablets |
| 501 | Felodipine 5mg modified-release tablets |
| 517 | Adizem sr 120mg Modified-release capsule (Napp Pharmaceuticals Ltd) |
| 536 | Tildiem la 200mg Modified-release capsule (Sanofi) |
| 541 | Adalat LA 20 tablets (Bayer Plc) |
| 568 | Felodipine 10mg modified-release tablets |
| 636 | Diltiazem 60mg modified-release capsules |
| 662 | Adalat 5mg capsules (Bayer Plc) |
| 700 | Vera-Til SR 120mg tablets (Tillomed Laboratories Ltd) |
| 729 | Amlodipine maleate 5mg tablets |
| 737 | Nifedipine 20mg modified-release capsules |
| 749 | Amlodipine 5mg tablets |
| 793 | Adizem xl 240mg Capsule (Napp Pharmaceuticals Ltd) |
| 939 | Tildiem Retard 90mg tablets (Sanofi) |
| 1118 | Verapamil 40mg tablets |
| 1120 | Verapamil 80mg tablets |
| 1130 | Viazem XL 300mg capsules (Thornton & Ross Ltd) |
| 1262 | Nifedipine 12 20mg Modified-release tablet |
| 1289 | Tildiem Retard 120mg tablets (Sanofi) |
| 1298 | Verapamil 240mg modified-release tablets |
| 1300 | Nifensar xl 20mg Modified-release tablet (Rhone-Poulenc Rorer Ltd) |
| 1449 | Nifedipine 24 30mg Modified-release tablet |
| 1529 | Posicor 50mg Tablet (Roche Products Ltd) |
| 1538 | Diltiazem 60mg tablets |
| 1574 | Verapamil 120mg modified-release capsules |
| 1686 | Diltiazem 90mg modified-release capsules |
| 1747 | Verapamil 120mg tablets |
| 1748 | Cordilox 120mg tablets (IVAX Pharmaceuticals UK Ltd) |
| 1836 | Diltiazem 60mg modified-release tablets |
| 1854 | Adalat la 30mg Tablet (Bayer Plc) |
| 1995 | Diltiazem 12hr 120mg modified-release capsules |
| 2280 | Adalat retard 10mg tablets (Bayer Plc) |
| 2343 | Adalat retard 20mg tablets (Bayer Plc) |
| 2453 | Diltiazem 60mg modified-release capsules |
| 2521 | Adalat 10mg capsules (Bayer Plc) |
| 2528 | Slozem 120mg capsules (Merck Serono Ltd) |
| 2592 | Viazem XL 120mg capsules (Thornton & Ross Ltd) |
| 2605 | Nifedipine 10mg modified-release capsules |
| 2663 | Diltiazem 240mg modified-release capsules |
| 2686 | Dilzem xl mr 240mg Modified-release capsule (Elan Pharma) |
| 2746 | Coracten SR 10mg capsules (UCB Pharma Ltd) |
| 2811 | Adizem sr 180mg Modified-release capsule (Napp Pharmaceuticals Ltd) |
| 2888 | Tildiem 60mg modified-release tablets (Sanofi) |
| 2926 | Nicardipine 20mg capsules |
| 3057 | Securon 120mg tablets (Abbott Laboratories Ltd) |
| 3061 | Diltiazem 12hr 180mg modified-release capsules |
| 3118 | Adizem sr 90mg Modified-release capsule (Napp Pharmaceuticals Ltd) |
| 3221 | Lacidipine 4mg tablets |
| 3302 | Cardene SR 30mg capsules (Astellas Pharma Ltd) |
| 3342 | Securon SR 240mg tablets (Abbott Laboratories Ltd) |
| 3343 | Half Securon SR 120mg tablets (Abbott Laboratories Ltd) |
| 3370 | Dilzem xl mr 120mg Modified-release capsule (Elan Pharma) |
| 3676 | Dilzem xl mr 180mg Modified-release capsule (Elan Pharma) |
| 3711 | Adipine MR 20 tablets (Chiesi Ltd) |
| 3712 | Coracten XL 30mg capsules (UCB Pharma Ltd) |
| 3917 | Istin 5mg tablets (Pfizer Ltd) |
| 3930 | Nifedipine 60mg modified-release tablets |
| 3931 | Posicor 100mg Tablet (Roche Products Ltd) |
| 3943 | Verapamil 240mg modified-release capsules |
| 4227 | Adalat la 60mg Tablet (Bayer Plc) |
| 4239 | Adipine MR 10 tablets (Chiesi Ltd) |
| 4308 | Dilzem sr 90mg Capsule (Elan Pharma) |
| 4408 | Slozem 240mg capsules (Merck Serono Ltd) |
| 4635 | Diltiazem 200mg modified-release capsules |
| 4732 | Diltiazem 90mg modified-release tablets |
| 4808 | Diltiazem 240mg modified-release capsules |
| 4852 | Adizem sr 120mg Modified-release tablet (Napp Pharmaceuticals Ltd) |
| 4856 | Coracten SR 20mg capsules (UCB Pharma Ltd) |
| 4923 | Diltiazem 24hr 180mg modified-release capsules |
| 4939 | Coracten XL 60mg capsules (UCB Pharma Ltd) |
| 5054 | Angitil SR 180 capsules (Chiesi Ltd) |
| 5158 | Lacidipine 2mg tablets |
| 5162 | Nifedipine 30mg modified-release capsules |
| 5181 | Angiopine MR 20mg tablets (Ashbourne Pharmaceuticals Ltd) |
| 5194 | Dilzem sr 120mg Capsule (Elan Pharma) |
| 5234 | Slozem 180mg capsules (Merck Serono Ltd) |
| 5277 | Fortipine LA 40 tablets (Mercury Pharma Group Ltd) |
| 5296 | Tildiem la 300mg Modified-release capsule (Sanofi) |
| 5326 | Diltiazem 24hr 300mg modified-release capsules |
| 5348 | Diltiazem 300mg modified-release capsules |
| 5477 | Nicardipine 30mg modified-release capsules |
| 5513 | Dilzem sr 60mg Capsule (Elan Pharma) |
| 5570 | Zanidip 10mg tablets (Recordati Pharmaceuticals Ltd) |
| 5593 | Lercanidipine 10mg tablets |
| 5806 | Tensipine MR 20 tablets (Thornton & Ross Ltd) |
| 5914 | Istin 10mg tablets (Pfizer Ltd) |
| 6309 | Adizem xl 300mg Capsule (Napp Pharmaceuticals Ltd) |
| 6477 | Amlodipine maleate 10mg tablets |
| 6510 | Univer 120mg modified-release capsules (Teva UK Ltd) |
| 6856 | Amlodipine 10mg tablets |
| 7280 | Plendil 10mg modified-release tablets (AstraZeneca UK Ltd) |
| 7398 | Viazem XL 360mg capsules (Thornton & Ross Ltd) |
| 7541 | Nifopress Retard 20mg tablets (Mercury Pharma Group Ltd) |
| 7562 | Cardene 30mg capsules (Astellas Pharma Ltd) |
| 7823 | NIFEDIPINE TAB 5 mg |
| 8024 | DILTIAZEM HCl XL 300 MG CAP |
| 8201 | Nicardipine 30mg capsules |
| 8213 | Nifedipine 24 20mg Modified-release tablet |
| 8257 | Prescal 2.5mg tablets (Novartis Pharmaceuticals UK Ltd) |
| 8310 | Isradipine 2.5mg tablets |
| 8524 | Securon 40mg Tablet (Abbott Laboratories Ltd) |
| 8558 | Adizem xl 120mg Capsule (Napp Pharmaceuticals Ltd) |
| 8759 | Verapamil hcl 120mg modified release tablets |
| 8884 | Cordilox 40mg tablets (IVAX Pharmaceuticals UK Ltd) |
| 8945 | Univer 240mg modified-release capsules (Teva UK Ltd) |
| 8975 | Verapamil 180mg modified-release capsules |
| 9094 | DILTIAZEM HCl SR 300 MG CAP |
| 9211 | ADIZEM-XL 180 MG CAP |
| 9240 | Adizem xl 180mg Capsule (Napp Pharmaceuticals Ltd) |
| 9269 | Nifedipine 40mg modified-release tablets |
| 9334 | Plendil 2.5mg modified-release tablets (AstraZeneca UK Ltd) |
| 9374 | Adizem 60mg Modified-release tablet (Napp Pharmaceuticals Ltd) |
| 9386 | Nicardipine 45mg modified-release capsules |
| 9410 | Angitil SR 120 capsules (Chiesi Ltd) |
| 9437 | Plendil 5mg modified-release tablets (AstraZeneca UK Ltd) |
| 9485 | Hypolar Retard 20 tablets (Sandoz Ltd) |
| 9553 | Slofedipine XL 60 tablets (Zentiva) |
| 9569 | Verapamil 120mg modified-release tablets |
| 9573 | Slofedipine XL 30mg tablets (Zentiva) |
| 9670 | Motens 4mg tablets (GlaxoSmithKline UK Ltd) |
| 9708 | Diltiazem 24hr 120mg modified-release capsules |
| 9723 | Calcicard CR 90mg tablets (Teva UK Ltd) |
| 9750 | Nifedipine 60mg modified-release capsules |
| 10136 | Nifedipress MR 20 tablets (Dexcel-Pharma Ltd) |
| 10153 | Felendil xl 5mg Modified-release tablet (Ratiopharm UK Ltd) |
| 10246 | Adipine XL 60mg tablets (Chiesi Ltd) |
| 10267 | Adizem-XL 200mg capsules (Napp Pharmaceuticals Ltd) |
| 10595 | Nimotop 30mg tablets (Bayer Plc) |
| 10688 | Verapamil 160mg tablets |
| 10832 | Securon 80mg Tablet (Abbott Laboratories Ltd) |
| 11223 | Angitil SR 90 capsules (Chiesi Ltd) |
| 11512 | Nifedipress MR 10 tablets (Dexcel-Pharma Ltd) |
| 11547 | Nimodipine 30mg tablets |
| 11567 | Ramipril 5mg with felodipine 5mg modified-release tablet |
| 11769 | Calchan MR 20 tablets (Ranbaxy (UK) Ltd) |
| 11770 | Dilzem SR 60 capsules (Teva UK Ltd) |
| 11777 | Verapamil 40mg/5ml oral solution sugar free |
| 11922 | Diltiazem 60mg/5ml oral suspension |
| 11943 | Cardene 20mg capsules (Astellas Pharma Ltd) |
| 11965 | Ramipril 2.5mg with felodipine 2.5mg modified-release tablet |
| 11966 | Motens 2mg tablets (GlaxoSmithKline UK Ltd) |
| 11972 | Vertab SR 240 tablets (Chiesi Ltd) |
| 11973 | Calcicard CR 120mg tablets (Teva UK Ltd) |
| 12104 | Cordilox 160mg tablets (IVAX Pharmaceuticals UK Ltd) |
| 12392 | Univer 180mg modified-release capsules (Teva UK Ltd) |
| 12606 | Nifelease 20mg Modified-release tablet (Eastern Pharmaceuticals Ltd) |
| 12613 | Unipine xl 30mg Modified-release tablet (Genus Pharmaceuticals Ltd) |
| 12705 | Angiozem CR 90mg tablets (Ashbourne Pharmaceuticals Ltd) |
| 12875 | Cardene SR 45mg capsules (Astellas Pharma Ltd) |
| 13027 | Viazem XL 240mg capsules (Thornton & Ross Ltd) |
| 13033 | Angitil XL 240 capsules (Chiesi Ltd) |
| 13075 | Dilzem XL 180 capsules (Teva UK Ltd) |
| 13127 | Dilzem XL 240 capsules (Teva UK Ltd) |
| 13139 | Adipine XL 30mg tablets (Chiesi Ltd) |
| 13240 | Dilzem XL 120 capsules (Teva UK Ltd) |
| 13243 | Lercanidipine 20mg tablets |
| 13251 | Vera-Til SR 240mg tablets (Tillomed Laboratories Ltd) |
| 13302 | Dilzem SR 90 capsules (Teva UK Ltd) |
| 13410 | Angiozem 60mg modified-release tablets (Ashbourne Pharmaceuticals Ltd) |
| 13672 | Angiopine MR 10mg tablets (Ashbourne Pharmaceuticals Ltd) |
| 13699 | Angiopine la 40mg Tablet (Ashbourne Pharmaceuticals Ltd) |
| 13856 | Verapress MR 240mg tablets (Actavis UK Ltd) |
| 13926 | Diltiazem 360mg modified-release capsules |
| 13965 | Cordilox MR 240mg tablets (Teva UK Ltd) |
| 14300 | Zanidip 20mg tablets (Recordati Pharmaceuticals Ltd) |
| 14305 | Vascalpha 10mg modified-release tablets (Actavis UK Ltd) |
| 14861 | Calchan MR 10 tablets (Ranbaxy (UK) Ltd) |
| 15288 | Angitil XL 300 capsules (Chiesi Ltd) |
| 15652 | Mibefradil 50mg Tablet |
| 15659 | DILTIAZEM HCL S/R 180 CAP |
| 16038 | Dilzem SR 120 capsules (Teva UK Ltd) |
| 16073 | Nifedipress MR 10 tablets (Teva UK Ltd) |
| 16162 | Amlodipine 5mg/5ml oral suspension |
| 16328 | Verapress MR 240mg tablets (Dexcel-Pharma Ltd) |
| 16677 | Cordilox 80mg tablets (IVAX Pharmaceuticals UK Ltd) |
| 16850 | Angiozem CR 120mg tablets (Ashbourne Pharmaceuticals Ltd) |
| 17006 | Triapin 5mg/5mg modified-release tablets (Sanofi) |
| 17325 | Cardilate MR 10mg tablets (Teva UK Ltd) |
| 17406 | Zemtard 180 XL capsules (Galen Ltd) |
| 17425 | Zemtard 120 XL capsules (Galen Ltd) |
| 17448 | Nifedipress mr 10mg Modified-release tablet (Sterwin Medicines) |
| 17474 | Felodipine 5mg modified-release / Ramipril 5mg tablets |
| 17492 | Zemtard 300 XL capsules (Galen Ltd) |
| 17557 | Felotens XL 5mg tablets (Thornton & Ross Ltd) |
| 17566 | Felotens XL 10mg tablets (Thornton & Ross Ltd) |
| 17586 | Slozem 300mg capsules (Merck Serono Ltd) |
| 17599 | Verapress MR 240mg tablets (Sandoz Ltd) |
| 17640 | Amlostin 5mg tablets (Discovery Pharmaceuticals Ltd) |
| 17666 | Viazem XL 180mg capsules (Thornton & Ross Ltd) |
| 18038 | Nisoldipine 20mg modified-release tablets |
| 18223 | Trandolapril with verapamil 2mg + 180mg Modified-release capsule |
| 18379 | Dilcardia SR 90mg capsules (Generics (UK) Ltd) |
| 18403 | Diltiazem HCl 180mg Modified-release capsule (Hillcross Pharmaceuticals Ltd) |
| 18404 | Diltiazem 60mg modified-release capsules (A A H Pharmaceuticals Ltd) |
| 18690 | SECURON (CALENDAR PACK) 120 MG TAB |
| 18830 | Disogram SR 90mg capsules (Ranbaxy (UK) Ltd) |
| 18834 | Disogram SR 60mg capsules (Ranbaxy (UK) Ltd) |
| 18852 | Disogram SR 120mg capsules (Ranbaxy (UK) Ltd) |
| 18874 | Disogram SR 180mg capsules (Ranbaxy (UK) Ltd) |
| 19015 | ADIZEM CONTINUS 120 MG TAB |
| 19129 | Syscor MR 10 tablets (Forest Laboratories UK Ltd) |
| 19170 | Tensipine MR 10 tablets (Thornton & Ross Ltd) |
| 19175 | Verapamil 40mg tablets (IVAX Pharmaceuticals UK Ltd) |
| 19325 | Cordilox 2.5mg/ml Injection (IVAX Pharmaceuticals UK Ltd) |
| 19426 | Disogram SR 240mg capsules (Ranbaxy (UK) Ltd) |
| 19440 | Disogram SR 300mg capsules (Ranbaxy (UK) Ltd) |
| 19690 | Verapamil 180mg modified-release / Trandolapril 2mg capsules |
| 20257 | Cardilate MR 20mg tablets (IVAX Pharmaceuticals UK Ltd) |
| 20311 | Nifedipress mr 20mg Modified-release tablet (Generics (UK) Ltd) |
| 20459 | Felendil xl 10mg Modified-release tablet (Ratiopharm UK Ltd) |
| 20579 | Tarka modified-release capsules (Abbott Laboratories Ltd) |
| 20591 | Nifedipress MR 20 tablets (Teva UK Ltd) |
| 20642 | Bi-carzem sr 60mg Modified-release capsule (Tillomed Laboratories Ltd) |
| 20878 | Angiopine 10 capsules (Ashbourne Pharmaceuticals Ltd) |
| 20890 | Zemtard 240 XL capsules (Galen Ltd) |
| 21145 | Dilcardia SR 60mg capsules (Generics (UK) Ltd) |
| 21162 | Felodipine 2.5mg modified-release / Ramipril 2.5mg tablets |
| 21216 | Hypolar Retard 10mg tablets (Sandoz Ltd) |
| 21665 | CORDILOX |
| 21795 | Retalzem 60 modified-release tablets (Kent Pharmaceuticals Ltd) |
| 21886 | Nifedipress MR 20 tablets (Actavis UK Ltd) |
| 21918 | Optil 60mg modified-release tablets (Opus Pharmaceuticals Ltd) |
| 22019 | Calanif 10mg Capsule (Berk Pharmaceuticals Ltd) |
| 22217 | Nimodrel 10mg modified-release tablet (Opus Pharmaceuticals Ltd) |
| 22241 | Mibefradil 100mg Tablet |
| 22619 | Britiazim 60mg Modified-release tablet (Thames Laboratories Ltd) |
| 22826 | Securon 160mg Tablet (Abbott Laboratories Ltd) |
| 23233 | Bi-carzem sr 90mg Modified-release capsule (Tillomed Laboratories Ltd) |
| 23505 | Adizem xl plus 150mg+12.5mg Modified-release capsule (Napp Pharmaceuticals Ltd) |
| 23733 | Optil sr 90mg Modified-release capsule (Opus Pharmaceuticals Ltd) |
| 23736 | Hypolar XL 30 tablets (Sandoz Ltd) |
| 23805 | Nisoldipine 10mg modified-release tablets |
| 23823 | Nisoldipine 30mg modified-release tablets |
| 24228 | Nimodrel 20mg modified-release tablet (Opus Pharmaceuticals Ltd) |
| 24365 | Cardioplen XL 5mg tablets (Chiesi Ltd) |
| 24366 | Cardioplen XL 10mg tablets (Chiesi Ltd) |
| 25132 | Nifopress MR 20mg tablets (Teva UK Ltd) |
| 25572 | Felogen XL 5mg tablets (Generics (UK) Ltd) |
| 25646 | Nivaten retard 20mg Modified-release tablet (Actavis UK Ltd) |
| 25777 | Dilcardia SR 120mg capsules (Generics (UK) Ltd) |
| 25919 | Nifedipine 20mg modified-release tablets (A A H Pharmaceuticals Ltd) |
| 26267 | Optil sr 120mg Modified-release capsule (Opus Pharmaceuticals Ltd) |
| 26269 | Optil sr 180mg Modified-release capsule (Opus Pharmaceuticals Ltd) |
| 26270 | Optil xl 300mg Modified-release capsule (Opus Pharmaceuticals Ltd) |
| 26309 | Optil xl 240mg Modified-release capsule (Opus Pharmaceuticals Ltd) |
| 26774 | Nifedipine 10mg/5ml Oral suspension |
| 27136 | Diltiazem 90mg modified-release tablets (A A H Pharmaceuticals Ltd) |
| 27685 | Diltiazem HCl 300mg Capsule (PLIVA Pharma Ltd) |
| 28438 | Triapin 2.5mg/2.5mg modified-release tablets (Sanofi) |
| 28688 | Nifedipine 10mg modified-release tablets (A A H Pharmaceuticals Ltd) |
| 28721 | Neofel XL 5mg tablets (Kent Pharmaceuticals Ltd) |
| 29044 | Neofel XL 10mg tablets (Kent Pharmaceuticals Ltd) |
| 29145 | Felendil xl 2.5mg Modified-release tablet (Ratiopharm UK Ltd) |
| 30197 | Diltiazem 120mg modified-release capsules |
| 30199 | Nifedipine 30mg modified-release tablets |
| 30242 | Diltiazem 180mg modified-release capsules |
| 30473 | Coroday MR 20mg tablets (Generics (UK) Ltd) |
| 30915 | Cabren 2.5mg modified-release tablets (Teva UK Ltd) |
| 30991 | Cabren 5mg modified-release tablets (Teva UK Ltd) |
| 31337 | Syscor MR 20 tablets (Forest Laboratories UK Ltd) |
| 31761 | Amlostin 10mg tablets (Discovery Pharmaceuticals Ltd) |
| 32089 | Diltiazem HCl 120mg Modified-release capsule (Hillcross Pharmaceuticals Ltd) |
| 33025 | Nimodrel XL 30mg tablets (Zurich Pharmaceuticals) |
| 33932 | Parmid XL 5mg tablets (Sandoz Ltd) |
| 34093 | Amlodipine 10mg tablets (A A H Pharmaceuticals Ltd) |
| 34101 | Nifedipine mr 20mg Modified-release tablet (IVAX Pharmaceuticals UK Ltd) |
| 34115 | Nifedipine 60mg Modified-release tablet |
| 34146 | Nifedipine mr 10mg Modified-release tablet (IVAX Pharmaceuticals UK Ltd) |
| 34187 | Nifedipine 10mg Modified-release tablet (Generics (UK) Ltd) |
| 34377 | Diltiazem HCl 90mg Modified-release capsule (Hillcross Pharmaceuticals Ltd) |
| 34581 | Diltiazem HCl 60mg Modified-release tablet (Kent Pharmaceuticals Ltd) |
| 34824 | Diltiazem HCl 120mg Modified-release tablet (IVAX Pharmaceuticals UK Ltd) |
| 35084 | Vascalpha 5mg modified-release tablets (Actavis UK Ltd) |
| 35096 | Exforge 10mg/160mg tablets (Novartis Pharmaceuticals UK Ltd) |
| 35173 | Valsartan 160mg with amlodipine 5mg tablets |
| 35174 | Valsartan 80mg with amlodipine 5mg tablets |
| 35189 | Amlodipine 10mg / Valsartan 160mg tablets |
| 35304 | Valsartan 160mg with amlodipine 10mg tablets |
| 35317 | Exforge 5mg/80mg tablets (Novartis Pharmaceuticals UK Ltd) |
| 35329 | Amlodipine 5mg / Valsartan 80mg tablets |
| 35343 | Amlodipine 5mg / Valsartan 160mg tablets |
| 35592 | Cardioplen XL 2.5mg tablets (Chiesi Ltd) |
| 35697 | Exforge 5mg/160mg tablets (Novartis Pharmaceuticals UK Ltd) |
| 37025 | Nifedipine 20mg modified-release tablets |
| 37184 | Valni XL 30mg tablets (Zentiva) |
| 37774 | Kenzem SR 60mg capsules (Kent Pharmaceuticals Ltd) |
| 37897 | Felotens XL 2.5mg tablets (Thornton & Ross Ltd) |
| 38545 | Tildiem LA 200 capsules (Sanofi) |
| 38632 | Adizem-SR 90mg capsules (Napp Pharmaceuticals Ltd) |
| 38634 | Adizem-XL 300mg capsules (Napp Pharmaceuticals Ltd) |
| 38818 | Adizem-SR 120mg capsules (Napp Pharmaceuticals Ltd) |
| 38831 | Adizem-SR 180mg capsules (Napp Pharmaceuticals Ltd) |
| 38855 | Adizem-XL 180mg capsules (Napp Pharmaceuticals Ltd) |
| 38865 | Adizem-XL 120mg capsules (Napp Pharmaceuticals Ltd) |
| 38876 | Tildiem LA 300 capsules (Sanofi) |
| 38882 | Adizem-XL 240mg capsules (Napp Pharmaceuticals Ltd) |
| 38964 | Adizem-SR 120mg tablets (Napp Pharmaceuticals Ltd) |
| 39009 | Verapamil 40mg tablets (Teva UK Ltd) |
| 39171 | Bi-Carzem SR 60mg capsules (Tillomed Laboratories Ltd) |
| 39800 | Valni XL 60mg tablets (Zentiva) |
| 39914 | Amlodipine 5mg tablets (Teva UK Ltd) |
| 39984 | Sevikar 20mg/5mg tablets (Daiichi Sankyo UK Ltd) |
| 40074 | Nifedipine 20mg Capsule |
| 40316 | Olmesartan medoxomil 20mg / Amlodipine 5mg tablets |
| 40405 | Verapamil 120mg tablets (Teva UK Ltd) |
| 40633 | Vascalpha 5mg modified-release tablets (Almus Pharmaceuticals Ltd) |
| 40639 | Olmesartan medoxomil 40mg / Amlodipine 5mg tablets |
| 40668 | Olmesartan medoxomil 40mg / Amlodipine 10mg tablets |
| 41203 | Sevikar 40mg/10mg tablets (Daiichi Sankyo UK Ltd) |
| 41205 | Sevikar 40mg/5mg tablets (Daiichi Sankyo UK Ltd) |
| 41489 | Bi-Carzem SR 120mg capsules (Tillomed Laboratories Ltd) |
| 42210 | Amlodipine 10mg tablets (Zentiva) |
| 42819 | Diltiazem xl 240mg Capsule (Hillcross Pharmaceuticals Ltd) |
| 42912 | Nifedipine 10mg capsules (Teva UK Ltd) |
| 43410 | Nifedipine extra 60mg Modified-release tablet |
| 43430 | Diltiazem 120mg modified-release tablets (A A H Pharmaceuticals Ltd) |
| 43753 | Adalat LA 30 tablets (Bayer Plc) |
| 43818 | Adalat LA 60 tablets (Bayer Plc) |
| 44192 | Zemret 240 XL capsules (Tillomed Laboratories Ltd) |
| 45051 | Verapamil hc 240mg Modified-release tablet (Actavis UK Ltd) |
| 45685 | Adanif XL 30mg tablets (Focus Pharmaceuticals Ltd) |
| 46355 | Sevikar HCT 20mg/5mg/12.5mg tablets (Daiichi Sankyo UK Ltd) |
| 46715 | Olmesartan medoxomil with amlodipine and hydrochlorothiazide 40mg + 10mg + 12.5mg Tablet |
| 46724 | Amlodipine 5mg/5ml oral solution |
| 46884 | Verapamil hc 240mg Modified-release tablet (Sandoz Ltd) |
| 47027 | Nifedipine 10mg Modified-release tablet (Kent Pharmaceuticals Ltd) |
| 47230 | Verapamil 240mg modified-release tablets (Teva UK Ltd) |
| 47331 | Lercanidipine 10mg tablets (Generics (UK) Ltd) |
| 47415 | Diltiazem sr 60mg Capsule (Hillcross Pharmaceuticals Ltd) |
| 47529 | Nifedipine 20mg/ml oral drops |
| 47573 | Sevikar HCT 40mg/5mg/12.5mg tablets (Daiichi Sankyo UK Ltd) |
| 47608 | Zemret 300 XL capsules (Tillomed Laboratories Ltd) |
| 47727 | Sevikar HCT 40mg/5mg/25mg tablets (Daiichi Sankyo UK Ltd) |
| 48272 | Diltiazem 60mg modified-release capsules (Alliance Healthcare (Distribution) Ltd) |
| 48282 | Diltiazem 90mg modified-release capsules (A A H Pharmaceuticals Ltd) |
| 48288 | Diltiazem 120mg modified-release capsules (A A H Pharmaceuticals Ltd) |
| 48457 | Diltiazem 90mg modified-release capsules (Alliance Healthcare (Distribution) Ltd) |
| 49001 | Diltiazem 120mg modified-release tablets (Alliance Healthcare (Distribution) Ltd) |
| 49289 | Diltiazem 120mg modified-release capsules (Alliance Healthcare (Distribution) Ltd) |
| 49338 | Nifedipine 20mg modified-release tablets (Alliance Healthcare (Distribution) Ltd) |
| 49390 | Diltiazem 90mg modified-release tablets (Alliance Healthcare (Distribution) Ltd) |
| 49762 | Nifedipine 10mg modified-release tablets (Alliance Healthcare (Distribution) Ltd) |
| 53220 | Sevikar HCT 40mg/10mg/25mg tablets (Daiichi Sankyo UK Ltd) |
| 54633 | Amlodipine 5mg tablets (Bristol Laboratories Ltd) |
| 55306 | Folpik XL 5mg tablets (Teva UK Ltd) |
| 55358 | Olmesartan medoxomil with amlodipine and hydrochlorothiazide 40mg + 10mg + 25mg Tablet |
| 56467 | Tildiem 60mg modified-release tablets (DE Pharmaceuticals) |
| 56469 | Adalat LA 60 tablets (Necessity Supplies Ltd) |
| 57859 | Diltiazem 90mg modified-release tablets (Cubic Pharmaceuticals Ltd) |
| 58339 | Neofel XL 2.5mg tablets (Almus Pharmaceuticals Ltd) |
| 58990 | Nifedipine 10mg modified-release tablets (Cubic Pharmaceuticals Ltd) |
| 2 | Bendroflumethiazide 2.5mg tablets |
| 6 | Furosemide 40mg tablets |
| 55 | Furosemide 20mg tablets |
| 56 | Co-amilofruse 5mg/40mg tablets |
| 58 | Bendroflumethiazide 5mg tablets |
| 193 | Co-amilofruse 2.5mg/20mg tablets |
| 211 | Frumil 40mg+5mg Tablet (Helios Healthcare Ltd) |
| 348 | Moduretic Tablet (Bristol-Myers Squibb Pharmaceuticals Ltd) |
| 542 | Hydrochlorothiazide 25mg tablets |
| 562 | Furosemide 10mg/ml Injection |
| 605 | Chlortalidone 50mg tablets |
| 692 | Spironolactone 25mg tablets |
| 708 | Spironolactone 50mg tablets |
| 787 | Spironolactone 100mg capsule |
| 814 | Bumetanide 1mg tablets |
| 923 | Co-amilozide 5mg/50mg tablets |
| 924 | Co-amilozide 2.5mg/25mg tablets |
| 1060 | Amiloride 5mg tablets |
| 1125 | Navidrex -k Tablet (Novartis Pharmaceuticals UK Ltd) |
| 1170 | Cyclopenthiazide 500microgram tablets |
| 1209 | Neo-Naclex 5mg tablets (Mercury Pharma Group Ltd) |
| 1211 | Bendroflumethiazide 2.5mg / Potassium chloride 630mg (potassium 8.4mmol) modified-release tablets |
| 1213 | Neo-Naclex-K modified-release tablets (Mercury Pharma Group Ltd) |
| 1251 | Moduret 25 tablets (Merck Sharp & Dohme Ltd) |
| 1297 | Aldactide 50 tablets (Pfizer Ltd) |
| 1301 | Frumil ls 20mg+2.5mg Tablet (Helios Healthcare Ltd) |
| 1369 | Furosemide with amiloride 40mg+5mg Tablet |
| 1721 | Dyazide 50mg/25mg tablets (Mercury Pharma Group Ltd) |
| 1776 | Burinex K modified-release tablets (LEO Pharma) |
| 2001 | Aldactide 25 tablets (Pfizer Ltd) |
| 2002 | Amiloride 5mg / hydrochlorothiazide 50mg tablets |
| 2046 | Navidrex 500microgram tablets (Mercury Pharma Group Ltd) |
| 2142 | Spironolactone 100mg tablets |
| 2179 | Triamterene 50mg capsules |
| 2255 | Navispare 2.5mg/250microgram tablets (Mercury Pharma Group Ltd) |
| 2284 | Diamox sr 250mg Capsule (Wyeth Pharmaceuticals) |
| 2389 | Aldactone 25mg tablets (Pfizer Ltd) |
| 2493 | Burinex A 5mg/1mg tablets (LEO Pharma) |
| 2495 | Bumetanide with Amiloride tablets |
| 2551 | Acetazolamide 250mg tablets |
| 2612 | Indapamide 2.5mg tablets |
| 2670 | AMILCO TAB |
| 2772 | Lasoride 5mg/40mg tablets (Sanofi) |
| 2788 | Burinex 1mg tablets (LEO Pharma) |
| 2833 | CYCLOPENTHIAZIDE -K tablets |
| 2961 | Frusene 50mg/40mg tablets (Orion Pharma (UK) Ltd) |
| 2979 | Centyl k Tablet (Edwin Burgess Ltd) |
| 3050 | Furosemide with triamterene 40mgwith50mg Tablet |
| 3054 | Hygroton 100mg Tablet (Alliance Pharmaceuticals Ltd) |
| 3056 | Natrilix SR 1.5mg tablets (Servier Laboratories Ltd) |
| 3248 | Furosemide 500mg tablets |
| 3263 | Acetazolamide 250mg modified-release capsules |
| 3265 | Diamox 250mg Tablet (Wyeth Pharmaceuticals) |
| 3293 | Moduretic Oral solution (Bristol-Myers Squibb Pharmaceuticals Ltd) |
| 3517 | Hydrochlorothiazide 50mg tablets |
| 3548 | Chlortalidone 100mg tablets |
| 3701 | Amiloride 2.5mg / hydrochlorothiazide 25mg tablets |
| 3793 | Co-amilofruse 10mg/80mg tablets |
| 3876 | BENDROFLUAZIDE 10 MG TAB |
| 3902 | FRUSEMIDE 20MG/SPIRONOLACTONE 50MG MG CAP |
| 3962 | TRIAMTERENE 50MG HYDROCHLOROTHIAZIDE25MG TAB |
| 3997 | Hygroton 50mg tablets (Alliance Pharmaceuticals Ltd) |
| 4034 | Amiloride 5mg / hydrochlorothiazide 50mg/5ml solution |
| 4044 | Diurexan 20mg tablets (Meda Pharmaceuticals Ltd) |
| 4068 | Dytac 50mg capsules (Mercury Pharma Group Ltd) |
| 4211 | Furosemide with amiloride 20mg+2.5mg Tablet |
| 4258 | Lasix 20mg/2ml solution for injection ampoules (Sanofi) |
| 4332 | Metolazone 5mg tablets |
| 4334 | Metolazone 500microgram low dose Tablet |
| 4661 | Spironolactone 50mg / Furosemide 20mg capsules |
| 4705 | Furosemide 20mg/2ml Injection |
| 4873 | Fru-Co 5mg/40mg tablets (Teva UK Ltd) |
| 4960 | Aldactone 50mg tablets (Pfizer Ltd) |
| 5112 | Indapamide 1.5mg modified-release tablets |
| 5218 | Bumetanide 1mg/5ml oral solution sugar free |
| 5220 | Furosemide with amiloride 80mg+10mg Tablet |
| 5249 | Furosemide 50mg/5ml oral solution sugar free |
| 5416 | Co-triamterzide 50mg/25mg tablets |
| 5727 | Amiloride 2.5mg / Cyclopenthiazide 250microgram tablets |
| 5728 | Furosemide 40mg/5ml oral solution sugar free |
| 5868 | Frusol 20mg/5ml oral solution (Rosemont Pharmaceuticals Ltd) |
| 5893 | Diamox 250mg tablets (Mercury Pharma Group Ltd) |
| 5954 | Diamox SR 250mg capsules (Mercury Pharma Group Ltd) |
| 6118 | Furosemide 20mg/5ml oral solution sugar free |
| 6160 | Bumetanide 500microgram / Potassium chloride 573mg (potassium 7.7mmol) modified-release tablets |
| 6815 | Spironolactone 50mg/5ml oral suspension sugar free |
| 7136 | Dytide capsules (Mercury Pharma Group Ltd) |
| 7351 | Bendroflumethiazide 2.5mg/5ml oral suspension |
| 7441 | Lasilactone 20mg/50mg capsules (Sanofi) |
| 7582 | Lasikal modified-release tablets (Borg Medicare) |
| 7606 | Lasix 40mg tablets (Sanofi) |
| 7618 | Xipamide 20mg tablets |
| 7625 | FRUSEMIDE 20MG/POTASSIUM 10MMOL S/R MG TAB |
| 7641 | Natrilix 2.5mg tablets (Servier Laboratories Ltd) |
| 7698 | Aprinox 5mg tablets (Amdipharm Plc) |
| 7709 | Arelix 6mg Capsule (Hoechst Marion Roussel) |
| 7734 | Diumide-K Continus tablets (Teofarma) |
| 7740 | Triamterene 50mg / Benzthiazide 25mg capsules |
| 7799 | Lasix 20mg tablets (Borg Medicare) |
| 7806 | Bumetanide 5mg tablets |
| 7888 | FRUSEMIDE 40MG/8MMOL POTASSIUM S/R MG TAB |
| 7952 | Aldactone 100mg tablets (Pfizer Ltd) |
| 7961 | Spironolactone 50mg with hydroflumethiazide 50mg tablet |
| 7991 | Spiroctan 100mg Capsule (Roche Products Ltd) |
| 8052 | Torasemide 5mg tablets |
| 8058 | Normetic Tablet (Abbott Laboratories Ltd) |
| 8102 | Furosemide 40mg / Potassium chloride 600mg (potassium 8mmol) modified-release tablets |
| 8303 | Tenavoid Tablet (Edwin Burgess Ltd) |
| 8521 | Spironolactone 25mg with hydroflumethiazide 25mg tablet |
| 8526 | Aprinox 2.5mg tablets (Amdipharm Plc) |
| 8602 | Metenix 5mg tablets (Sanofi) |
| 8817 | Diamox sustets 500 500mg Sustets (Wyeth Pharmaceuticals) |
| 8836 | Chlorothiazide 500mg tablets |
| 8891 | Hygroton -k Tablet (Novartis Pharmaceuticals UK Ltd) |
| 8897 | Triam-Co 50mg/25mg tablets (IVAX Pharmaceuticals UK Ltd) |
| 9223 | Triamterene with hydrochlorothiazide 50mg + 25mg Tablet |
| 9456 | Amiloride 5mg / furosemide 40mg tablets |
| 9680 | Frusol 40mg/5ml oral solution (Rosemont Pharmaceuticals Ltd) |
| 9935 | Amiloride 5mg/5ml oral solution sugar free |
| 10066 | Torem 5mg tablets (Meda Pharmaceuticals Ltd) |
| 10214 | Spironolactone 5mg/5ml oral suspension sugar free |
| 10251 | Eplerenone 25mg tablets |
| 10392 | Lasix 500mg tablets (Sanofi) |
| 10422 | Lasix 50mg/5ml Injection (Hoechst UK Ltd) |
| 10781 | Lasix with k Tablet (Hoechst Marion Roussel) |
| 10796 | CHLORTHALIDONE 25MG/POTASSIUM6.7MMOL S/R MG TAB |
| 11156 | Spirolone 25mg Tablet (Berk Pharmaceuticals Ltd) |
| 11265 | Triamterene 50mg / Furosemide 40mg tablets |
| 11268 | Torem 2.5mg tablets (Meda Pharmaceuticals Ltd) |
| 11384 | Co-flumactone 50mg/50mg tablets |
| 11487 | Torasemide 2.5mg tablets |
| 11519 | Spironolactone 25mg/5ml oral suspension sugar free |
| 12226 | Burinex 1mg/5ml Oral solution (LEO Pharma) |
| 12294 | Burinex 5mg tablets (LEO Pharma) |
| 12354 | Etacrynic 50mg tablets |
| 12360 | Nephril 1mg Tablet (Pfizer Ltd) |
| 12367 | Piretanide 6mg capsule |
| 12440 | Hydrosaluric 25mg tablets (Merck Sharp & Dohme Ltd) |
| 12546 | Kalspare Tablet (Dominion Pharma) |
| 12547 | Triamterene 50mg / Chlortalidone 50mg tablets |
| 12926 | Polythiazide 1mg tablets |
| 12946 | Spironolactone 10mg/5ml oral suspension sugar free |
| 13264 | Spironolactone 15mg/5ml oral suspension |
| 13352 | Midamor 5mg Tablet (MSD Thomas Morson Pharmaceuticals) |
| 13363 | Esidrex 50mg Tablet (Novartis Pharmaceuticals UK Ltd) |
| 13435 | Frumil Forte 10mg/80mg tablets (Sanofi) |
| 13525 | Hydrenox 50mg Tablet (Knoll Ltd) |
| 14109 | Spironolactone 100mg/5ml oral solution sugar free |
| 14144 | Inspra 25mg tablets (Pfizer Ltd) |
| 14587 | Amiloride 5mg / Bumetanide 1mg tablets |
| 14761 | Frusid 40mg tablets (Dr Reddy's Laboratories (UK) Ltd) |
| 14837 | Frusol 50mg/5ml oral solution (Rosemont Pharmaceuticals Ltd) |
| 15052 | Spiroctan 50mg Tablet (Roche Products Ltd) |
| 15127 | Hydrochlorothiazide with triamterene 25mgwith50mg Tablet |
| 15341 | Burinex 0.5mg/ml Injection (LEO Pharma) |
| 15347 | Acetazolamide 500mg powder for solution for injection vials |
| 15457 | Baycaron 25mg Tablet (Bayer Plc) |
| 15602 | NATRILIX 5 MG TAB |
| 15811 | Co-flumactone 25mg/25mg tablets |
| 15874 | Amiloride 2.5mg / furosemide 20mg tablets |
| 16206 | Froop 40mg tablets (Ashbourne Pharmaceuticals Ltd) |
| 16498 | Kalspare tablets (DHP Healthcare Ltd) |
| 16531 | Eplerenone 50mg tablets |
| 17143 | Mefruside 25mg Tablet |
| 17252 | Esidrex 25mg Tablet (Novartis Pharmaceuticals UK Ltd) |
| 17273 | BENDROFLUAZIDE K 2.5 MG TAB |
| 17561 | Bendroflumethiazide 2.5mg / Potassium chloride 573mg (potassium 7.7mmol) modified-release tablets |
| 17720 | Saluric 500mg Tablet (Merck Sharp & Dohme Ltd) |
| 17776 | BENDROFLUAZIDE 40 MG TAB |
| 17902 | Spirolone 100mg Tablet (Berk Pharmaceuticals Ltd) |
| 17960 | Furosemide 20mg / Potassium chloride 750mg (potassium 10mmol) modified-release tablets |
| 18096 | Torasemide 10mg tablets |
| 18259 | BENDROFLUAZIDE K TAB |
| 18332 | Aridil 20mg+2.5mg Tablet (C P Pharmaceuticals Ltd) |
| 18361 | Amilmaxco 5mg/50mg tablets (Ashbourne Pharmaceuticals Ltd) |
| 18497 | Amiloride 10mg / furosemide 80mg tablets |
| 18726 | Triamaxco 50mg/25mg tablets (Ashbourne Pharmaceuticals Ltd) |
| 18733 | Co-amilozide 5mg with 50mg/ml oral solution |
| 18973 | Centyl 2.5mg Tablet (Edwin Burgess Ltd) |
| 19192 | Furosemide 40mg Tablet (M & A Pharmachem Ltd) |
| 19194 | Furosemide 20mg tablets (Teva UK Ltd) |
| 19258 | Furosemide 50mg/5ml solution for injection ampoules |
| 19300 | Bumetanide 2mg/4ml solution for injection ampoules |
| 19352 | Xuret 0.5mg Tablet (Galen Ltd) |
| 19890 | Hydrochlorothiazide with amiloride 25mgwith2.5mg Tablet |
| 20066 | Amil-Co 5mg/50mg tablets (IVAX Pharmaceuticals UK Ltd) |
| 20426 | Centyl k 2.5mg+7.7mmol Tablet (Edwin Burgess Ltd) |
| 20431 | Centyl K modified-release tablets (LEO Pharma) |
| 20538 | Frumax 40mg Tablet (Ashbourne Pharmaceuticals Ltd) |
| 21803 | Berkozide 2.5mg Tablet (Berk Pharmaceuticals Ltd) |
| 21848 | AMILOSPARE 5 MG TAB |
| 21849 | Dryptal 40mg Tablet (Berk Pharmaceuticals Ltd) |
| 21867 | Berkozide 5mg Tablet (Berk Pharmaceuticals Ltd) |
| 21938 | Froop Co 5mg/40mg tablets (Ashbourne Pharmaceuticals Ltd) |
| 22242 | NAVIDREX |
| 22658 | Torem 10mg tablets (Meda Pharmaceuticals Ltd) |
| 22923 | Hydrochlorothiazide with amiloride 50mg with 5mg Tablet |
| 23091 | Spirospare 100 tablets (Ashbourne Pharmaceuticals Ltd) |
| 23427 | Bendroflumethiazide 5mg tablets (A A H Pharmaceuticals Ltd) |
| 24008 | Vasetic Tablet (Shire Pharmaceuticals Ltd) |
| 24189 | Neo-bendromax 2.5mg Tablet (Ashbourne Pharmaceuticals Ltd) |
| 24190 | Neo-bendromax 5mg Tablet (Ashbourne Pharmaceuticals Ltd) |
| 25717 | Furosemide 40mg tablets (Generics (UK) Ltd) |
| 25965 | Co-amilofruse 2.5mg/20mg tablets (Wockhardt UK Ltd) |
| 26217 | Berkamil 5mg Tablet (Berk Pharmaceuticals Ltd) |
| 26220 | Delvas Tablet (Berk Pharmaceuticals Ltd) |
| 26256 | Opumide 2.5mg Tablet (Opus Pharmaceuticals Ltd) |
| 26275 | Nindaxa 2.5 tablets (Ashbourne Pharmaceuticals Ltd) |
| 27256 | Bendroflumethiazide 2.5mg tablets (Wockhardt UK Ltd) |
| 27447 | Furosemide 40mg tablets (Wockhardt UK Ltd) |
| 27689 | Bendroflumethiazide 2.5mg tablets (IVAX Pharmaceuticals UK Ltd) |
| 27690 | Furosemide 40mg tablets (A A H Pharmaceuticals Ltd) |
| 27957 | Natramid 2.5mg Tablet (Trinity Pharmaceuticals Ltd) |
| 29497 | Acetazolamide 125mg/5ml oral suspension |
| 29694 | Inspra 50mg tablets (Pfizer Ltd) |
| 29780 | Furosemide 20mg Tablet (C P Pharmaceuticals Ltd) |
| 29991 | Centyl 5mg Tablet (Edwin Burgess Ltd) |
| 30625 | Furosemide 20mg tablets (A A H Pharmaceuticals Ltd) |
| 30773 | Co-amilofruse 5mg+40mg Tablet (Berk Pharmaceuticals Ltd) |
| 30875 | Furosemide 250mg/25ml solution for injection ampoules |
| 31219 | Spironolactone 100mg tablets (A A H Pharmaceuticals Ltd) |
| 31375 | Amilamont 5mg/5ml oral solution sugar free (Rosemont Pharmaceuticals Ltd) |
| 31529 | Spironolactone 25mg tablets (Teva UK Ltd) |
| 31548 | Furosemide 20mg tablets (Actavis UK Ltd) |
| 31670 | Bendroflumethiazide 2.5mg tablets (Teva UK Ltd) |
| 31820 | Bendroflumethiazide 5mg tablets (Wockhardt UK Ltd) |
| 31932 | Bumetanide 1mg tablets (C P Pharmaceuticals Ltd) |
| 32091 | Bumetanide 1mg tablets (A A H Pharmaceuticals Ltd) |
| 32277 | Furosemide 80mg/8ml solution for injection pre-filled syringes |
| 33083 | Indapamide 2.5mg tablets (Teva UK Ltd) |
| 33415 | Bendroflumethiazide 2.5mg tablets (Generics (UK) Ltd) |
| 33651 | Bendroflumethiazide 2.5mg tablets (A A H Pharmaceuticals Ltd) |
| 33837 | Amiloride 5mg tablets (A A H Pharmaceuticals Ltd) |
| 34006 | Furosemide 40mg tablets (Actavis UK Ltd) |
| 34059 | Bendroflumethiazide 2.5mg tablets (Actavis UK Ltd) |
| 34124 | Bendroflumethiazide 5mg tablets (Actavis UK Ltd) |
| 34324 | Amiloride 5mg tablets (Teva UK Ltd) |
| 34367 | Co-amilozide 2.5mg/25mg tablets (Wockhardt UK Ltd) |
| 34374 | Furosemide 40mg tablets (Teva UK Ltd) |
| 34557 | Furosemide 40mg tablets (IVAX Pharmaceuticals UK Ltd) |
| 34750 | Amiloride 5mg tablets (Actavis UK Ltd) |
| 35162 | Furosemide 20mg/2ml solution for injection ampoules |
| 35399 | Acetazolamide 250mg/5ml oral suspension |
| 35789 | Spironolactone 25mg Tablet (Celltech Pharma Europe Ltd) |
| 36190 | Furosemide 5mg/5ml oral solution sugar free |
| 36767 | Bumetanide 1mg tablets (IVAX Pharmaceuticals UK Ltd) |
| 37294 | Triamterene with chlortalidone 50mg + 25mg Tablet |
| 38901 | Frumil LS 20mg/2.5mg tablets (Sanofi) |
| 39447 | Varbim XL 1.5mg tablets (Teva UK Ltd) |
| 39807 | Frumil 40mg/5mg tablets (Sanofi) |
| 40149 | Bendroflumethiazide 5mg tablets (IVAX Pharmaceuticals UK Ltd) |
| 40886 | Bendroflumethiazide 2.5mg tablets (Almus Pharmaceuticals Ltd) |
| 41517 | Bendroflumethiazide 5mg tablets (Teva UK Ltd) |
| 41556 | Co-amilozide 5mg/50mg tablets (Teva UK Ltd) |
| 41592 | Spironolactone 100mg tablets (Actavis UK Ltd) |
| 41630 | Amiloride 5mg Tablet (IVAX Pharmaceuticals UK Ltd) |
| 41660 | Spironolactone 100mg tablets (Teva UK Ltd) |
| 41706 | Spironolactone 50mg tablets (IVAX Pharmaceuticals UK Ltd) |
| 41719 | Co-amilofruse 5mg/40mg tablets (Actavis UK Ltd) |
| 41861 | Tensaid XL 1.5mg tablets (Generics (UK) Ltd) |
| 42142 | Moduretic 5mg/50mg tablets (Merck Sharp & Dohme Ltd) |
| 42906 | Indapamide 2.5mg tablets (Niche Generics Ltd) |
| 44168 | Indipam XL 1.5mg tablets (Actavis UK Ltd) |
| 46302 | Neo-Naclex 2.5mg tablets (Mercury Pharma Group Ltd) |
| 46916 | Co-amilozide 5mg/50mg tablets (A A H Pharmaceuticals Ltd) |
| 47018 | Spironolactone 25mg/5ml oral suspension |
| 47804 | Co-triamterzide 50mg/25mg tablets (A A H Pharmaceuticals Ltd) |
| 53674 | Metolazone 2.5mg tablets |

Disease-modifying anti-rheumatic drugs (DMARDs) – product codes

| **prodcode** | **Product name** |
| --- | --- |
| 71967 | nordimet 15mg/0.6ml solution for injection pre-filled pens (nordic pharma ltd) |
| 53175 | neoral 25mg capsules (mawdsley-brooks & company ltd) |
| 76369 | methofill 22.5mg/0.45ml solution for injection pre-filled injector (accord healthcare ltd) |
| 34894 | sulfasalazine 500mg gastro-resistant tablet (ceretron ltd) |
| 14395 | imuran 50mg powder for solution for injection vials (aspen pharma trading ltd) |
| 14348 | metoject 20mg/2ml solution for injection pre-filled syringes (medac uk) |
| 41086 | methotrexate 5g/50ml solution for infusion vials |
| 32111 | methotrexate 2.5mg tablets (pfizer ltd) |
| 75003 | methotrexate 5g/200ml solution for infusion vials (advanz pharma) |
| 52615 | neoral 100mg capsules (sigma pharmaceuticals plc) |
| 47102 | capimune 50mg capsules (mylan) |
| 47042 | ciclosporin 100mg capsule (hillcross pharmaceuticals ltd) |
| 11959 | distamine 50mg tablet (alliance pharmaceuticals ltd) |
| 33682 | sulfasalazine 500mg gastro-resistant tablet (ddsa pharmaceuticals ltd) |
| 75698 | azathioprine 50mg tablets (phoenix healthcare distribution ltd) |
| 72495 | nordimet 22.5mg/0.9ml solution for injection pre-filled pens (nordic pharma ltd) |
| 68955 | methotrexate 10mg tablets (sandoz ltd) |
| 53989 | salazopyrin en-tabs 500mg (waymade healthcare plc) |
| 4418 | salazopyrin 500mg suppository (pharmacia ltd) |
| 45023 | quinoric 200mg tablets (bristol laboratories ltd) |
| 52868 | penicillamine 250mg tablets (phoenix healthcare distribution ltd) |
| 8583 | methotrexate 25mg/ml injection |
| 21889 | methotrexate 25mg/1ml |
| 66785 | vanquoral 10mg capsules (teva uk ltd) |
| 40280 | metoject 7.5mg/0.15ml solution for injection pre-filled syringes (medac uk) |
| 49958 | neoral 25mg capsules (de pharmaceuticals) |
| 672 | hydroxychloroquine 200mg tablets |
| 67523 | hydroxychloroquine 200mg/5ml oral suspension |
| 54867 | ciclosporin 50mg capsules (sigma pharmaceuticals plc) |
| 53797 | azathioprine 50mg tablets (arrow generics ltd) |
| 18460 | arava 100mg tablets (sanofi) |
| 71099 | leflunomide 10mg tablets (aspire pharma ltd) |
| 70119 | methotrexate 17.5mg/0.7ml solution for injection pre-filled disposable devices |
| 33601 | metoject 25mg/2.5ml solution for injection pre-filled syringes (medac uk) |
| 61160 | azathioprine 50mg tablets (tillomed laboratories ltd) |
| 29340 | azathioprine 50mg tablets (ivax pharmaceuticals uk ltd) |
| 48061 | penicillamine oral liquid |
| 74593 | methotrexate 25mg/5ml oral solution |
| 53166 | sodium aurothiomalate 50mg/0.5ml injection |
| 72944 | sulfasalazine 500mg gastro-resistant tablets (de pharmaceuticals) |
| 46039 | methotrexate 30mg/1.5ml solution for injection pre-filled syringes |
| 61419 | metoject pen 22.5mg/0.45ml solution for injection pre-filled pen (medac uk) |
| 61137 | methotrexate 17.5mg/0.35ml solution for injection pre-filled disposable devices |
| 3697 | sulfasalazine 500mg suppositories |
| 68599 | methotrexate 500mg/20ml solution for injection vials (pfizer ltd) |
| 69850 | methotrexate 10mg/0.4ml solution for injection pre-filled disposable devices |
| 66143 | hydroxychloroquine 200mg tablets (teva uk ltd) |
| 17035 | methotrexate 2.5mg/5ml oral suspension |
| 40292 | metoject 20mg/0.4ml solution for injection pre-filled syringes (medac uk) |
| 48556 | ciclosporin 100mg capsules (phoenix healthcare distribution ltd) |
| 72980 | azathioprine 25mg/5ml oral suspension |
| 47192 | capsorin 100mg capsules (morningside healthcare ltd) |
| 10211 | salazopyrin 500mg suppositories (pfizer ltd) |
| 19072 | oprisine 50mg tablet (opus pharmaceuticals ltd) |
| 61178 | metoject pen 7.5mg/0.15ml solution for injection pre-filled pen (medac uk) |
| 7337 | methotrexate 10mg/0.4ml solution for injection pre-filled syringes |
| 71898 | nordimet 20mg/0.8ml solution for injection pre-filled pens (nordic pharma ltd) |
| 21753 | maxtrex 10mg tablets (pfizer ltd) |
| 36167 | methotrexate 1g/10ml solution for injection vials |
| 32229 | methotrexate 500mg/20ml solution for injection vials |
| 58953 | penicillamine 250mg tablets (waymade healthcare plc) |
| 59250 | ciclosporin 100mg capsules (colorama pharmaceuticals ltd) |
| 8392 | penicillamine 250 mg cap |
| 7497 | sulfasalazine 250mg/5ml oral solution |
| 62596 | sulfasalazine 500mg gastro-resistant tablets (sigma pharmaceuticals plc) |
| 35402 | methotrexate 7.5mg/0.75ml solution for injection pre-filled syringes |
| 54257 | penicillamine 250mg tablets (alliance healthcare (distribution) ltd) |
| 40356 | metoject 10mg/0.2ml solution for injection pre-filled syringes (medac uk) |
| 451 | azathioprine 25mg tablets |
| 16522 | arava 10mg tablets (sanofi) |
| 15373 | sulfasalazine 500mg suppositories |
| 57441 | methotrexate 10mg tablets (a a h pharmaceuticals ltd) |
| 27404 | methotrexate 15mg/1.5ml solution for injection pre-filled syringes |
| 57183 | penicillamine 125mg tablets (a a h pharmaceuticals ltd) |
| 61171 | metoject pen 15mg/0.3ml solution for injection pre-filled pen (medac uk) |
| 67597 | methotrexate 25mg/ml injection (dupont pharmaceuticals ltd) |
| 34687 | azathioprine 50mg tablets (a a h pharmaceuticals ltd) |
| 60689 | penicillamine 125mg/5ml oral solution |
| 61081 | methotrexate 12.5mg/0.25ml solution for injection pre-filled disposable devices |
| 48798 | ciclosporin 50mg capsules (phoenix healthcare distribution ltd) |
| 643 | penicillamine 125mg tablets |
| 671 | imuran 25mg tablet (wellcome medical division) |
| 41620 | azathioprine 50mg tablets (teva uk ltd) |
| 36792 | azathioprine 250mg/5ml oral solution |
| 30780 | methotrexate 2.5mg tablet (pharmacia ltd) |
| 571 | azathioprine 50mg tablets |
| 55858 | azathioprine oral solution |
| 34451 | azathioprine 50mg tablets (mylan) |
| 73401 | methofill 7.5mg/0.15ml solution for injection pre-filled injector (accord healthcare ltd) |
| 20862 | sulfasalazine 500mg gastro-resistant tablets (actavis uk ltd) |
| 27642 | methotrexate 27.5mg/1.1ml solution for injection pre-filled syringes |
| 63798 | ciclosporin 100mg capsules (a a h pharmaceuticals ltd) |
| 56037 | methotrexate 2.5mg tablets (a a h pharmaceuticals ltd) |
| 31949 | sulfasalazine 500mg tablets (actavis uk ltd) |
| 47471 | capsorin 25mg capsules (morningside healthcare ltd) |
| 68643 | azathioprine 175mg/5ml oral suspension |
| 42178 | sulfasalazine 500mg tablet (berk pharmaceuticals ltd) |
| 7336 | methotrexate 12.5mg/0.5ml solution for injection pre-filled syringes |
| 13556 | sandimmun 100mg capsules (novartis pharmaceuticals uk ltd) |
| 75546 | sulfasalazine 250mg/5ml oral suspension sugar free (waymade healthcare plc) |
| 32101 | azathioprine 25mg tablets (a a h pharmaceuticals ltd) |
| 73486 | methotrexate 2.5mg/ml injection (dupont pharmaceuticals ltd) |
| 51181 | azathioprine 60mg/5ml oral solution |
| 267 | penicillamine 50mg tablets |
| 56909 | sulfasalazine 500mg tablets (phoenix healthcare distribution ltd) |
| 59538 | methotrexate 10mg tablets (teva uk ltd) |
| 2837 | ciclosporin 50mg capsules |
| 42637 | deximune 25mg capsules (dexcel-pharma ltd) |
| 46197 | metoject 22.5mg/0.45ml solution for injection pre-filled syringes (medac uk) |
| 4971 | leflunomide 10mg tablets |
| 42448 | deximune 50mg capsules (dexcel-pharma ltd) |
| 40273 | methotrexate 20mg/0.4ml solution for injection pre-filled syringes |
| 270 | azathioprine 50mg powder for solution for injection vials |
| 2920 | sulfasalazine 500mg tablet |
| 67163 | hydroxychloroquine 200mg tablets (phoenix healthcare distribution ltd) |
| 13320 | azathioprine 10mg tablets |
| 24634 | methotrexate 25mg/2.5ml solution for injection pre-filled syringes |
| 27400 | metoject 15mg/1.5ml solution for injection pre-filled syringes (medac uk) |
| 58885 | methotrexate 10mg tablets (sigma pharmaceuticals plc) |
| 58671 | sulfasalazine 500mg tablets (almus pharmaceuticals ltd) |
| 68672 | leflunomide 10mg tablets (alliance healthcare (distribution) ltd) |
| 3329 | myocrisin 20mg/0.5ml solution for injection ampoules (sanofi) |
| 47047 | capsorin 50mg capsules (morningside healthcare ltd) |
| 30495 | imuran 10mg tablet (wellcome medical division) |
| 35518 | azathioprine 50mg/5ml oral suspension |
| 67421 | azathioprine 100mg/5ml oral suspension |
| 31215 | azathioprine 50mg tablets (kent pharmaceuticals ltd) |
| 60979 | methotrexate 2.5mg tablets (morningside healthcare ltd) |
| 37117 | metoject 10mg/1ml solution for injection pre-filled syringes (medac uk) |
| 46637 | capimune 25mg capsules (mylan) |
| 59006 | azathioprine 25mg tablets (kent pharmaceuticals ltd) |
| 15556 | azathioprine 125 mg tab |
| 16137 | neoral 10mg capsules (novartis pharmaceuticals uk ltd) |
| 16519 | methotrexate 25mg/1ml solution for injection pre-filled syringes |
| 54982 | azathioprine 20mg/5ml oral suspension |
| 42988 | imuran 50mg tablets (aspen pharma trading ltd) |
| 17642 | arava 20mg tablets (sanofi) |
| 31683 | sulfasalazine 3g/100ml retention enema |
| 58303 | methotrexate 2.5mg tablets (orion pharma (uk) ltd) |
| 40170 | penicillamine 125mg tablets (mylan) |
| 16570 | methotrexate 7.5mg/0.3ml solution for injection pre-filled syringes |
| 42449 | deximune 100mg capsules (dexcel-pharma ltd) |
| 19370 | ciclosporin 50mg/ml concentrate solution infusion |
| 32418 | kineret 100mg/0.67ml solution for injection pre-filled syringes (swedish orphan biovitrum ltd) |
| 55116 | ciclosporin 25mg capsules (cubic pharmaceuticals ltd) |
| 40328 | methotrexate 25mg/0.5ml solution for injection pre-filled syringes |
| 27579 | methotrexate |
| 49244 | sulfasalazine 250mg/5ml oral suspension sugar free |
| 370 | salazopyrin 3g/100ml enema (pharmacia ltd) |
| 55395 | sulfasalazine 500mg tablets (waymade healthcare plc) |
| 72452 | methofill 10mg/0.2ml solution for injection pre-filled injector (accord healthcare ltd) |
| 39115 | azathioprine 10mg capsules |
| 61169 | metoject pen 25mg/0.5ml solution for injection pre-filled pen (medac uk) |
| 45165 | methotrexate 20mg/1ml solution for injection pre-filled syringes |
| 70736 | zlatal 7.5mg/0.3ml solution for injection pre-filled syringes (nordic pharma ltd) |
| 28199 | penicillamine 500 mg sus |
| 49951 | methotrexate 2.5mg tablets (sandoz ltd) |
| 40293 | metoject 25mg/0.5ml solution for injection pre-filled syringes (medac uk) |
| 14054 | salazopyrin 250mg/5ml oral suspension (pfizer ltd) |
| 61082 | methotrexate 22.5mg/0.45ml solution for injection pre-filled disposable devices |
| 46265 | metoject 17.5mg/0.35ml solution for injection pre-filled syringes (medac uk) |
| 4231 | neoral 50mg capsules (novartis pharmaceuticals uk ltd) |
| 60606 | sulfasalazine 500mg gastro-resistant tablets (sigma pharmaceuticals plc) |
| 3267 | myocrisin 50mg/0.5ml solution for injection ampoules (sanofi) |
| 75063 | azathioprine 10mg/5ml oral solution |
| 29069 | methotrexate 500mg/vial sterile powder |
| 70674 | nordimet 25mg/1ml solution for injection pre-filled pens (nordic pharma ltd) |
| 44183 | sulfasalazine 500mg gastro-resistant tablets (a a h pharmaceuticals ltd) |
| 72496 | nordimet 7.5mg/0.3ml solution for injection pre-filled pens (nordic pharma ltd) |
| 36849 | methotrexate 10mg/5ml oral suspension |
| 18890 | methotrexate 17.5mg/0.7ml solution for injection pre-filled syringes |
| 47377 | ciclosporin 25mg capsule (hillcross pharmaceuticals ltd) |
| 61122 | methotrexate 25mg/0.5ml solution for injection pre-filled disposable devices |
| 73648 | leflunomide 10mg tablets (actavis uk ltd) |
| 36726 | anakinra 100mg/0.67ml solution for injection pre-filled syringes |
| 71054 | sulfasalazine 500mg gastro-resistant tablets (teva uk ltd) |
| 770 | azathioprine capsules |
| 1566 | salazopyrin 500mg tablet (pharmacia ltd) |
| 66487 | zlatal 12.5mg/0.5ml solution for injection pre-filled syringes (nordic pharma ltd) |
| 67739 | leflunomide 20mg tablets (teva uk ltd) |
| 34258 | methotrexate 20mg/0.8ml injection (central homecare) |
| 51120 | methotrexate 2.5mg tablets (alliance healthcare (distribution) ltd) |
| 3327 | distamine 125mg tablets (alliance pharmaceuticals ltd) |
| 31667 | sulfasalazine 500mg tablets (a a h pharmaceuticals ltd) |
| 49243 | sulfasalazine 500mg/5ml oral suspension |
| 61796 | methotrexate 30mg/0.6ml solution for injection pre-filled disposable devices |
| 61151 | methotrexate 10mg/0.2ml solution for injection pre-filled disposable devices |
| 64858 | vanquoral 50mg capsules (teva uk ltd) |
| 57174 | methotrexate 10mg tablets (waymade healthcare plc) |
| 43077 | imuran 25mg tablets (aspen pharma trading ltd) |
| 3896 | ciclosporin 100mg capsules |
| 36800 | methotrexate 10mg/5ml oral solution |
| 41585 | methotrexate sodium 2.5mg tablet (wyeth pharmaceuticals) |
| 62421 | metoject pen 27.5mg/0.55ml solution for injection pre-filled pen (medac uk) |
| 29566 | hydroxychloroquine 200mg/5ml oral solution |
| 14748 | methotrexate sodium 25mg/ml injection |
| 72486 | nordimet 10mg/0.4ml solution for injection pre-filled pens (nordic pharma ltd) |
| 61140 | methotrexate 20mg/0.4ml solution for injection pre-filled disposable devices |
| 46098 | metoject 12.5mg/0.25ml solution for injection pre-filled syringes (medac uk) |
| 61050 | metoject pen 10mg/0.2ml solution for injection pre-filled pen (medac uk) |
| 22982 | azathioprine 50mg/5ml oral solution |
| 30925 | penicillamine 250mg tablets (actavis uk ltd) |
| 61084 | sulfasalazine 500mg gastro-resistant tablets (kent pharmaceuticals ltd) |
| 61180 | metoject pen 20mg/0.4ml solution for injection pre-filled pen (medac uk) |
| 823 | methotrexate 2.5mg tablets |
| 283 | myocrisin 10mg/0.5ml solution for injection ampoules (sanofi) |
| 1899 | imuran 50mg tablet (wellcome medical division) |
| 43562 | azathioprine 50mg tablets (actavis uk ltd) |
| 33968 | sulfasalazine 500mg tablet (approved prescription services ltd) |
| 59099 | sulfasalazine 500mg tablets (alliance healthcare (distribution) ltd) |
| 380 | salazopyrin en-tabs 500mg (pfizer ltd) |
| 66094 | hydroxychloroquine 200mg tablets (de pharmaceuticals) |
| 12339 | azamune 50mg tablet (penn pharmaceuticals ltd) |
| 48217 | leflunomide 10mg tablets (medac uk) |
| 604 | penicillamine 250mg tablets |
| 53696 | methotrexate 50mg/2ml solution for injection vials (a a h pharmaceuticals ltd) |
| 63298 | hydroxychloroquine 200mg tablets (creo pharma ltd) |
| 46407 | methotrexate 1g/40ml solution for injection vials |
| 13493 | ridaura tiltab 3mg tablets (astellas pharma ltd) |
| 72300 | nordimet 12.5mg/0.5ml solution for injection pre-filled pens (nordic pharma ltd) |
| 17880 | salazopyrin 250mg/5ml liquid (pharmacia ltd) |
| 30932 | methotrexate 5mg/0.2ml solution for injection pre-filled syringes |
| 13494 | sandimmun 100mg/ml oral solution (novartis pharmaceuticals uk ltd) |
| 4946 | plaquenil 200mg tablets (sanofi) |
| 13321 | salazopyrin 3g/100ml enema (pfizer ltd) |
| 3918 | azathioprine 10 mg tab |
| 72453 | methofill 20mg/0.4ml solution for injection pre-filled injector (accord healthcare ltd) |
| 35865 | metoject 7.5mg/0.75ml solution for injection pre-filled syringes (medac uk) |
| 75012 | azathioprine 50mg tablet (berk pharmaceuticals ltd) |
| 4978 | salazopyrin 500mg tablets (pfizer ltd) |
| 2838 | ciclosporin 25mg capsules |
| 72045 | methotrexate 10mg tablets (morningside healthcare ltd) |
| 54975 | ciclosporin 100mg capsules (cubic pharmaceuticals ltd) |
| 8327 | methotrexate 50mg/3ml injection |
| 15596 | sandimmun 50mg capsules (novartis pharmaceuticals uk ltd) |
| 359 | sulfasalazine 3g/100ml enema |
| 68671 | leflunomide 20mg tablets (alliance healthcare (distribution) ltd) |
| 45558 | methotrexate 25mg/1.25ml solution for injection pre-filled syringes |
| 65339 | azapress 50mg tablets (ennogen pharma ltd) |
| 1905 | neoral 100mg/ml oral solution (novartis pharmaceuticals uk ltd) |
| 973 | neoral 100mg capsules (novartis pharmaceuticals uk ltd) |
| 73323 | penicillamine 250mg tablet (ivax pharmaceuticals uk ltd) |
| 50950 | metoject 30mg/0.6ml solution for injection pre-filled syringes (medac uk) |
| 75803 | methofill 12.5mg/0.25ml solution for injection pre-filled injector (accord healthcare ltd) |
| 54974 | ciclosporin 50mg capsules (cubic pharmaceuticals ltd) |
| 44908 | methotrexate 30mg/0.6ml solution for injection pre-filled syringes |
| 56872 | penicillamine 125mg tablets (phoenix healthcare distribution ltd) |
| 70059 | methotrexate 25mg/1ml solution for injection pre-filled disposable devices |
| 40284 | metoject 15mg/0.3ml solution for injection pre-filled syringes (medac uk) |
| 5427 | sulfasalazine 500mg tablets |
| 8904 | distamine 250mg tablets (alliance pharmaceuticals ltd) |
| 54193 | hydroxychloroquine 200mg tablets (a a h pharmaceuticals ltd) |
| 23401 | sulazine ec 500mg tablets (genesis pharmaceuticals ltd) |
| 31217 | penicillamine 250mg tablets (a a h pharmaceuticals ltd) |
| 54134 | ciclosporin 100mg capsules (sigma pharmaceuticals plc) |
| 69639 | methotrexate 7.5mg/0.3ml solution for injection pre-filled disposable devices |
| 71048 | sulfasalazine 500mg tablets (genesis pharmaceuticals ltd) |
| 70379 | zlatal 10mg/0.4ml solution for injection pre-filled syringes (nordic pharma ltd) |
| 51667 | methotrexate 200mg/8ml solution for injection vials |
| 34473 | sulfasalazine 500mg tablets (mylan) |
| 26064 | methotrexate 20mg/2ml solution for injection pre-filled syringes |
| 49547 | methotrexate 5g/200ml solution for infusion vials |
| 40371 | methotrexate 10mg/0.2ml solution for injection pre-filled syringes |
| 15344 | sodium aurothiomalate 2 mg inj |
| 48012 | hydroxychloroquine sulphate 200mg tablet (hillcross pharmaceuticals ltd) |
| 70977 | methotrexate 12mg/5ml oral suspension |
| 68631 | methotrexate 5mg/2ml solution for injection vials (pfizer ltd) |
| 66473 | vanquoral 25mg capsules (teva uk ltd) |
| 46152 | methotrexate 12.5mg/0.25ml solution for injection pre-filled syringes |
| 66003 | azathioprine 25mg tablets (mawdsley-brooks & company ltd) |
| 70046 | methotrexate 15mg/0.6ml solution for injection pre-filled disposable devices |
| 34929 | methotrexate 10mg tablets (pfizer ltd) |
| 1626 | ciclosporin 100mg/ml oral solution sugar free |
| 61273 | methotrexate 15mg/0.3ml solution for injection pre-filled disposable devices |
| 31120 | penicillamine 125mg tablet (ivax pharmaceuticals uk ltd) |
| 73677 | methofill 25mg/0.5ml solution for injection pre-filled injector (accord healthcare ltd) |
| 41104 | methotrexate 2.5mg tablets (wockhardt uk ltd) |
| 17206 | azathioprine 50 mg sus |
| 64857 | vanquoral 100mg capsules (teva uk ltd) |
| 21899 | immunoprin 50mg tablets (ashbourne pharmaceuticals ltd) |
| 14347 | methotrexate 20mg/0.8ml solution for injection pre-filled syringes |
| 28041 | methotrexate 12.5mg/5ml oral suspension |
| 73434 | methotrexate 2mg/ml oral solution sugar free (rosemont pharmaceuticals ltd) |
| 9528 | methotrexate 5mg/2ml solution for injection vials |
| 12816 | methotrexate 100mg/ml injection |
| 76367 | methotrexate 2.5mg tablets (almus pharmaceuticals ltd) |
| 16035 | ciclosporin 10mg capsules |
| 59312 | penicillamine 125mg tablets (kent pharmaceuticals ltd) |
| 52743 | ciclosporin 25mg capsules (sigma pharmaceuticals plc) |
| 59249 | ciclosporin 50mg capsules (colorama pharmaceuticals ltd) |
| 27342 | maxtrex 2.5mg/ml injection (pharmacia ltd) |
| 18424 | methotrexate sodium 2.5mg tablet |
| 61488 | metoject pen 30mg/0.6ml solution for injection pre-filled pen (medac uk) |
| 73010 | hydroxychloroquine 40mg/5ml oral solution |
| 61211 | metoject pen 12.5mg/0.25ml solution for injection pre-filled pen (medac uk) |
| 3920 | sandimmun 25mg capsules (novartis pharmaceuticals uk ltd) |
| 65584 | methotrexate 2mg/ml oral solution sugar free |
| 16606 | sodium aurothiomalate 20mg/0.5ml solution for injection ampoules |
| 11767 | sulfasalazine 250mg/5ml oral suspension |
| 53385 | methotrexate 2.5mg tablets (waymade healthcare plc) |
| 53176 | neoral 50mg capsules (de pharmaceuticals) |
| 972 | neoral 25mg capsules (novartis pharmaceuticals uk ltd) |
| 71283 | methotrexate 22.5mg/0.9ml solution for injection pre-filled disposable devices |
| 71453 | methofill 15mg/0.3ml solution for injection pre-filled injector (accord healthcare ltd) |
| 51321 | methotrexate 50mg/2ml solution for injection vials |
| 64317 | hydroxychloroquine 200mg tablets (waymade healthcare plc) |
| 54209 | sulfasalazine 500mg gastro-resistant tablets (phoenix healthcare distribution ltd) |
| 62753 | methotrexate 27.5mg/0.55ml solution for injection pre-filled syringes |
| 73814 | azathioprine 50mg tablets (de pharmaceuticals) |
| 48763 | ciclosporin 25mg capsules (phoenix healthcare distribution ltd) |
| 57103 | metoject 27.5mg/0.55ml solution for injection pre-filled syringes (medac uk) |
| 62051 | ciclosporin 25mg capsules (niche pharma ltd) |
| 71046 | sulfasalazine 500mg tablets (de pharmaceuticals) |
| 34684 | penicillamine 250mg tablets (mylan) |
| 32865 | methotrexate 10mg/1ml solution for injection pre-filled syringes |
| 75002 | sulfasalazine 500mg gastro-resistant tablets (mawdsley-brooks & company ltd) |
| 40301 | methotrexate 7.5mg/0.15ml solution for injection pre-filled syringes |
| 52606 | methotrexate 2.5mg tablets (sigma pharmaceuticals plc) |
| 8776 | azathioprine 100 mg tab |
| 76247 | zlatal 25mg/1ml solution for injection pre-filled syringes (nordic pharma ltd) |
| 877 | methotrexate 10mg tablets |
| 71454 | zlatal 15mg/0.6ml solution for injection pre-filled syringes (nordic pharma ltd) |
| 3934 | auranofin 3mg tablets |
| 62007 | leflunomide 15mg tablets |
| 46156 | methotrexate 17.5mg/0.35ml solution for injection pre-filled syringes |
| 62833 | methotrexate 2.5mg tablets (de pharmaceuticals) |
| 61172 | methotrexate 7.5mg/0.15ml solution for injection pre-filled disposable devices |
| 40281 | methotrexate 15mg/0.3ml solution for injection pre-filled syringes |
| 35752 | methotrexate 7.5mg/5ml oral suspension |
| 61181 | metoject pen 17.5mg/0.35ml solution for injection pre-filled pen (medac uk) |
| 75868 | methofill 17.5mg/0.35ml solution for injection pre-filled injector (accord healthcare ltd) |
| 508 | sulfasalazine 500mg gastro-resistant tablets |
| 70737 | hydroxychloroquine 200mg tablets (alliance healthcare (distribution) ltd) |
| 58468 | penicillamine 125mg tablets (teva uk ltd) |
| 55773 | azathioprine 10mg/5ml oral suspension |
| 10842 | sodium aurothiomalate 10mg/0.5ml solution for injection ampoules |
| 4470 | sodium aurothiomalate 50mg/0.5ml solution for injection ampoules |
| 352 | penicillamine 125 mg cap |
| 30703 | methotrexate 30mg/1.2ml solution for injection pre-filled syringes |
| 29721 | pendramine 125mg tablet (viatris pharmaceuticals ltd) |
| 38056 | ciclosporin 50mg/1ml solution for infusion ampoules |
| 20951 | methotrexate 2.5mg tablets (advanz pharma) |
| 42924 | ciclosporin 250mg/5ml solution for infusion ampoules |
| 52921 | azathioprine 125mg/5ml oral suspension |
| 17672 | methotrexate 22.5mg/0.9ml solution for injection pre-filled syringes |
| 13428 | maxtrex 2.5mg tablets (pfizer ltd) |
| 4970 | leflunomide 100mg tablets |
| 64836 | hydroxychloroquine 150mg/5ml oral suspension |
| 16540 | methotrexate 15mg/0.6ml solution for injection pre-filled syringes |
| 34816 | azathioprine 25mg tablets (mylan) |
| 59723 | methotrexate 7.5mg/5ml oral solution |
| 64130 | penicillamine 125mg tablets (waymade healthcare plc) |
| 55433 | penicillamine 250mg tablets (kent pharmaceuticals ltd) |
| 24783 | methotrexate 50mg/2ml injection |
| 57789 | penicillamine 125mg tablets (alliance healthcare (distribution) ltd) |
| 68977 | azathioprine 25mg tablets (sigma pharmaceuticals plc) |
| 58654 | azathioprine 50mg tablets (sandoz ltd) |
| 64131 | hydroxychloroquine 200mg tablets (zentiva) |
| 20255 | pendramine 250mg tablet (viatris pharmaceuticals ltd) |
| 26261 | berkaprine 50mg tablet (rorer pharmaceuticals ltd) |
| 70411 | methotrexate 20mg/0.8ml solution for injection pre-filled disposable devices |
| 41670 | azathioprine 50mg tablet (c p pharmaceuticals ltd) |
| 69928 | methotrexate 12.5mg/0.5ml solution for injection pre-filled disposable devices |
| 46129 | methotrexate 22.5mg/0.45ml solution for injection pre-filled syringes |
| 61085 | methotrexate 2.5mg tablets (waymade healthcare plc) |
| 74812 | imuran 25mg tablets (stephar (u.k.) ltd) |
| 72645 | leflunomide 20mg tablets (actavis uk ltd) |
| 62993 | leflunomide 20mg tablets (sandoz ltd) |
| 46395 | capimune 100mg capsules (mylan) |
| 65639 | ciclosporin 100mg capsules (j m mcgill ltd) |
| 53869 | azathioprine 20mg/5ml oral solution |
| 59685 | methotrexate 2.5mg tablets (teva uk ltd) |
| 74213 | leflunomide 20mg tablets (a a h pharmaceuticals ltd) |
| 63121 | azathioprine 25mg tablets (alliance healthcare (distribution) ltd) |
| 31216 | penicillamine 125mg tablets (actavis uk ltd) |
| 6934 | leflunomide 20mg tablets |
| 61726 | sulazine ec 500mg tablets (teva uk ltd) |
| 71619 | nordimet 17.5mg/0.7ml solution for injection pre-filled pens (nordic pharma ltd) |
| 26790 | sandimmun 50mg/ml concentrate for solution for infusion (novartis pharmaceuticals uk ltd) |
| 53956 | azathioprine 50mg tablets (almus pharmaceuticals ltd) |

Smoking status - medcodes

| **medcode** | **Description** | **Ever or never smoker** |
| --- | --- | --- |
| 33 | Never smoked tobacco | Never |
| 60 | Current non-smoker | Never |
| 90 | Ex smoker | Ever |
| 93 | Cigarette smoker | Ever |
| 776 | Stopped smoking | Ever |
| 1822 | Very heavy smoker - 40+cigs/d | Ever |
| 1823 | Smoker | Ever |
| 1878 | Moderate smoker - 10-19 cigs/d | Ever |
| 3568 | Heavy smoker - 20-39 cigs/day | Ever |
| 7622 | Smoking cessation advice | Ever |
| 9045 | Advice on smoking | Ever |
| 10184 | Pregnancy smoking advice | Ever |
| 10211 | Smoking cessation milestones | Ever |
| 10558 | Current smoker | Ever |
| 10742 | Referral to stop-smoking clinic | Ever |
| 11356 | Seen by smoking cessation advisor | Ever |
| 11527 | DNA - Did not attend smoking cessation clinic | Ever |
| 11788 | Non-smoker | Never |
| 12240 | Trying to give up smoking | Ever |
| 12878 | Date ceased smoking | Ever |
| 12941 | Occasional smoker | Ever |
| 12942 | Smoker - amount smoked | Ever |
| 12943 | Cigar smoker | Ever |
| 12944 | Light smoker - 1-9 cigs/day | Ever |
| 12946 | Ex-smoker - amount unknown | Ever |
| 12947 | Pipe smoker | Ever |
| 12951 | Smoking restarted | Ever |
| 12952 | Smoking started | Ever |
| 12953 | Attends stop smoking monitor. | Ever |
| 12955 | Ex-moderate smoker (10-19/day) | Ever |
| 12956 | Ex-heavy smoker (20-39/day) | Ever |
| 12957 | Ex-light smoker (1-9/day) | Ever |
| 12958 | Trivial smoker - < 1 cig/day | Ever |
| 12959 | Ex-very heavy smoker (40+/day) | Ever |
| 12961 | Ex-trivial smoker (<1/day) | Ever |
| 12964 | Keeps trying to stop smoking | Ever |
| 12966 | Smoking reduced | Ever |
| 16717 | Smokers' cough | Ever |
| 18573 | Referral to smoking cessation advisor | Ever |
| 19488 | Ex cigar smoker | Ever |
| 26096 | Smokes drugs | Ever |
| 26470 | Ex pipe smoker | Ever |
| 30423 | Thinking about stopping smoking | Ever |
| 30762 | Not interested in stopping smoking | Ever |
| 31114 | Ready to stop smoking | Ever |
| 34126 | Negotiated date for cessation of smoking | Ever |
| 38112 | Smoking cessation programme start date | Ever |
| 40418 | Refuses stop smoking monitor | Ever |
| 41042 | Smoking cessation advice provided by community pharmacist | Ever |
| 41979 | Smoking restarted | Ever |
| 74907 | Smoking cessation therapy | Ever |
| 90522 | Smoking cessation therapy NOS | Ever |
| 91708 | Other specified smoking cessation therapy | Ever |
| 94958 | Smoking cessation drug therapy | Ever |
| 97210 | Ex-cigarette smoker | Ever |
| 98137 | Brief intervention for smoking cessation | Ever |
| 98154 | Referral to NHS stop smoking service | Ever |
| 98245 | Stop smoking face to face follow-up | Ever |
| 99838 | Recently stopped smoking | Ever |
| 100099 | Smoking cessation advice declined | Ever |
| 100495 | Ex roll-up cigarette smoker | Ever |
| 101338 | Failed attempt to stop smoking | Ever |
| 101764 | Practice based smoking cessation programme start date | Ever |
| 102361 | Referral for smoking cessation service offered | Ever |
| 102951 | Lost to smoking cessation follow-up | Ever |
| 103507 | Stop smoking service opportunity signposted | Ever |
| 104185 | Smoking cessation drug therapy declined | Ever |
| 104230 | Smoking cessation programme declined | Ever |
| 105999 | Smokes drugs in cigarette form | Ever |

Smoking status – Product codes

| **prodcode** | **Product name** |
| --- | --- |
| 42221 | Nicotine 4mg lozenges sugar free (Teva UK Ltd) |
| 38958 | Nicotinell 1mg lozenges (Novartis Consumer Health UK Ltd) |
| 41765 | Nicotinell Mint 2mg medicated chewing gum (Novartis Consumer Health UK Ltd) |
| 5502 | Nicotine 15mg/16hours transdermal patches |
| 39123 | Nicotine 25mg/16hours transdermal patches |
| 58034 | Nicotine 2.5mg orodispersible films sugar free |
| 5784 | Nicotine 4mg lozenges sugar free |
| 42047 | Nicotinell Liquorice 4mg medicated chewing gum (Novartis Consumer Health UK Ltd) |
| 5457 | Nicotine 5mg/16hours transdermal patches |
| 41860 | Nicotine bitartrate 2mg Sublingual tablet |
| 27414 | Varenicline 1mg tablets |
| 41923 | Nicotine 15mg/16 hours transdermal patches and Nicotine 2mg medicated chewing gum sugar free |
| 5515 | Nicotine 1mg Lozenge |
| 46717 | Nicotine 15mg inhalation cartridges with device |
| 14556 | TRANSDERMAL NICOTINE PATCH 10 MG |
| 5606 | Nicotinell tts 20 sq cm Transdermal patch (Novartis Consumer Health UK Ltd) |
| 40617 | Nicotinell TTS 20 patches (Novartis Consumer Health UK Ltd) |
| 3818 | Nicotinell tts 30 sq cm Transdermal patch (Novartis Consumer Health UK Ltd) |
| 25510 | Nicotine 2mg mint flavour chewing-gum |
| 37646 | Nicotine 1.5mg lozenges sugar free |
| 41879 | Nicotinell Liquorice 2mg medicated chewing gum (Novartis Consumer Health UK Ltd) |
| 5946 | Nicotinell 2mg Medicated chewing-gum (Novartis Consumer Health UK Ltd) |
| 45504 | Nicotine 1mg/dose oromucosal spray sugar free |
| 6448 | Nicotine 21mg/24hours transdermal patches |
| 8297 | NICOTINE TRANSDERMAL PATCH 20CM |
| 42048 | Nicotine bitartrate 1mg lozenges sugar free |
| 33392 | Nicotine 22mg/24 hr Transdermal patch |
| 41909 | Nicotinell Mint 4mg medicated chewing gum (Novartis Consumer Health UK Ltd) |
| 8534 | TRANSDERMAL NICOTINE PATCH 5 MG |
| 5944 | Nicotine 10mg inhalation cartridges with device |
| 6698 | Nicotinell 2mg lozenges (Novartis Consumer Health UK Ltd) |
| 41881 | Nicotinell Classic 2mg medicated chewing gum (Novartis Consumer Health UK Ltd) |
| 9591 | Nicotine 14mg/24hours transdermal patches |
| 5758 | Nicotine 4mg medicated chewing gum sugar free |
| 116 | NICOTINE TRANSDERMAL PATCH 30CM |
| 8571 | Nicotine 500micrograms/dose nasal spray |
| 27412 | Varenicline 1mg tablets and Varenicline 500microgram tablets |
| 11718 | Nicotine 2mg sublingual tablets sugar free |
| 13048 | Nicotinell 4mg Medicated chewing-gum (Novartis Consumer Health UK Ltd) |
| 5531 | Nicotinell 1mg Lozenge (Novartis Consumer Health UK Ltd) |
| 41808 | Nicotinell Fruit 4mg medicated chewing gum (Novartis Consumer Health UK Ltd) |
| 5115 | Bupropion 150mg modified-release tablets |
| 7303 | Nicotinell tts 10 sq cm Transdermal patch (Novartis Consumer Health UK Ltd) |
| 25516 | Nicotine 4mg mint flavour chewing-gum |
| 42011 | Nicotinell Classic 4mg medicated chewing gum (Novartis Consumer Health UK Ltd) |
| 46588 | Nicotinell Icemint 2mg medicated chewing gum (Novartis Consumer Health UK Ltd) |
| 40683 | Nicotinell TTS 10 patches (Novartis Consumer Health UK Ltd) |
| 46701 | Nicotinell Icemint 4mg medicated chewing gum (Novartis Consumer Health UK Ltd) |
| 41931 | Nicotinell Fruit 2mg medicated chewing gum (Novartis Consumer Health UK Ltd) |
| 9806 | Nicotine 2mg lozenges sugar free |
| 35089 | Varenicline 500microgram tablets |
| 9804 | Nicotine 7mg/24hours transdermal patches |
| 42286 | Nicotine bitartrate 2mg lozenges sugar free |
| 6323 | Nicotine 2mg medicated chewing gum sugar free |
| 40620 | Nicotinell TTS 30 patches (Novartis Consumer Health UK Ltd) |
| 55590 | Nicotine 11mg/24 hr Transdermal patch |
| 5479 | Nicotine 10mg/16hours transdermal patches |

NSAIDs – product codes

| **prodcode** | **Product name** |
| --- | --- |
| 33669 | Diclofenac 50mg Gastro-resistant tablet (Genus Pharmaceuticals Ltd) |
| 32601 | Econac 100mg suppositories (Mercury Pharma Group Ltd) |
| 3216 | Indometacin 25mg modified-release tablets |
| 33801 | Opustan 250mg Capsule (Opus Pharmaceuticals Ltd) |
| 3182 | Froben 50mg tablets (Abbott Laboratories Ltd) |
| 14678 | Defanac sr 100mg Modified-release tablet (Ranbaxy (UK) Ltd) |
| 50602 | Diclofenac potassium 50mg tablets (Alliance Healthcare (Distribution) Ltd) |
| 29037 | Valdic 100 Retard tablets (Fannin UK Ltd) |
| 36543 | Aspirin 100mg effervescent tablets |
| 2387 | Arthrotec 75 gastro-resistant tablets (Pfizer Ltd) |
| 50266 | Ibuprofen 200mg caplets (The Boots Company Plc) |
| 30164 | Lemsip Cold and Flu Sinus 12 Hr Ibuprofen + Pseudoephedrine modified-release capsules (Reckitt Benckiser Healthcare (UK) Ltd) |
| 13606 | Flexin-25 Continus tablets (Napp Pharmaceuticals Ltd) |
| 46967 | Mefenamic acid 250mg Capsule (Sandoz Ltd) |
| 17532 | Dicloflex Retard 100mg tablets (Kent Pharmaceuticals Ltd) |
| 7490 | Froben 100mg suppositories (Abbott Laboratories Ltd) |
| 35653 | Etopan XL 600mg tablets (Taro Pharmaceuticals (UK) Ltd) |
| 46848 | Naproxen 500mg Gastro-resistant tablet (Almus Pharmaceuticals Ltd) |
| 53700 | Naproxen 250mg gastro-resistant tablets (Alliance Healthcare (Distribution) Ltd) |
| 3326 | Oruvail 100mg Modified-release capsule (Hawgreen Ltd) |
| 35935 | Meloxicam 7.5mg tablets (Somex Pharma) |
| 15767 | Ibuprofen 5% foam |
| 57006 | Diclofenac sodium 25mg gastro-resistant tablets (Phoenix Healthcare Distribution Ltd) |
| 51293 | Diclofenac potassium 50mg tablets (Phoenix Healthcare Distribution Ltd) |
| 42108 | Ibuprofen 200mg tablets (OBG Pharmaceuticals Ltd) |
| 387 | Surgam 200mg tablets (Sanofi) |
| 18527 | Mandafen 400mg tablets (M & A Pharmachem Ltd) |
| 2386 | Voltarol Retard 100mg tablets (Novartis Pharmaceuticals UK Ltd) |
| 12766 | Flurbiprofen 8.75mg lozenges |
| 612 | Dicloflex 25mg gastro-resistant tablets (Dexcel-Pharma Ltd) |
| 48059 | Diclofenac potassium 50mg tablets (A A H Pharmaceuticals Ltd) |
| 736 | Indometacin 50mg capsules |
| 50058 | Voltarol 50mg dispersible tablets (Doncaster Pharmaceuticals Ltd) |
| 52905 | Aspirin 300mg tablets (Lloyds Pharmacy Ltd) |
| 51306 | Parecoxib 40mg powder for solution for injection vials |
| 29759 | Aspro Tablet (Roche Consumer Health) |
| 20016 | Tolmetin 400mg Capsule |
| 18261 | Aspirin 500mg with Papaveretum 7.71mg dispersible tablets |
| 34527 | Ibuprofen 200mg tablets (Zentiva) |
| 44483 | Nurofen Express 512mg tablets (Reckitt Benckiser Healthcare (UK) Ltd) |
| 53622 | Aspirin 300mg Tablet (M & A Pharmachem Ltd) |
| 8672 | Feldene 20mg suppositories (Pfizer Ltd) |
| 31429 | Timpron 250mg Gastro-resistant tablet (Berk Pharmaceuticals Ltd) |
| 28816 | Rheuflex 500mg Tablet (Goldshield Pharmaceuticals Ltd) |
| 34290 | Naproxen 250mg gastro-resistant tablets (Teva UK Ltd) |
| 33935 | Nurofen Maximum Strength Migraine Pain 684mg caplets (Reckitt Benckiser Healthcare (UK) Ltd) |
| 4254 | Cytotec 200microgram tablets (Pfizer Ltd) |
| 27055 | Diclofenac sodium 50mg gastro-resistant tablets (Kent Pharmaceuticals Ltd) |
| 36577 | Indometacin 50mg Capsule (Meridian Healthcare (UK) Ltd) |
| 21815 | Arthrofen 600 tablets (Ashbourne Pharmaceuticals Ltd) |
| 5175 | Celebrex 100mg capsules (Pfizer Ltd) |
| 30327 | Jomethid XL 200mg capsules (Actavis UK Ltd) |
| 33568 | Ketoprofen 200mg Modified-release capsule (Actavis UK Ltd) |
| 18151 | Voltarol Pain-eze 1% Emulgel (Novartis Consumer Health UK Ltd) |
| 5085 | Voltarol Rapid 50mg tablets (Novartis Pharmaceuticals UK Ltd) |
| 360 | Brufen 100mg/5ml syrup (Abbott Laboratories Ltd) |
| 34434 | Aspirin 75mg dispersible tablets (Thornton & Ross Ltd) |
| 55009 | Brufen 600mg effervescent granules sachets (Necessity Supplies Ltd) |
| 7481 | Lederfen 450mg Tablet (Wyeth Pharmaceuticals) |
| 24111 | Ketorolac 10mg/1ml solution for injection ampoules |
| 49132 | Voltarol 1% Emulgel (Necessity Supplies Ltd) |
| 393 | Disprin 300mg dispersible tablets (Reckitt Benckiser Healthcare (UK) Ltd) |
| 53804 | Aspirin 300mg gastro-resistant tablets (Alliance Healthcare (Distribution) Ltd) |
| 13882 | Imazin XL tablets (Napp Pharmaceuticals Ltd) |
| 42793 | Diclofenac 100mg Modified-release tablet (IVAX Pharmaceuticals UK Ltd) |
| 45988 | Ibuprofen 200mg / Phenylephrine 5mg tablets |
| 43434 | Aspirin 300mg gastro-resistant tablets (A A H Pharmaceuticals Ltd) |
| 349 | VOLTAROL 75 MG INJ |
| 2366 | Flurbiprofen 100mg tablets |
| 34595 | Mefenamic acid 500mg tablets (Zentiva) |
| 48165 | Aspirin 300mg tablets (Aspar Pharmaceuticals Ltd) |
| 42604 | Mobiflex 20mg tablets (Meda Pharmaceuticals Ltd) |
| 28172 | Ibuprofen 300mg / Pseudoephedrine 45mg modified-release capsules |
| 40381 | Aspirin 75mg Soluble tablet (C P Pharmaceuticals Ltd) |
| 526 | Aceclofenac 100mg tablets |
| 11522 | Pennsaid 1.50% Cutaneous solution (Provalis Healthcare Ltd) |
| 51614 | Ibuprofen 200mg caplets (Lloyds Pharmacy Ltd) |
| 16286 | Lofensaid Retard 75 tablets (Opus Pharmaceuticals Ltd) |
| 11554 | Ibuprofen 200mg / Codeine 12.8mg tablets |
| 1866 | Naprosyn 500mg tablets (Roche Products Ltd) |
| 25433 | Radian B Muscle lotion (Thornton & Ross Ltd) |
| 31469 | Apsifen -f 600mg Tablet (Approved Prescription Services Ltd) |
| 17818 | Ketovail 100mg modified-release capsules (Teva UK Ltd) |
| 40141 | Ketoprofen 100mg capsules (A A H Pharmaceuticals Ltd) |
| 57007 | Mefenamic acid 250mg capsules (Essential Generics Ltd) |
| 10785 | Fenbid 300mg Spansules (Mercury Pharma Group Ltd) |
| 26575 | Streflam 8.75mg Lozenge (Crookes Healthcare Ltd) |
| 57297 | Mefenamic acid 500mg tablets (Alliance Healthcare (Distribution) Ltd) |
| 26083 | Indolar 100mg Suppository (Lagap) |
| 34536 | Ibuprofen 400mg tablets (IVAX Pharmaceuticals UK Ltd) |
| 8882 | Feldene 0.50% Sports gel (Pfizer Ltd) |
| 14084 | Diclovol 75mg SR tablets (Arun Pharmaceuticals Ltd) |
| 3496 | Nycopren 250mg gastro-resistant tablets (Ardern Healthcare Ltd) |
| 56441 | Calprofen 100mg/5ml oral suspension 5ml sachets (McNeil Products Ltd) |
| 50080 | Dynastat 40mg powder and solvent for solution for injection vials (Pfizer Ltd) |
| 52856 | Co-codaprin 8mg/400mg tablets |
| 21821 | Lidifen f 600mg Tablet (Berk Pharmaceuticals Ltd) |
| 377 | Aspirin 300mg dispersible tablets |
| 54284 | Aspirin 75mg dispersible tablets (Almus Pharmaceuticals Ltd) |
| 54137 | Ibuprofen 400mg tablets (Aspar Pharmaceuticals Ltd) |
| 5254 | Celecoxib 200mg capsules |
| 32105 | Mefenamic acid 500mg tablets (A A H Pharmaceuticals Ltd) |
| 34898 | Mefenamic acid 250mg Capsule (Berk Pharmaceuticals Ltd) |
| 43032 | Inoven 200mg Tablet (Janssen-Cilag Ltd) |
| 54660 | Diclofenac sodium 50mg capsules |
| 13459 | Dysman 500 tablets (Ashbourne Pharmaceuticals Ltd) |
| 14333 | Ibuprofen 400mg capsules |
| 43426 | Ibuprofen 5% gel (A A H Pharmaceuticals Ltd) |
| 10310 | Aspirin powder |
| 21045 | Ibumetin 400mg Tablet (Alfred Benzon (UK) Ltd) |
| 13347 | Alrheumat 50mg Capsule (Bayer Plc) |
| 37541 | Aspirin 227mg medicated chewing-gum |
| 254 | Aspirin 300mg tablets |
| 928 | Diclofenac sodium 25mg tablets |
| 18820 | Fenpaed 100mg/5ml Oral suspension (Pinewood Healthcare) |
| 31954 | Aspirin 75mg dispersible tablets (Teva UK Ltd) |
| 53576 | Arcoxia 120mg tablets (Doncaster Pharmaceuticals Ltd) |
| 36260 | Mendys 250mg Capsule (Kent Pharmaceuticals Ltd) |
| 45851 | Aspirin 300mg Soluble tablet (Ranbaxy (UK) Ltd) |
| 41365 | Axorid 200mg/20mg modified-release capsules (Meda Pharmaceuticals Ltd) |
| 4965 | Piroxicam 20mg orodispersible tablets sugar free |
| 34961 | Ibuprofen 600mg tablets (Sandoz Ltd) |
| 50166 | Generic Anadin Extra tablets |
| 43541 | Piroxicam 10mg capsules (Actavis UK Ltd) |
| 20709 | MEFENAMIC ACID DISPERSIBLE |
| 26231 | Timpron 500mg Gastro-resistant tablet (Berk Pharmaceuticals Ltd) |
| 27362 | Diclofenac 100mg Modified-release tablet (Actavis UK Ltd) |
| 13380 | Clinoril 200mg tablets (Merck Sharp & Dohme Ltd) |
| 55582 | Celebrex 200mg capsules (Lexon (UK) Ltd) |
| 12964 | Aspirin 600mg / Caffeine 50mg oral powder sachets sugar free |
| 14385 | Cuprofen 200mg Tablet (SSL International Plc) |
| 18798 | Lofensaid 50mg gastro-resistant tablets (Opus Pharmaceuticals Ltd) |
| 9822 | Arcoxia 120mg tablets (Merck Sharp & Dohme Ltd) |
| 41623 | Piroxicam 20mg capsules (IVAX Pharmaceuticals UK Ltd) |
| 9886 | Dicloflex 50mg Gastro-resistant tablet (Ratiopharm UK Ltd) |
| 3043 | Ketoprofen 200mg modified-release capsules |
| 21843 | Pranoxen continus 375mg Tablet (Napp Pharmaceuticals Ltd) |
| 17491 | Dicloflex sr 75mg Tablet (Ratiopharm UK Ltd) |
| 10898 | Voltarol Ophtha 0.1% eye drops 0.3ml unit dose (Spectrum Thea Pharmaceuticals Ltd) |
| 48562 | Ibuprofen 100mg/5ml oral suspension 5ml sachets sugar free |
| 49788 | Voltarol 1% Emulgel (Doncaster Pharmaceuticals Ltd) |
| 25283 | Valenac ec 50mg Gastro-resistant tablet (Shire Pharmaceuticals Ltd) |
| 36650 | Nurofen 200mg tablets (Reckitt Benckiser Healthcare (UK) Ltd) |
| 30806 | Rhumalgan 50mg Tablet (Lagap) |
| 36787 | Nurofen Express 684mg caplets (Reckitt Benckiser Healthcare (UK) Ltd) |
| 1156 | Ibugel 5% gel (Dermal Laboratories Ltd) |
| 10978 | Voltarol 25mg Suppository (Novartis Pharmaceuticals UK Ltd) |
| 56762 | Naproxen 100mg/5ml oral suspension |
| 25718 | Angettes 75 tablets (Bristol-Myers Squibb Pharmaceuticals Ltd) |
| 5200 | Voltarol 50mg suppositories (Novartis Pharmaceuticals UK Ltd) |
| 33321 | Indometacin 50mg capsules (Actavis UK Ltd) |
| 10169 | Brexidol 20mg tablets (Chiesi Ltd) |
| 3972 | Naprosyn EC 250mg tablets (Roche Products Ltd) |
| 18640 | Tolectin 200mg Capsule (Cilag Pharmaceuticals Ltd) |
| 29704 | Paxofen 200mg Tablet (M A Steinhard Ltd) |
| 920 | Indocid 100mg suppositories (Merck Sharp & Dohme Ltd) |
| 40484 | Orudis 100mg capsules (Sanofi) |
| 162 | Arthrotec 50 gastro-resistant tablets (Pfizer Ltd) |
| 10305 | Aspirin 162.5mg capsules |
| 49685 | Aspirin 75mg dispersible tablets (Sigma Pharmaceuticals Plc) |
| 31001 | Cullens headache powders Sachets (Cullen and Davidson) |
| 41364 | Ketoprofen 100mg / Omeprazole 20mg modified-release capsules |
| 1086 | Ibuprofen 600mg tablets |
| 1496 | Indocid R 75mg capsules (Merck Sharp & Dohme Ltd) |
| 29465 | Piroxicam 20mg capsules (Actavis UK Ltd) |
| 46920 | Ketoprofen 200mg Modified-release capsule (Generics (UK) Ltd) |
| 661 | Naproxen 250mg tablets |
| 32036 | Aspirin 75mg dispersible tablets (Actavis UK Ltd) |
| 12122 | Orudis 50mg Capsule (Hawgreen Ltd) |
| 14994 | Clotam Rapid 200mg tablets (Galen Ltd) |
| 22305 | Disprin Extra dispersible tablets (Reckitt Benckiser Healthcare (UK) Ltd) |
| 34386 | Aspirin 300mg tablets (Actavis UK Ltd) |
| 7840 | Oruvail 150mg Modified-release capsule (Hawgreen Ltd) |
| 23204 | Pardelprin MR 75mg capsules (Actavis UK Ltd) |
| 38511 | Feminax Ultra 250mg gastro-resistant tablets (Bayer Plc) |
| 24193 | Imbrilon 25mg Capsule (Berk Pharmaceuticals Ltd) |
| 37648 | Nurofen Express 400mg liquid capsules (Reckitt Benckiser Healthcare (UK) Ltd) |
| 4506 | Volsaid Retard 75 tablets (Chiesi Ltd) |
| 48568 | Boots Rapid Ibuprofen lysine 342mg tablets (The Boots Company Plc) |
| 6115 | Diclofenac sodium 3% gel |
| 8544 | Fenbufen 450mg tablets |
| 7913 | Tiaprofenic acid 200mg tablets |
| 43456 | Anadin LiquiFast 400mg capsules (Pfizer Consumer Healthcare Ltd) |
| 1902 | Aspirn 600mg gastro-resistant tablets |
| 34744 | Diclofenac 100mg Modified-release capsule (Sandoz Ltd) |
| 40215 | Oruvail 100 modified-release capsules (Sanofi) |
| 31945 | Naproxen 500mg Gastro-resistant tablet (Sterwin Medicines) |
| 31944 | Diclofenac sodium 25mg gastro-resistant tablets (Generics (UK) Ltd) |
| 1049 | Nu-seals aspirin 600mg Tablet (Eli Lilly and Company Ltd) |
| 16001 | Ibuprofen 200mg tablets (A A H Pharmaceuticals Ltd) |
| 21770 | Paracetamol 200mg with aspirin 300mg dispersible tablet |
| 37816 | Cuprofen PLUS tablets (SSL International Plc) |
| 55434 | Ibuprofen 400mg tablets (Bristol Laboratories Ltd) |
| 56898 | Rhumalgan SR 75mg capsules (Actavis UK Ltd) |
| 2606 | Ketoprofen 2.5% gel |
| 23932 | Aspro Clear 300mg effervescent tablets (Bayer Plc) |
| 52044 | Aspirin 300mg caplets (The Boots Company Plc) |
| 40756 | Dicloflex 25mg gastro-resistant tablets (Almus Pharmaceuticals Ltd) |
| 259 | Mefenamic acid 250mg capsules |
| 26967 | Alka-Seltzer XS effervescent tablets (Bayer Plc) |
| 51827 | Mefenamic acid 500mg tablets (Sigma Pharmaceuticals Plc) |
| 24617 | Tiloket 2.5% gel (Tillomed Laboratories Ltd) |
| 47501 | Rhumalgan SR 75mg capsules (Almus Pharmaceuticals Ltd) |
| 9044 | Codis 500 dispersible tablets (Reckitt Benckiser Healthcare (UK) Ltd) |
| 58048 | Diclofenac sodium 50mg gastro-resistant tablets (Waymade Healthcare Plc) |
| 474 | Celecoxib 100mg capsules |
| 1755 | Piroxicam 20mg capsules |
| 8969 | Lodine 300mg Capsule (Shire Pharmaceuticals Ltd) |
| 5407 | Naproxen 125mg/5ml oral suspension |
| 13083 | Deep Relief gel (The Mentholatum Company Ltd) |
| 35749 | Radian B Ibuprofen Massage stick (Thornton & Ross Ltd) |
| 53791 | Aspirin 150mg suppositories (Alliance Healthcare (Distribution) Ltd) |
| 18329 | Enprin 75mg gastro-resistant tablets (Galpharm International Ltd) |
| 48218 | Dicloflex sr 100mg Tablet (Teva UK Ltd) |
| 29068 | Nurofen Extra Strength 400mg capsules (Reckitt Benckiser Healthcare (UK) Ltd) |
| 4216 | Brufen 600mg tablets (Abbott Laboratories Ltd) |
| 26216 | Timpron 500mg Tablet (Berk Pharmaceuticals Ltd) |
| 55153 | Nurofen Express Soluble 400mg oral powder sachets (Reckitt Benckiser Healthcare (UK) Ltd) |
| 26970 | Ibuprofen 100mg/5ml oral suspension sugar free (Teva UK Ltd) |
| 2293 | Voltarol 25mg/ml Injection (Novartis Pharmaceuticals UK Ltd) |
| 51237 | Voltarol 1% Emulgel (Waymade Healthcare Plc) |
| 52617 | Ibuprofen 100mg/5ml oral suspension sugar free (Sigma Pharmaceuticals Plc) |
| 1051 | Indometacin 75mg modified-release tablets |
| 34743 | Naproxen 500mg gastro-resistant tablets (A A H Pharmaceuticals Ltd) |
| 33645 | Diclofenac 75mg Modified-release tablet (IVAX Pharmaceuticals UK Ltd) |
| 1984 | Diclofenac sodium 100mg modified-release tablets |
| 31383 | Dexomon 75mg SR tablets (Hillcross Pharmaceuticals Ltd) |
| 3421 | Diclomax sr 75mg Modified-release capsule (Provalis Healthcare Ltd) |
| 44313 | Indoflex 25mg Capsule (Unimed Pharmaceuticals Ltd) |
| 4679 | Asasantin Retard capsules (Boehringer Ingelheim Ltd) |
| 57112 | Ibuprofen 400mg tablets (Alliance Healthcare (Distribution) Ltd) |
| 39823 | Dicloflex 50mg gastro-resistant tablets (Almus Pharmaceuticals Ltd) |
| 33559 | Diclofenac 50mg Tablet (C P Pharmaceuticals Ltd) |
| 29352 | Ibuprofen 100mg/5ml oral suspension sugar free (Vantage) |
| 41367 | Ketoprofen 200mg / Omeprazole 20mg modified-release capsules |
| 34362 | Diclofenac 25mg Gastro-resistant tablet (Genus Pharmaceuticals Ltd) |
| 18662 | Indomod 75mg modified-release capsules (Pfizer Ltd) |
| 4911 | Ibuprofen 400mg Granules |
| 45840 | Aspirin 300mg Dispersible tablet (Numark Management Ltd) |
| 3710 | Piroxicam 20mg dispersible tablets |
| 12075 | Mobiflex 20mg Tablet (Roche Products Ltd) |
| 849 | Ibumed 400mg Tablet (Medipharma Ltd) |
| 15363 | Nurofen Cold and Flu tablets (Reckitt Benckiser Healthcare (UK) Ltd) |
| 10149 | Ibuprofen 200mg capsules |
| 27778 | Solpaflex 2.50% Gel (GlaxoSmithKline Consumer Healthcare) |
| 34385 | Aspirin 75mg Soluble tablet (Co-operative) |
| 50343 | Feldene 0.5% gel (Doncaster Pharmaceuticals Ltd) |
| 33180 | Ketoprofen cr 200mg Capsule (Bristol-Myers Squibb Pharmaceuticals Ltd) |
| 20384 | Flamatak MR 100mg tablets (Actavis UK Ltd) |
| 31950 | Diclofenac sodium 50mg gastro-resistant tablets (Sterwin Medicines) |
| 54783 | Naproxen 250mg tablets (Teva UK Ltd) |
| 3409 | Feldene 20mg Orodispersible tablet (Pfizer Ltd) |
| 6696 | Micropirin 75mg gastro-resistant tablets (Dexcel-Pharma Ltd) |
| 15180 | Naproxen and misoprostol 500mgwith200microgram combined Tablet |
| 31787 | Econac SR 75mg tablets (Mercury Pharma Group Ltd) |
| 46342 | Medifen 3with months 100mg/5ml Oral suspension (SSL International Plc) |
| 55486 | Naproxen 500mg tablets (Teva UK Ltd) |
| 1446 | Voltarol 50mg Tablet (Novartis Pharmaceuticals UK Ltd) |
| 3597 | Nurofen 200mg Soluble tablet (Crookes Healthcare Ltd) |
| 7539 | Beechams Powders oral powder sachets (SmithKline Beecham Plc) |
| 40185 | Oruvail 200 modified-release capsules (Sanofi) |
| 4762 | Ibuleve Sports 5% gel (Dendron Ltd) |
| 50059 | Celebrex 100mg capsules (Necessity Supplies Ltd) |
| 32992 | Aspirin 75mg gastro-resistant tablets (Generics (UK) Ltd) |
| 22283 | Lemsip flu 12 hr Modified-release capsule (Reckitt Benckiser Healthcare (UK) Ltd) |
| 55913 | Voltarol 50mg suppositories (Lexon (UK) Ltd) |
| 24025 | Caprin 300mg gastro-resistant tablets (Pinewood Healthcare) |
| 784 | Ibuprofen 300mg modified-release capsules |
| 14707 | Defanac Retard 100mg tablets (Ranbaxy (UK) Ltd) |
| 53617 | Ibuprofen and codeine 200mg+12.8mg Tablet (Almus Pharmaceuticals Ltd) |
| 37688 | Diclofenac sodium 1% gel |
| 31064 | Mobiflex 20mg Granules (Roche Products Ltd) |
| 16637 | Ketorolac 10mg tablets |
| 44730 | Mentholatum Ibuprofen 5% gel (The Mentholatum Company Ltd) |
| 31959 | Indometacin 50mg capsules (A A H Pharmaceuticals Ltd) |
| 597 | Diclofenac potassium 50mg tablets |
| 41512 | Aspirin 75mg gastro-resistant tablets (Teva UK Ltd) |
| 44112 | Voltarol Joint Pain 12.5mg tablets (Novartis Consumer Health UK Ltd) |
| 11999 | Orudis 100mg Capsule (Hawgreen Ltd) |
| 5812 | Etoricoxib 90mg tablets |
| 34 | Aspirin 75mg gastro-resistant tablets |
| 53178 | Aspirin 75mg gastro-resistant tablets (Wockhardt UK Ltd) |
| 5938 | Etoricoxib 120mg tablets |
| 18217 | Aspirin 300mg orodispersible tablets sugar free |
| 55894 | Naproxen 500mg gastro-resistant tablets (Phoenix Healthcare Distribution Ltd) |
| 52141 | Mobilan 25mg Capsule (Galen Ltd) |
| 11540 | Diclofenac 16mg/ml topical solution |
| 650 | Etoricoxib 60mg tablets |
| 54870 | Piroxicam 0.5% gel (Alliance Healthcare (Distribution) Ltd) |
| 50117 | Brufen 100mg/5ml syrup (Lexon (UK) Ltd) |
| 20907 | Sudafed Sinus Pressure & Pain tablets (McNeil Products Ltd) |
| 5401 | Voltarol Rapid 25mg tablets (Novartis Pharmaceuticals UK Ltd) |
| 47401 | Ibuprofen 5% gel (Galpharm International Ltd) |
| 29330 | Diclofenac sodium 50mg gastro-resistant tablets (Sandoz Ltd) |
| 9144 | Caprin 75mg gastro-resistant tablets (Wockhardt UK Ltd) |
| 55230 | Aspirin 300mg dispersible tablets (Kent Pharmaceuticals Ltd) |
| 48546 | Ibuprofen 400mg caplets (Bristol Laboratories Ltd) |
| 31777 | Piroxicam 20mg dispersible tablets (Generics (UK) Ltd) |
| 5080 | Celebrex 200mg capsules (Pfizer Ltd) |
| 33113 | Artracin 50mg Capsule (DDSA Pharmaceuticals Ltd) |
| 14776 | Surgam 300mg tablets (Sanofi) |
| 12607 | KETOROLAC TROMETAMOL 30 MG/ML INJ |
| 10589 | Fenopron 600 tablets (Typharm Ltd) |
| 34212 | Diclofenac 75mg Modified-release tablet (Genus Pharmaceuticals Ltd) |
| 26522 | Meflam 500mg Tablet (Trinity Pharmaceuticals Ltd) |
| 56554 | Naproxen 250mg/5ml oral suspension |
| 30923 | Diclofenac 100mg suppositories (A A H Pharmaceuticals Ltd) |
| 27782 | Ibuprofen 400mg tablets (Teva UK Ltd) |
| 2197 | Naproxen 375mg Tablet |
| 28168 | Nurofen Recovery 200mg orodispersible tablets (Reckitt Benckiser Healthcare (UK) Ltd) |
| 37805 | Polyurethane foam Film dressing 15cmx15cm |
| 50555 | Aspirin 300mg dispersible tablets (Doncaster Pharmaceuticals Ltd) |
| 28900 | Indometacin 25mg Capsule (Generics (UK) Ltd) |
| 53331 | Ibuprofen 100mg/5ml oral suspension sugar free (Alliance Healthcare (Distribution) Ltd) |
| 50317 | Voltarol 75mg SR tablets (Lexon (UK) Ltd) |
| 48161 | Naproxen 500mg Tablet (Almus Pharmaceuticals Ltd) |
| 22138 | Aspirin 324mg modified-release tablets |
| 34447 | Ibuprofen 200mg tablets (Thornton & Ross Ltd) |
| 24007 | Valrox 500mg Tablet (Shire Pharmaceuticals Ltd) |
| 6666 | Dipyridamole 200mg modified-release / Aspirin 25mg capsules |
| 41569 | Aspirin 300mg tablets (A A H Pharmaceuticals Ltd) |
| 31858 | Caspac xl 162.5mg Capsule (Pharmacia Ltd) |
| 31210 | Aspirin 300mg Tablet (Co-operative) |
| 52229 | Voltarol 1% Emulgel (Sigma Pharmaceuticals Plc) |
| 6881 | Solaraze 3% gel (Almirall Ltd) |
| 57045 | Voltarol 50mg dispersible tablets (Waymade Healthcare Plc) |
| 1739 | Brufen 400mg tablets (Abbott Laboratories Ltd) |
| 177 | Indometacin 25mg capsules |
| 1231 | Ketoprofen 100mg capsules |
| 7424 | Fenbufen 300mg capsules |
| 39019 | Brufen Retard 800mg tablets (Abbott Laboratories Ltd) |
| 33994 | Diclofenac sodium 25mg gastro-resistant tablets (IVAX Pharmaceuticals UK Ltd) |
| 34910 | Mefenamic acid 500mg Tablet (Berk Pharmaceuticals Ltd) |
| 17030 | Rhumalgan SR 75mg capsules (Sandoz Ltd) |
| 51339 | Indometacin 25mg capsules (Genesis Pharmaceuticals Ltd) |
| 17029 | Rhumalgan CR 75 tablets (Sandoz Ltd) |
| 1233 | Diclofenac sodium 75mg modified-release tablets |
| 8645 | Aspirin 300mg effervescent tablets |
| 57370 | Meloxicam 15mg orodispersible tablets sugar free |
| 42821 | Nabumetone 500mg tablets (A A H Pharmaceuticals Ltd) |
| 1983 | Mefenamic acid 250mg Dispersible tablet |
| 42397 | Nurofen Express 256mg tablets (Reckitt Benckiser Healthcare (UK) Ltd) |
| 1246 | Ponstan 250mg Dispersible tablet (Chemidex Pharma Ltd) |
| 15005 | Indomod 25mg modified-release capsules (Pfizer Ltd) |
| 53345 | Voltarol Rapid 50mg tablets (Lexon (UK) Ltd) |
| 34485 | Aspirin 75mg gastro-resistant tablets (IVAX Pharmaceuticals UK Ltd) |
| 586 | Ibuprofen 200mg Capsule |
| 41366 | Axorid 100mg/20mg modified-release capsules (Meda Pharmaceuticals Ltd) |
| 3817 | Synflex 275mg tablets (Roche Products Ltd) |
| 26351 | Rheumatac Retard 75 tablets (Amdipharm Plc) |
| 13818 | Nabumetone 500mg tablets (Actavis UK Ltd) |
| 4648 | Ibuspray 5% spray (Dermal Laboratories Ltd) |
| 14422 | Fenbufen 450mg Effervescent tablet |
| 41621 | Piroxicam 20mg capsules (A A H Pharmaceuticals Ltd) |
| 2257 | Surgam SA 300mg capsules (Sanofi) |
| 8062 | Motifene 75mg modified-release capsules (Daiichi Sankyo UK Ltd) |
| 32854 | Diclofenac sodium 75mg modified-release capsules (A A H Pharmaceuticals Ltd) |
| 51874 | Arcoxia 30mg tablets (Lexon (UK) Ltd) |
| 48871 | Diclofenac potassium 25mg tablets (Actavis UK Ltd) |
| 3897 | Sulindac 100mg tablets |
| 3334 | Ketorolac 0.5% eye drops |
| 26242 | Timpron 250mg Tablet (Berk Pharmaceuticals Ltd) |
| 52154 | Ibuprofen 200mg tablets (Galpharm International Ltd) |
| 23026 | Artracin sr 75mg Modified-release capsule (Trinity Pharmaceuticals Ltd) |
| 645 | Aspirin 300mg suppositories |
| 32090 | Mefenamic acid 500mg tablets (Actavis UK Ltd) |
| 10209 | Ibufem 200mg tablets (Galpharm International Ltd) |
| 37850 | Ibucalm Ibuprofen Pain Relief 5% gel (Aspar Pharmaceuticals Ltd) |
| 55233 | Ibuprofen 400mg Tablet (Nucare Plc) |
| 1073 | Mefenamic acid 500mg tablets |
| 45814 | First Resort Double Action Pain Relief 12.5mg tablets (Actavis UK Ltd) |
| 4469 | Fenoprofen 300mg tablets |
| 19382 | Slofenac 75mg SR tablets (Sterwin Medicines) |
| 499 | Diclofenac 50mg suppositories |
| 11970 | Meloxicam 7.5mg suppositories |
| 13639 | Flexin-50 Continus tablets (Napp Pharmaceuticals Ltd) |
| 44800 | Naproxen 500mg / Esomeprazole 20mg modified-release tablets |
| 17680 | Indomax 75 SR capsules (Ashbourne Pharmaceuticals Ltd) |
| 24137 | Indometacin 25mg capsules (Actavis UK Ltd) |
| 36329 | Ibuprofen 10% gel (Thornton & Ross Ltd) |
| 13627 | Mobic 15mg suppositories (Boehringer Ingelheim Ltd) |
| 11168 | Volsaid Retard 100 tablets (Chiesi Ltd) |
| 4045 | Naprosyn EC 375mg tablets (Roche Products Ltd) |
| 12776 | Ibumousse 5% (Dermal Laboratories Ltd) |
| 11215 | Voltarol 25mg suppositories (Novartis Pharmaceuticals UK Ltd) |
| 26247 | Opustan 500mg Tablet (Opus Pharmaceuticals Ltd) |
| 22230 | Meflam 250mg Capsule (Trinity Pharmaceuticals Ltd) |
| 40516 | Anadin LiquiFast 200mg capsules (Pfizer Consumer Healthcare Ltd) |
| 14517 | Robaxisal forte Tablet (Shire Pharmaceuticals Ltd) |
| 52618 | Aspirin 75mg dispersible tablets (Bristol Laboratories Ltd) |
| 33785 | Galprofen 200mg tablets (Galpharm International Ltd) |
| 32875 | Ibuprofen 400mg tablets (Sandoz Ltd) |
| 30942 | Diclofenac 50mg Tablet (Regent Laboratories Ltd) |
| 31916 | Tiloket CR 100mg capsules (Tillomed Laboratories Ltd) |
| 33293 | Aspirin 75mg gastro-resistant tablets (Sterwin Medicines) |
| 54906 | Diclofenac 50mg/5ml oral suspension |
| 56213 | Ibuprofen 400mg tablets sugar coated (Kent Pharmaceuticals Ltd) |
| 10265 | Fenbid 5% gel (Mercury Pharma Group Ltd) |
| 48644 | Ibuprofen 400mg caplets (Lloyds Pharmacy Ltd) |
| 6249 | Froben 100mg tablets (Abbott Laboratories Ltd) |
| 332 | Ibuprofen 5% gel |
| 55505 | Naproxen 250mg gastro-resistant tablets (Kent Pharmaceuticals Ltd) |
| 8401 | Motrin 400mg tablets (Pfizer Ltd) |
| 41624 | Piroxicam 10mg capsules (IVAX Pharmaceuticals UK Ltd) |
| 1115 | Diclofenac sodium 100mg modified-release capsules |
| 12992 | Aspirin 500mg / Codeine 8mg dispersible tablets sugar free |
| 23425 | Nurofen Migraine Pain 342mg tablets (Reckitt Benckiser Healthcare (UK) Ltd) |
| 25342 | Arthrosin EC 500 tablets (Ashbourne Pharmaceuticals Ltd) |
| 32210 | Aspirin 300mg dispersible tablets (Actavis UK Ltd) |
| 54760 | Parecoxib 40mg powder and solvent for solution for injection vials |
| 20059 | Tiaprofenic acid 300mg sachets |
| 18647 | Fenoket 200mg modified-release capsules (Opus Pharmaceuticals Ltd) |
| 32097 | Indometacin 75mg Modified-release capsule (Actavis UK Ltd) |
| 34289 | Naproxen 250mg gastro-resistant tablets (Generics (UK) Ltd) |
| 28348 | Ibuprofen 200mg tablets (Teva UK Ltd) |
| 49432 | Calprofen 100mg/5ml oral suspension (McNeil Products Ltd) |
| 10558 | Flexin-75 Continus tablets (Napp Pharmaceuticals Ltd) |
| 9474 | Preservex 100mg tablets (Almirall Ltd) |
| 32862 | Ibuprofen 100mg/5ml oral suspension sugar free (Thornton & Ross Ltd) |
| 17201 | Motrin 600mg tablets (Pfizer Ltd) |
| 35882 | Diclofenac 0.1% eye drops 0.3ml unit dose preservative free |
| 2827 | Feldene 10mg dispersible tablets (Pfizer Ltd) |
| 21380 | Aspirin 75mg / Isosorbide mononitrate 60mg modified-release tablets |
| 16170 | Fenbufen 300mg capsules (Genus Pharmaceuticals Ltd) |
| 48071 | Piroxicam 0.50% Gel (Manx Pharma Ltd) |
| 25341 | Arthrosin EC 250 tablets (Ashbourne Pharmaceuticals Ltd) |
| 57943 | Valket 200 Retard capsules (Tillomed Laboratories Ltd) |
| 2258 | Emflex 60mg capsules (Merck Serono Ltd) |
| 2628 | Nu-seals aspirin ec 75mg Gastro-resistant tablet (Eli Lilly and Company Ltd) |
| 31499 | Paracetamol with aspirin tablet |
| 21949 | Toradol 30mg/1ml solution for injection ampoules (Roche Products Ltd) |
| 34911 | Ibuprofen 200mg Tablet (Celltech Pharma Europe Ltd) |
| 14476 | Indolar SR 75mg capsules (Sandoz Ltd) |
| 38770 | Lodine SR 600mg tablets (Almirall Ltd) |
| 1470 | Mobic 15mg tablets (Boehringer Ingelheim Ltd) |
| 3599 | Ibuprofen 600mg effervescent granules sachets |
| 2288 | Naprosyn 250mg tablets (Roche Products Ltd) |
| 51808 | Diclofenac 12.5mg/5ml oral solution |
| 34309 | Aspirin 300mg dispersible tablets (A A H Pharmaceuticals Ltd) |
| 3852 | Diclomax 100mg Modified-release capsule (Provalis Healthcare Ltd) |
| 3266 | Flurbiprofen 50mg tablets |
| 2463 | Piroxicam 10mg dispersible tablets |
| 30920 | Aspirin 300mg Dispersible tablet (M & A Pharmachem Ltd) |
| 37562 | Arcoxia 30mg tablets (Merck Sharp & Dohme Ltd) |
| 57057 | Aspirin 75mg dispersible tablets (Wockhardt UK Ltd) |
| 42218 | Co-codaprin 8mg/400mg dispersible tablets (A A H Pharmaceuticals Ltd) |
| 30297 | Diclofenac 50mg Gastro-resistant tablet (Pharmacia Ltd) |
| 32136 | Ibular 200mg Tablet (Lagap) |
| 34354 | Ibuprofen 200mg tablets (Vantage) |
| 39876 | Mobigel 4% spray (Mercury Pharma Group Ltd) |
| 30724 | Galprofen 100mg/5ml oral suspension (Galpharm International Ltd) |
| 14570 | Ibuleve 5% Mousse (Dendron Ltd) |
| 4095 | Voltarol 12.5mg Suppository (Novartis Pharmaceuticals UK Ltd) |
| 52931 | Naproxen 500mg gastro-resistant tablets (Kent Pharmaceuticals Ltd) |
| 33668 | Aspirin 300mg Dispersible tablet (Rusco Ltd) |
| 54997 | Aspirin 75mg dispersible tablets (Dowelhurst Ltd) |
| 32227 | Larafen CR 200mg capsules (Ennogen Pharma Ltd) |
| 38817 | Diclofenac potassium 12.5mg tablets |
| 57475 | Meloxicam 7.5mg orodispersible tablets sugar free |
| 54476 | Naproxen 500mg gastro-resistant tablets (Genesis Pharmaceuticals Ltd) |
| 24121 | Diclofenac sodium 25mg gastro-resistant tablets (Actavis UK Ltd) |
| 3311 | Etodolac 200mg capsules |
| 34487 | Diclofenac sodium 50mg gastro-resistant tablets (IVAX Pharmaceuticals UK Ltd) |
| 53711 | Aspirin 300mg Tablet (Nucare Plc) |
| 37972 | Ibuleve Speed Relief 5% gel (Dendron Ltd) |
| 39708 | Diclofenac 4% cutaneous spray |
| 17126 | Fenactol SR 75mg tablets (Discovery Pharmaceuticals Ltd) |
| 1116 | Diclofenac 100mg suppositories |
| 8600 | Piroxicam 20mg suppositories |
| 25619 | Nurofen 400mg Tablet (Crookes Healthcare Ltd) |
| 10625 | Indocid 5mg/ml oral suspension (Merck Sharp & Dohme Ltd) |
| 570 | Dynastat 40mg Powder for solution for injection (Pharmacia Ltd) |
| 52338 | Diclofenac potassium 50mg tablets (Focus Pharmaceuticals Ltd) |
| 50949 | Aspirin 75mg tablets (A A H Pharmaceuticals Ltd) |
| 47350 | Voltarol Active 4% spray (Novartis Consumer Health UK Ltd) |
| 1708 | Codafen Continus tablets (Napp Pharmaceuticals Ltd) |
| 56078 | Rhumalgan XL 100mg capsules (Almus Pharmaceuticals Ltd) |
| 21824 | Flamrase 50 EC tablets (Teva UK Ltd) |
| 25205 | Ibuprofen 100mg/5ml oral suspension 5ml sachets sugar free (Thornton & Ross Ltd) |
| 48000 | Aspirin 300mg tablets (Sigma Pharmaceuticals Plc) |
| 21813 | Lidifen 400mg Tablet (Berk Pharmaceuticals Ltd) |
| 21955 | Ketozip 200 XL capsules (Ashbourne Pharmaceuticals Ltd) |
| 35292 | Nurofen 200mg liquid capsules (Reckitt Benckiser Healthcare (UK) Ltd) |
| 2200 | Indometacin 25mg modified-release capsules |
| 28553 | Diclofenac sodium 50mg gastro-resistant tablets (Teva UK Ltd) |
| 23841 | Safapryn Tablet (Pfizer Ltd) |
| 43679 | Flamasacard 162.5mg Modified-release capsule (Abbey Pharmaceuticals Ltd) |
| 42455 | Dicloflex Retard 100mg tablets (Teva UK Ltd) |
| 33676 | Aspirin 75mg dispersible tablets (Kent Pharmaceuticals Ltd) |
| 51343 | Voltarol Rapid 25mg tablets (Doncaster Pharmaceuticals Ltd) |
| 29332 | Ibuprofen 100mg/5ml oral suspension sugar free (Sandoz Ltd) |
| 11495 | Piroxicam betadex 20mg tablets |
| 16193 | Motrin 800mg tablets (Pfizer Ltd) |
| 17128 | Fenactol 50mg gastro-resistant tablets (Discovery Pharmaceuticals Ltd) |
| 3335 | Acular 0.5% eye drops (Allergan Ltd) |
| 45213 | Diclofenac 10mg dispersible tablets |
| 580 | Diclofenac sodium 75mg modified-release tablets |
| 18921 | Fenactol 25mg gastro-resistant tablets (Discovery Pharmaceuticals Ltd) |
| 20466 | Voltarol Ophtha Multidose 0.1% eye drops (Spectrum Thea Pharmaceuticals Ltd) |
| 3170 | Meloxicam 15mg suppositories |
| 45262 | Naproxen Oral solution |
| 19575 | Proflex 200mg Tablet (Novartis Consumer Health UK Ltd) |
| 8185 | Disprin CV 300mg modified-release tablets (Reckitt Benckiser Healthcare (UK) Ltd) |
| 29054 | Methocarbamol with aspirin Tablet |
| 48675 | Ibuprofen Pain Relief Maximum Strength 10% gel (Numark Management Ltd) |
| 28390 | Valenac ec 25mg Gastro-resistant tablet (Shire Pharmaceuticals Ltd) |
| 34850 | Ibuprofen 600mg tablets (Teva UK Ltd) |
| 51828 | Ibuprofen 100mg/5ml oral suspension sugar free (Kent Pharmaceuticals Ltd) |
| 49799 | Aspirin 150mg suppositories (A A H Pharmaceuticals Ltd) |
| 55454 | Naproxen 500mg tablets (Kent Pharmaceuticals Ltd) |
| 10481 | Lederfen f 450mg Tablet (Wyeth Pharmaceuticals) |
| 56925 | Naproxen 250mg tablets (Actavis UK Ltd) |
| 18371 | Digenac xl 100mg Modified-release tablet (Genus Pharmaceuticals Ltd) |
| 34621 | Ibuprofen 200mg Tablet (Nucare Plc) |
| 37235 | Ibuprofen 100mg/5ml / Pseudoephedrine 15mg/5ml oral suspension sugar free |
| 49059 | Voltarol 50mg dispersible tablets (Lexon (UK) Ltd) |
| 30282 | Diclofenac 75mg Modified-release tablet (Galen Ltd) |
| 49060 | Aspirin 75mg dispersible tablets (Alliance Healthcare (Distribution) Ltd) |
| 27200 | Diclovol Retard 100mg tablets (Generics (UK) Ltd) |
| 2243 | Meloxicam 7.5mg tablets |
| 34143 | Naprosyn 375 Tablet (Roche Products Ltd) |
| 9630 | Feldene P 0.5% gel (Pfizer Ltd) |
| 47816 | Tenoxicam 20mg Tablet (Sovereign Medical Ltd) |
| 1043 | Naproxen sodium 275mg tablets |
| 1137 | Nu-seals aspirin ec 300mg Gastro-resistant tablet (Eli Lilly and Company Ltd) |
| 10792 | Voltarol 50mg Suppository (Novartis Pharmaceuticals UK Ltd) |
| 10939 | Toradol 10mg/1ml solution for injection ampoules (Roche Products Ltd) |
| 25362 | Defanac 25mg gastro-resistant tablets (Ranbaxy (UK) Ltd) |
| 14251 | Ketorolac 30mg/1ml solution for injection ampoules |
| 54734 | Aspirin 300mg tablets (Wockhardt UK Ltd) |
| 49277 | Ibuprofen 200mg caplets (Bristol Laboratories Ltd) |
| 1210 | Indometacin 75mg modified-release capsules |
| 36606 | Manorfen 400mg tablets (The Manor Drug Company (Nottingham) Ltd) |
| 41823 | Indometacin sr 75mg Modified-release capsule (Generics (UK) Ltd) |
| 56282 | Diclofenac 2% gel |
| 157 | Voltarol 100mg Suppository (Novartis Pharmaceuticals UK Ltd) |
| 1096 | Diclofenac sodium 25mg gastro-resistant tablets |
| 40664 | Oruvail 150 modified-release capsules (Sanofi) |
| 838 | Oruvail 200mg Modified-release capsule (Hawgreen Ltd) |
| 1075 | Diclofenac sodium 50mg gastro-resistant tablets |
| 16272 | Lofensaid Retard 100 tablets (Opus Pharmaceuticals Ltd) |
| 34942 | Aspirin 75mg Dispersible tablet (Nucare Plc) |
| 9201 | Ibuleve 5% spray (Dendron Ltd) |
| 33589 | Ibuprofen 400mg tablets (Thornton & Ross Ltd) |
| 46919 | Ketoprofen sr 200mg Capsule (Approved Prescription Services Ltd) |
| 16611 | Anadin Tablet (Wyeth Consumer Healthcare) |
| 50269 | Arthrotec 75 gastro-resistant tablets (Mawdsley-Brooks & Company Ltd) |
| 32704 | Advil cold and sinus 200mg+30mg Tablet (Wyeth Consumer Healthcare) |
| 39502 | Ibuprofen sodium dihydrate 200mg tablets |
| 42406 | Diclofenac 50mg Gastro-resistant tablet (Almus Pharmaceuticals Ltd) |
| 43096 | Ibuleve Speed Relief 5% spray (Dendron Ltd) |
| 25790 | Rhumalgan 25mg Tablet (Lagap) |
| 18030 | Imazin XL forte tablets (Napp Pharmaceuticals Ltd) |
| 33111 | Prosaid 250mg Tablet (BHR Pharmaceuticals Ltd) |
| 2105 | Solprin 300mg Tablet (Reckitt Benckiser Healthcare (UK) Ltd) |
| 16194 | Lodine 200mg Tablet (Shire Pharmaceuticals Ltd) |
| 28332 | Mobiflex 20mg powder and solvent for solution for injection vials (Roche Products Ltd) |
| 2938 | Ibuprofen 100mg/5ml Oral suspension |
| 7222 | Tolfenamic acid 200mg tablets |
| 389 | Ketoprofen 50mg capsules |
| 37253 | Anadin ultra double strength 400mg Capsule (Wyeth Consumer Healthcare) |
| 56106 | Naproxen 500mg/5ml oral suspension |
| 58112 | Alka-Seltzer effervescent tablets original (Bayer Plc) |
| 1778 | Surgam 300mg Tablet (Sanofi) |
| 14901 | Diclofenac 1% transdermal patches |
| 24212 | Imbrilon 50mg Capsule (Berk Pharmaceuticals Ltd) |
| 29587 | Ebufac 400mg Tablet (DDSA Pharmaceuticals Ltd) |
| 11466 | Nabumetone 500mg/5ml oral suspension sugar free |
| 685 | Aspav dispersible tablets (Actavis UK Ltd) |
| 21811 | Lidifen 200mg Tablet (Berk Pharmaceuticals Ltd) |
| 24960 | Aspirin 300mg tablets (Vantage) |
| 31953 | Aspirin 75mg dispersible tablets (IVAX Pharmaceuticals UK Ltd) |
| 40144 | Aspirin 300mg Dispersible tablet (Thornton & Ross Ltd) |
| 36486 | Econac XL 100mg tablets (Mercury Pharma Group Ltd) |
| 48084 | Ibuprofen 200mg/5ml oral suspension |
| 15068 | Arthrofen 400 tablets (Ashbourne Pharmaceuticals Ltd) |
| 43911 | Ibuprofen 600mg Tablet (C P Pharmaceuticals Ltd) |
| 4692 | Dicloflex 50mg gastro-resistant tablets (Dexcel-Pharma Ltd) |
| 46440 | Naproxen 500mg Tablet (M & A Pharmachem Ltd) |
| 54353 | Generic Anadin Extra soluble tablets sugar free |
| 51099 | Voltarol Rapid 50mg tablets (Mawdsley-Brooks & Company Ltd) |
| 21421 | Seractil 400mg tablets (Genus Pharmaceuticals Ltd) |
| 5767 | Ibuprofen 10% gel |
| 24128 | Diclofenac sodium 25mg gastro-resistant tablets (A A H Pharmaceuticals Ltd) |
| 141 | Piroxicam 10mg capsules |
| 26214 | Fenbuzip 450mg Tablet (Ashbourne Pharmaceuticals Ltd) |
| 8789 | Dicloflex retard tabs 100 100mg Modified-release tablet (Dexcel-Pharma Ltd) |
| 6208 | Voltarol 1% Emulgel P (Novartis Consumer Health UK Ltd) |
| 21807 | Flamrase 25 EC tablets (Teva UK Ltd) |
| 45320 | Ibuprofen 200mg tablets (Sandoz Ltd) |
| 14380 | Lederfen 300mg capsules (Mercury Pharma Group Ltd) |
| 26404 | Tolmetin 200mg Capsule |
| 2904 | Diclofenac sodium 75mg gastro-resistant modified-release capsules |
| 1621 | Brufen 200mg tablets (Abbott Laboratories Ltd) |
| 54514 | Ibuprofen lysine 400mg oral powder sachets |
| 8385 | Ketoprofen 150mg modified-release capsules |
| 34762 | Aspirin 300mg Gastro-resistant tablet (Galen Ltd) |
| 4710 | Mefenamic acid 250mg Capsule (Actavis UK Ltd) |
| 4564 | Fenoprofen 200mg Tablet |
| 21444 | Volraman 25mg gastro-resistant tablets (LPC Medical (UK) Ltd) |
| 39738 | Aspirin 162.5mg modified-release capsules |
| 24236 | Slofenac 100mg Modified-release tablet (Sterwin Medicines) |
| 9939 | Aspirin 500mg effervescent tablets sugar free |
| 46860 | Anadin LiquiFast 200mg effervescent tablets (Pfizer Consumer Healthcare Ltd) |
| 11951 | Original Phensic Aspirin tablets (Merck Consumer Health Products) |
| 19036 | Arthrofen 200 tablets (Ashbourne Pharmaceuticals Ltd) |
| 47820 | Voltarol Pain-eze Extra Strength 25mg tablets (Novartis Consumer Health UK Ltd) |
| 19320 | Piroflam 20mg Capsule (Opus Pharmaceuticals Ltd) |
| 17754 | Progesic 200mg Tablet (Eli Lilly and Company Ltd) |
| 11995 | Orudis 100mg Suppository (Hawgreen Ltd) |
| 28810 | Aspirin 300mg with Glycine 133mg soluble tablets |
| 34924 | Mefenamic acid 250mg Capsule (Teva UK Ltd) |
| 43806 | Aspirin 300mg gastro-resistant tablets (Sandoz Ltd) |
| 497 | Voltarol 25mg gastro-resistant tablets (Novartis Pharmaceuticals UK Ltd) |
| 18448 | Voltarol 12.5mg suppositories (Novartis Pharmaceuticals UK Ltd) |
| 54518 | Diclofenac sodium 50mg gastro-resistant tablets (Phoenix Healthcare Distribution Ltd) |
| 32366 | Relcofen 200mg Tablet (Actavis UK Ltd) |
| 31178 | Ketoprofen 2.5% gel (A A H Pharmaceuticals Ltd) |
| 20442 | Nurofen 5% gel (Reckitt Benckiser Healthcare (UK) Ltd) |
| 21864 | Pirozip 10 capsules (Ashbourne Pharmaceuticals Ltd) |
| 55579 | Aspirin 300mg tablets (Almus Pharmaceuticals Ltd) |
| 22618 | Solprin 75mg Tablet (Reckitt Benckiser Healthcare (UK) Ltd) |
| 3432 | Naproxen 375mg gastro-resistant tablets |
| 4043 | Froben sr 200mg Modified-release capsule (Abbott Laboratories Ltd) |
| 26159 | Fenbid Forte 10% gel (Mercury Pharma Group Ltd) |
| 37502 | Ibuprofen 10mg/2ml solution for infusion ampoules |
| 43616 | Celecoxib 400mg capsules |
| 9500 | Diclotard 75mg modified-release tablets (Galen Ltd) |
| 26888 | Difenor xl 100mg Modified-release tablet (IVAX Pharmaceuticals UK Ltd) |
| 15367 | Anadin Extra tablets (Pfizer Consumer Healthcare Ltd) |
| 16192 | Motrin 200mg Tablet (Pharmacia Ltd) |
| 48810 | Dysman 250 capsules (Ashbourne Pharmaceuticals Ltd) |
| 7522 | Lederfen 300mg Tablet (Wyeth Pharmaceuticals) |
| 30243 | Ibuprofen 200mg effervescent tablets |
| 52956 | Voltarol 1% Emulgel (Stephar (U.K.) Ltd) |
| 56651 | Piroxicam 0.5% gel (Sigma Pharmaceuticals Plc) |
| 46968 | Mefenamic acid 250mg capsules (Generics (UK) Ltd) |
| 53384 | Voltarol 50mg dispersible tablets (Mawdsley-Brooks & Company Ltd) |
| 34610 | Naproxen 500mg gastro-resistant tablets (Generics (UK) Ltd) |
| 26994 | Fenbuzip 300mg Tablet (Ashbourne Pharmaceuticals Ltd) |
| 33704 | Ibuprofen 100mg/5ml oral suspension sugar free (A A H Pharmaceuticals Ltd) |
| 25358 | Defanac 50mg gastro-resistant tablets (Ranbaxy (UK) Ltd) |
| 402 | Nurofen 200mg Tablet (Crookes Healthcare Ltd) |
| 29345 | Ibuprofen 100mg/5ml Oral suspension (Hillcross Pharmaceuticals Ltd) |
| 344 | Acemetacin 60mg capsules |
| 676 | Diclofenac 75mg/3ml solution for injection ampoules |
| 34611 | Aspirin 75mg gastro-resistant tablets (C P Pharmaceuticals Ltd) |
| 34663 | Ibuprofen 100mg/5ml Oral suspension (Neo Laboratories Ltd) |
| 42500 | Ketoprofen sr 100mg Capsule (Approved Prescription Services Ltd) |
| 31589 | Diclofenac sodium 75mg modified-release tablets (A A H Pharmaceuticals Ltd) |
| 8663 | Naprosyn S/R 500mg tablets (Roche Products Ltd) |
| 38948 | Diclomax Retard 100mg capsules (Galen Ltd) |
| 44892 | Sudafed sinus pressure & pain Tablet (McNeil Products Ltd) |
| 54430 | Aspirin 75mg tablets (Alliance Healthcare (Distribution) Ltd) |
| 48021 | Aspirin 75mg Tablet (Hillcross Pharmaceuticals Ltd) |
| 41450 | Orudis 100mg suppositories (Sanofi) |
| 11322 | Flamrase sr 75mg Modified-release tablet (APS Berk) |
| 52009 | Ibuprofen 200mg capsules (Galpharm International Ltd) |
| 20386 | Ramodar 200mg Tablet (Wyeth Pharmaceuticals) |
| 850 | Mobic 7.5mg tablets (Boehringer Ingelheim Ltd) |
| 33318 | Indometacin 50mg Capsule (Generics (UK) Ltd) |
| 41817 | Indometacin sr 75mg Modified-release capsule (C P Pharmaceuticals Ltd) |
| 1392 | Ibuprofen 800mg modified-release tablets |
| 15 | Ibuprofen 400mg tablets |
| 25794 | Isisfen 400mg Tablet (Isis Products Ltd) |
| 3053 | Naproxen 500mg gastro-resistant tablets |
| 3168 | Indometacin 25mg/5ml oral suspension sugar free |
| 33656 | Aspirin 75mg dispersible tablets (A A H Pharmaceuticals Ltd) |
| 30849 | Valdic 75 Retard tablets (Fannin UK Ltd) |
| 44639 | Aspirin 300mg Dispersible tablet (Nucare Plc) |
| 19189 | Micropirin 75mg Gastro-resistant tablet (Ratiopharm UK Ltd) |
| 2363 | Dolobid 250mg tablets (Merck Sharp & Dohme Ltd) |
| 7667 | Diclofenac 12.5mg suppositories |
| 34550 | Ibuprofen 400mg tablets film coated (Actavis UK Ltd) |
| 50652 | Junior Ibuprofen 100mg/5ml oral suspension (Numark Management Ltd) |
| 38182 | Orbifen Cold & Flu oral suspension (Orbis Consumer Products Ltd) |
| 30168 | Arthroxen 250mg Tablet (C P Pharmaceuticals Ltd) |
| 48974 | Aspirin 75mg tablets (Phoenix Healthcare Distribution Ltd) |
| 41594 | Aspirin 300mg Dispersible tablet (Teva UK Ltd) |
| 17733 | Condrotec 500mg+200microgram Tablet (Pharmacia Ltd) |
| 4565 | Fenoprofen 600mg tablets |
| 33457 | Isclofen 50mg Gastro-resistant tablet (Isis Products Ltd) |
| 32509 | Anadin Ibuprofen 200mg tablets (Pfizer Consumer Healthcare Ltd) |
| 51360 | Naproxen 250mg tablets (Accord Healthcare Ltd) |
| 42905 | Diclofenac 75mg Modified-release tablet (Actavis UK Ltd) |
| 5173 | Dexketoprofen 25mg tablets |
| 48326 | Ibuprofen 100mg/5ml oral suspension sugar free |
| 773 | Misoprostol 200microgram tablets |
| 39693 | Naproxen 200mg/5ml oral suspension |
| 30982 | Naproxen 500mg gastro-resistant tablets (Actavis UK Ltd) |
| 16 | Aspirin 75mg tablets |
| 44703 | Piroxicam 10mg Capsule (Berk Pharmaceuticals Ltd) |
| 34725 | Flurbiprofen 50mg Tablet (Bristol-Myers Squibb Pharmaceuticals Ltd) |
| 15732 | Diclovol 50mg gastro-resistant tablets (Arun Pharmaceuticals Ltd) |
| 24122 | Diclofenac sodium 50mg gastro-resistant tablets (Actavis UK Ltd) |
| 30811 | Proflex 300mg Modified-release capsule (Novartis Consumer Health UK Ltd) |
| 341 | Feldene 10mg capsules (Pfizer Ltd) |
| 24356 | Eccoxolac 300mg capsules (Meda Pharmaceuticals Ltd) |
| 37002 | Nurofen Express 200mg liquid capsules (Reckitt Benckiser Healthcare (UK) Ltd) |
| 1544 | Piroxicam 0.5% gel |
| 27571 | ORUVAIL S/R |
| 46844 | Dicloflex 75mg SR tablets (Actavis UK Ltd) |
| 25750 | Rheuflex 250mg Tablet (Goldshield Pharmaceuticals Ltd) |
| 2671 | Indometacin 50mg modified-release tablets |
| 35893 | Dicloflex Retard 100mg tablets (Almus Pharmaceuticals Ltd) |
| 24020 | Valrox 250mg Tablet (Shire Pharmaceuticals Ltd) |
| 34931 | Ibuprofen 200mg Tablet (Regent Laboratories Ltd) |
| 3935 | Feldene 20 capsules (Pfizer Ltd) |
| 25701 | Ketovail 200mg modified-release capsules (Teva UK Ltd) |
| 10917 | Flamrase SR 100mg tablets (Teva UK Ltd) |
| 32234 | Mefenamic acid 500mg tablets (IVAX Pharmaceuticals UK Ltd) |
| 34190 | Indometacin 75mg modified-release capsules (A A H Pharmaceuticals Ltd) |
| 35711 | Dicloflex 25mg gastro-resistant tablets (Teva UK Ltd) |
| 36597 | Hedex Ibuprofen 200mg tablets (Omega Pharma Ltd) |
| 5266 | Lodine sr 600mg Modified-release tablet (Shire Pharmaceuticals Ltd) |
| 18812 | Nurofen meltlets lemon 200mg Orodispersible tablet (Reckitt Benckiser Healthcare (UK) Ltd) |
| 17828 | DISPRIN CV 100 MG TAB |
| 28764 | Closteril 100mg Modified-release tablet (Pharmalife Healthcare Services Ltd) |
| 9222 | Dicloflex 75mg SR tablets (Dexcel-Pharma Ltd) |
| 53604 | Ibuprofen 200mg capsules (Numark Management Ltd) |
| 12000 | Ketoprofen 100mg suppositories |
| 31962 | Ketpron XL 200mg capsules (Mercury Pharma Group Ltd) |
| 3974 | Tenoxicam 20mg tablets |
| 53626 | Naproxen 500mg gastro-resistant tablets (Alliance Healthcare (Distribution) Ltd) |
| 50785 | Diclofenac sodium 50mg gastro-resistant tablets (Genesis Pharmaceuticals Ltd) |
| 34199 | Indometacin 100mg suppositories (Actavis UK Ltd) |
| 21610 | Rhumalgan CR 100 tablets (Sandoz Ltd) |
| 27677 | Diclofenac 75mg/3ml Injection (Antigen Pharmaceuticals) |
| 38332 | Ibucalm 200mg tablets (Aspar Pharmaceuticals Ltd) |
| 40401 | Naproxen 250mg gastro-resistant tablets (IVAX Pharmaceuticals UK Ltd) |
| 10336 | Ketoprofen 100mg/2ml solution for injection ampoules |
| 5648 | Ibuprofen 200mg orodispersible tablets sugar free |
| 2986 | Co-codaprin 8mg/400mg dispersible tablets |
| 1468 | Ibuprofen 200mg Soluble tablet |
| 37731 | Nurofen Express 342mg caplets (Reckitt Benckiser Healthcare (UK) Ltd) |
| 25257 | Advil 200mg tablets (Wyeth Consumer Healthcare) |
| 38944 | Froben SR 200mg capsules (Abbott Laboratories Ltd) |
| 29181 | Dicloflex 75mg SR tablets (Almus Pharmaceuticals Ltd) |
| 41521 | Indometacin 25mg Capsule (Approved Prescription Services Ltd) |
| 11977 | Aspro clear maximum strength tablets |
| 57585 | Asasantin Retard capsules (Dowelhurst Ltd) |
| 5482 | Sulindac 200mg tablets |
| 32242 | Ibuprofen 400mg tablets (Sterwin Medicines) |
| 10295 | Relifex 500mg/5ml oral suspension (Meda Pharmaceuticals Ltd) |
| 34980 | Ibuprofen 200mg tablets sugar coated (Actavis UK Ltd) |
| 20105 | Dicloflex 25mg Gastro-resistant tablet (Ratiopharm UK Ltd) |
| 4713 | Voltarol 75mg/3ml solution for injection ampoules (Novartis Pharmaceuticals UK Ltd) |
| 628 | Diclofenac potassium 25mg tablets |
| 24531 | Mobiflex 20mg Effervescent tablet (Roche Products Ltd) |
| 39109 | Feldene Melt 20mg tablets (Pfizer Ltd) |
| 32100 | Ibuprofen 600mg tablets (A A H Pharmaceuticals Ltd) |
| 7432 | Oruvail IM 100mg/2ml solution for injection ampoules (Sanofi) |
| 54021 | Voltarol Retard 100mg tablets (Sigma Pharmaceuticals Plc) |
| 43709 | Aspirin 75mg gastro-resistant tablets (Almus Pharmaceuticals Ltd) |
| 34271 | Diclofenac sodium 100mg modified-release tablets (A A H Pharmaceuticals Ltd) |
| 16473 | Relifex 500mg dispersible tablets (Meda Pharmaceuticals Ltd) |
| 1139 | Voltarol 25mg Tablet (Novartis Pharmaceuticals UK Ltd) |
| 9736 | Mefenamic acid 50mg/5ml oral suspension |
| 2858 | Feldene 0.5% gel (Pfizer Ltd) |
| 52420 | Celebrex 100mg capsules (Mawdsley-Brooks & Company Ltd) |
| 24320 | Indolar 50mg Capsule (Lagap) |
| 46904 | Nuromol 200mg/500mg tablets (Reckitt Benckiser Healthcare (UK) Ltd) |
| 27013 | Tiloket 200mg Modified-release capsule (Tillomed Laboratories Ltd) |
| 19398 | Radian B Ibuprofen 5% gel (Thornton & Ross Ltd) |
| 17920 | Disprin cv 100mg Modified-release tablet (Reckitt Benckiser Healthcare (UK) Ltd) |
| 2693 | Proflex 5% cream (Novartis Consumer Health UK Ltd) |
| 31054 | Phorpain Maximum Strength 10% gel (Mercury Pharma Group Ltd) |
| 6464 | Arcoxia 60mg tablets (Merck Sharp & Dohme Ltd) |
| 54075 | Voltarol 50mg dispersible tablets (Stephar (U.K.) Ltd) |
| 50628 | Ibuprofen 400mg caplets (The Boots Company Plc) |
| 6007 | Nu-Seals 300 gastro-resistant tablets (Alliance Pharmaceuticals Ltd) |
| 54565 | Aspirin 75mg dispersible tablets (Lloyds Pharmacy Ltd) |
| 34889 | Ibuprofen 400mg Tablet (Celltech Pharma Europe Ltd) |
| 2607 | Paynocil Tablet (Beecham Research Laboratories) |
| 16225 | Dexomon retard 100mg Modified-release tablet (Hillcross Pharmaceuticals Ltd) |
| 9421 | Powergel 2.5% gel (A. Menarini Farmaceutica Internazionale SRL) |
| 28255 | Naproxen 250mg tablets (Wockhardt UK Ltd) |
| 120 | Indocid 25mg capsules (Merck Sharp & Dohme Ltd) |
| 23323 | Prosaid 500mg Tablet (BHR Pharmaceuticals Ltd) |
| 53164 | Diclofenac sodium 25mg gastro-resistant tablets (Kent Pharmaceuticals Ltd) |
| 40394 | Advil 400mg Tablet (Wyeth Consumer Healthcare) |
| 41701 | Ibuprofen 600mg tablets (Actavis UK Ltd) |
| 55313 | Ibuprofen 400mg tablets (Boston Healthcare Ltd) |
| 7261 | Cuprofen 5% gel (SSL International Plc) |
| 51242 | Naproxen 500mg tablets (Pfizer Ltd) |
| 6435 | Pennsaid 16mg/ml cutaneous solution (Movianto UK Ltd) |
| 36521 | Aspirin 500mg modified-release tablets |
| 11326 | Meprobamate with ethoheptazine citrate and aspirin Tablet |
| 26095 | Ibuprofen lysine 400mg tablets |
| 417 | Diclofenac 50mg dispersible tablets sugar free |
| 25800 | Feverfen 100mg/5ml oral suspension (Wise Pharmaceuticals Ltd) |
| 42003 | Indometacin sr 75mg Capsule (Lagap) |
| 29232 | Care ibuprofen 5% Gel (Thornton & Ross Ltd) |
| 32536 | Diclofenac 25mg Tablet (Berk Pharmaceuticals Ltd) |
| 49220 | Aspirin 300mg tablets (Kent Pharmaceuticals Ltd) |
| 30382 | Ibuprofen 200mg Tablet (C P Pharmaceuticals Ltd) |
| 21816 | Pranoxen continus 500mg Tablet (Napp Pharmaceuticals Ltd) |
| 33320 | Aspirin 75mg Dispersible tablet (Sovereign Medical Ltd) |
| 34666 | Aspirin ec 300mg Gastro-resistant tablet (A A H Pharmaceuticals Ltd) |
| 11980 | Cuprofen 400mg Tablet (SSL International Plc) |
| 56007 | Aspirin 300mg dispersible tablets (Sigma Pharmaceuticals Plc) |
| 43060 | Aspirin 300mg Soluble tablet (Celltech Pharma Europe Ltd) |
| 21840 | Arthrosin 250 tablets (Ashbourne Pharmaceuticals Ltd) |
| 4880 | Diclofenac sodium 75mg gastro-resistant / Misoprostol 200microgram tablets |
| 25329 | Lofensaid 25mg gastro-resistant tablets (Opus Pharmaceuticals Ltd) |
| 34425 | Ibuprofen 400mg Tablet (Family Health) |
| 381 | Anadin Tablet (Wyeth Consumer Healthcare) |
| 57523 | Feldene 0.5% gel (Dowelhurst Ltd) |
| 4368 | Lodine 200mg Capsule (Shire Pharmaceuticals Ltd) |
| 37763 | Diclofenac 75mg/2ml solution for injection vials |
| 21382 | Aspirin 150mg / Isosorbide mononitrate 60mg modified-release tablets |
| 647 | Ibuprofen 100mg/5ml oral suspension |
| 41513 | Ibuprofen 200mg tablets (IVAX Pharmaceuticals UK Ltd) |
| 17572 | Tenoxicam 20mg powder and solvent for solution for injection vials |
| 24622 | Aspirin 325mg / Caffeine 22mg tablets |
| 140 | Naproxen 500mg suppositories |
| 25335 | PostMI 75 EC tablets (Ashbourne Pharmaceuticals Ltd) |
| 34218 | Diclofenac 25mg Gastro-resistant tablet (Pharmacia Ltd) |
| 129 | Naprosyn 500mg suppositories (Roche Products Ltd) |
| 27366 | Naproxen 500mg gastro-resistant tablets (Teva UK Ltd) |
| 589 | Voltarol 50mg dispersible tablets (Novartis Pharmaceuticals UK Ltd) |
| 1692 | Diclofenac sodium 50mg gastro-resistant / Misoprostol 200microgram tablets |
| 2235 | Relifex 500mg tablets (Meda Pharmaceuticals Ltd) |
| 3336 | Toradol 10mg tablets (Roche Products Ltd) |
| 52714 | Etodolac 600mg modified-release tablets (Alliance Healthcare (Distribution) Ltd) |
| 18234 | Rheumacin LA 75mg capsules (Hillcross Pharmaceuticals Ltd) |
| 15836 | Ocufen 0.03% eye drops 0.4ml unit dose (Allergan Ltd) |
| 56996 | Aspirin 75mg dispersible tablets (Waymade Healthcare Plc) |
| 37094 | Cuprofen 200mg tablets (SSL International Plc) |
| 26234 | Flamatrol 10mg Capsule (Berk Pharmaceuticals Ltd) |
| 4631 | Voltarol 50mg gastro-resistant tablets (Novartis Pharmaceuticals UK Ltd) |
| 39722 | Voltarol Pain-eze 12.5mg tablets (Novartis Consumer Health UK Ltd) |
| 20840 | Acetylsalicylic acid mix |
| 37750 | Piroxicam 20mg capsules (Generics (UK) Ltd) |
| 53803 | Ibuprofen 200mg capsules (Kent Pharmaceuticals Ltd) |
| 4625 | Voltarol 75mg SR tablets (Novartis Pharmaceuticals UK Ltd) |
| 21846 | Pirozip 20 capsules (Ashbourne Pharmaceuticals Ltd) |
| 2382 | Tiaprofenic acid 300mg modified-release capsules |
| 21831 | Dysman 250mg Capsule (Ashbourne Pharmaceuticals Ltd) |
| 56995 | Aspirin 75mg dispersible tablets (Phoenix Healthcare Distribution Ltd) |
| 21814 | ORUVAIL S/R |
| 26205 | Fenbuzip 300mg Capsule (Ashbourne Pharmaceuticals Ltd) |
| 30790 | Dicloflex sr 75mg Tablet (Genus Pharmaceuticals Ltd) |
| 17124 | Dicloflex sr 100mg Tablet (IVAX Pharmaceuticals UK Ltd) |
| 11461 | Ibuprofen 300mg modified-release / Codeine 20mg tablets |
| 9439 | Flurbiprofen 200mg modified-release capsules |
| 16176 | Lederfen 450mg tablets (Mercury Pharma Group Ltd) |
| 24682 | Tenoxicam 20mg effervescent tablets |
| 30389 | Contraflam 250mg Capsule (Berk Pharmaceuticals Ltd) |
| 9301 | Aspirin 100mg modified-release tablets |
| 6006 | Nu-Seals 75 gastro-resistant tablets (Alliance Pharmaceuticals Ltd) |
| 14085 | Diclovol Retard 100mg tablets (Arun Pharmaceuticals Ltd) |
| 2863 | Tiaprofenic acid 300mg tablets |
| 484 | Equagesic Tablet (Wyeth Pharmaceuticals) |
| 31938 | Aspirin 75mg gastro-resistant tablets (Sandoz Ltd) |
| 8145 | Fenbufen 300mg tablets |
| 4309 | Ibuprofen lysine 200mg tablets |
| 33662 | Aspirin 300mg Dispersible tablet (A A H Pharmaceuticals Ltd) |
| 39758 | Nurofen Express 256mg caplets (Reckitt Benckiser Healthcare (UK) Ltd) |
| 44986 | Vimovo 500mg/20mg modified-release tablets (AstraZeneca UK Ltd) |
| 24305 | Ibufac 400mg Tablet (DDSA Pharmaceuticals Ltd) |
| 47992 | Aspirin 75mg gastro-resistant tablets (A A H Pharmaceuticals Ltd) |
| 24887 | Nurofen Advance 200mg tablets (Crookes Healthcare Ltd) |
| 29772 | Ketotard XL 200mg capsules (Galen Ltd) |
| 56883 | Aspirin 75mg tablets (Waymade Healthcare Plc) |
| 21387 | Diclofenac sodium 50mg gastro-resistant tablets (Generics (UK) Ltd) |
| 827 | Voltarol 1% Emulgel (Novartis Consumer Health UK Ltd) |
| 51923 | Ibuprofen 10% gel (A A H Pharmaceuticals Ltd) |
| 7524 | Feldene 20mg dispersible tablets (Pfizer Ltd) |
| 7434 | Clinoril 100mg tablets (Merck Sharp & Dohme Ltd) |
| 34793 | Mefenamic acid 250mg capsules (Zentiva) |
| 34922 | Naproxen 500mg Tablet (Berk Pharmaceuticals Ltd) |
| 447 | Diclofenac sodium 75mg modified-release capsules |
| 8510 | Ibuprofen 5% spray |
| 29848 | Aspirin 300mg with Glycine 150mg chewable tablets |
| 41524 | Mefenamic acid 500mg tablets (Teva UK Ltd) |
| 6853 | Ibutop Ralgex Ibuprofen 5% gel (SSL International Plc) |
| 56584 | Arcoxia 60mg tablets (Lexon (UK) Ltd) |
| 29749 | Ibuprofen 200mg tablets (Ranbaxy (UK) Ltd) |
| 32365 | Relcofen 400mg tablets (Actavis UK Ltd) |
| 47994 | Naproxen 250mg Gastro-resistant tablet (Almus Pharmaceuticals Ltd) |
| 6498 | Arcoxia 90mg tablets (Merck Sharp & Dohme Ltd) |
| 53397 | Brufen 100mg/5ml syrup (Mawdsley-Brooks & Company Ltd) |
| 22206 | Nurofen Long Lasting 300mg capsules (Crookes Healthcare Ltd) |
| 56071 | Voltarol Active 4% spray (Novartis Consumer Health UK Ltd) |
| 56558 | Voltarol 12 Hour 2% Emulgel P (Novartis Consumer Health UK Ltd) |
| 1030 | Junifen 100mg/5ml Oral suspension (Crookes Healthcare Ltd) |
| 1270 | Ibuleve 5% gel (Dendron Ltd) |
| 46921 | Ibuprofen 400mg tablets (Ranbaxy (UK) Ltd) |
| 7426 | Lederfen 300mg Capsule (Wyeth Pharmaceuticals) |
| 52280 | Aspirin 300mg Tablet (Wockhardt UK Ltd) |
| 32108 | Diclofenac sodium 25mg gastro-resistant tablets (Teva UK Ltd) |
| 17525 | Fenactol Retard 100mg tablets (Discovery Pharmaceuticals Ltd) |
| 34797 | Aspirin 75mg gastro-resistant tablets (Actavis UK Ltd) |
| 56275 | Meloxicam 7.5mg tablets (Teva UK Ltd) |
| 3431 | Naproxen 250mg gastro-resistant tablets |
| 3899 | Dolobid 500mg tablets (Merck Sharp & Dohme Ltd) |
| 57162 | Diclofenac 50mg dispersible tablets sugar free (Doncaster Pharmaceuticals Ltd) |
| 38881 | Diclomax SR 75mg capsules (Galen Ltd) |
| 50926 | Aspirin 75mg dispersible tablets (The Boots Company Plc) |
| 361 | DISPRIN TAB |
| 24308 | Slo-Indo 75mg capsules (Generics (UK) Ltd) |
| 15501 | Flurbiprofen 100mg suppositories |
| 27490 | Feldene IM 20mg/1ml solution for injection ampoules (Pfizer Ltd) |
| 55099 | Acoflam 100mg Retard tablets (Mercury Pharma Group Ltd) |
| 8186 | Aspirin 300mg modified-release tablets |
| 20621 | Dicloflex 75mg SR tablets (Kent Pharmaceuticals Ltd) |
| 34738 | Naproxen 250mg gastro-resistant tablets (A A H Pharmaceuticals Ltd) |
| 20967 | Phorpain 5% gel (Mercury Pharma Group Ltd) |
| 50314 | Brufen 600mg effervescent granules sachets (Doncaster Pharmaceuticals Ltd) |
| 20978 | Anadin Ultra liquid capsules (Wyeth Consumer Healthcare) |
| 4806 | Voltarol 100mg suppositories (Novartis Pharmaceuticals UK Ltd) |
| 6226 | Aspirin 500mg / Papaveretum 7.71mg dispersible tablets sugar free |
| 917 | Diclofenac sodium 50mg tablets |
| 34977 | Naproxen 500mg Gastro-resistant tablet (Galen Ltd) |
| 14672 | Defanac 75mg SR tablets (Ranbaxy (UK) Ltd) |
| 28256 | Diclofenac 50mg Tablet (Berk Pharmaceuticals Ltd) |
| 53980 | Naproxen 250mg tablets (Phoenix Healthcare Distribution Ltd) |
| 45256 | Indometacin 25mg Capsule (Meridian Healthcare (UK) Ltd) |
| 3416 | Diclofenac sodium 100mg modified-release tablets |
| 40086 | Acoflam 50mg gastro-resistant tablets (Mercury Pharma Group Ltd) |
| 25211 | Anadin Original tablets (Pfizer Consumer Healthcare Ltd) |
| 39085 | Naproxen 250mg tablets (A A H Pharmaceuticals Ltd) |
| 156 | Diclofenac 1% gel |
| 22232 | Disprin Direct 300mg orodispersible tablets (Reckitt Benckiser Healthcare (UK) Ltd) |
| 29316 | Care ibuprofen 400mg Tablet (Thornton & Ross Ltd) |
| 43904 | Feminax Express 342mg tablets (Bayer Plc) |
| 43045 | Diclofenac potassium 50mg tablets (Actavis UK Ltd) |
| 416 | Ibuprofen 200mg tablets |
| 16474 | Nabumetone 500mg dispersible tablets sugar free |
| 45842 | Ibuprofen 600mg Tablet (Celltech Pharma Europe Ltd) |
| 21050 | Ketonal 100mg Capsule (Lagap) |
| 37587 | Etoricoxib 30mg tablets |
| 34757 | Ibuprofen 400mg Tablet (Unichem) |
| 20395 | Flamatak MR 75mg tablets (Actavis UK Ltd) |
| 34729 | Ibuprofen 400mg tablets (OBG Pharmaceuticals Ltd) |
| 14884 | Voltarol Gel Patch 1% medicated plasters (Novartis Consumer Health UK Ltd) |
| 39264 | Dicloflex Retard 100mg tablets (Dexcel-Pharma Ltd) |
| 13807 | Deep relief ibuprofen 5% Gel (Mentholatum Company) |
| 31870 | Aspirin 320mg tablets |
| 56736 | Aspirin 300mg tablets (Waymade Healthcare Plc) |
| 3492 | Diflunisal 500mg tablets |
| 28522 | Ibuprofen 200mg / Pseudoephedrine hydrochloride 30mg tablets |
| 15023 | Naproxen 375mg Modified-release tablet |
| 27968 | Apsifen 400mg Tablet (Approved Prescription Services Ltd) |
| 34438 | Mefenamic acid 250mg capsules (A A H Pharmaceuticals Ltd) |
| 34670 | Naproxen 250mg Gastro-resistant tablet (Galen Ltd) |
| 37553 | Ibucalm 400mg tablets (Aspar Pharmaceuticals Ltd) |
| 26631 | Rhumalgan XL 100mg capsules (Sandoz Ltd) |
| 112 | Ibuprofen 5% cream |
| 58071 | Voltarol Rapid 50mg tablets (Waymade Healthcare Plc) |
| 22776 | Aspirin 500mg with Cyclizine 25mg effervescent tablets |
| 31211 | Aspirin 75mg Dispersible tablet (A A H Pharmaceuticals Ltd) |
| 28888 | Galprofen Long Lasting 200mg capsules (Galpharm International Ltd) |
| 31482 | Apsifen 200mg Tablet (Approved Prescription Services Ltd) |
| 20650 | Aspirin 300mg / Paracetamol 200mg dispersible tablets sugar free |
| 28695 | Piroflam 10mg Capsule (Opus Pharmaceuticals Ltd) |
| 3077 | Oruvail 2.5% gel (Sanofi) |
| 20036 | Clotam 200mg Capsule (Thames Laboratories Ltd) |
| 41622 | Piroxicam 10mg capsules (A A H Pharmaceuticals Ltd) |
| 5896 | Ibuleve Maximum Strength 10% gel (Dendron Ltd) |
| 21123 | Piroxicam 20mg Capsule (Berk Pharmaceuticals Ltd) |
| 25361 | Diclovol 25mg gastro-resistant tablets (Arun Pharmaceuticals Ltd) |
| 41766 | Maximum Strength Aspro Clear 500mg effervescent tablets (Bayer Plc) |
| 46942 | Ibuprofen 600mg tablets (IVAX Pharmaceuticals UK Ltd) |
| 27901 | VOLTAROL RETARD |
| 2129 | Brufen retard tabs 800mg Modified-release tablet (Abbott Laboratories Ltd) |
| 13893 | Nurofen Plus tablets (Reckitt Benckiser Healthcare (UK) Ltd) |
| 46141 | Nurofen Tension Headache 342mg caplets (Reckitt Benckiser Healthcare (UK) Ltd) |
| 48138 | Ibuprofen 200mg tablets (Aspar Pharmaceuticals Ltd) |
| 392 | Ibuprofen 200mg modified-release capsules |
| 17165 | Nycopren 500mg gastro-resistant tablets (Ardern Healthcare Ltd) |
| 15159 | Tolfenamic acid 200mg Capsule |
| 2622 | Ibuprofen 800mg tablets |
| 3309 | Aspirin 325mg / Caffeine 15mg tablets |
| 35967 | Paramed Extra Power Pain Control tablets (Galpharm International Ltd) |
| 52389 | Voltarol 50mg suppositories (Sigma Pharmaceuticals Plc) |
| 23488 | Claradin 300mg Tablet (Nicholas Laboratories Ltd) |
| 34923 | Naproxen 250mg Tablet (Berk Pharmaceuticals Ltd) |
| 49862 | Voltarol 1% Emulgel (Lexon (UK) Ltd) |
| 32641 | Indometacin 25mg capsules (A A H Pharmaceuticals Ltd) |
| 407 | Brufen 600mg effervescent granules sachets (Abbott Laboratories Ltd) |
| 11907 | Dexibuprofen 400mg tablets |
| 3901 | Naprosyn EC 500mg tablets (Roche Products Ltd) |
| 28479 | Nurofen Back Pain SR 300mg capsules (Reckitt Benckiser Healthcare (UK) Ltd) |
| 27082 | Ketpron XL 100mg capsules (Mercury Pharma Group Ltd) |
| 25330 | Solpaflex tablets (GlaxoSmithKline Consumer Healthcare) |
| 41677 | Mefenamic acid 250mg Capsule (IVAX Pharmaceuticals UK Ltd) |
| 395 | Aspirin mixture |
| 1571 | Ketoprofen 100mg modified-release capsules |
| 657 | Aspirin 500mg granules sachets sugar free |
| 51829 | Naproxen 250mg tablets (Kent Pharmaceuticals Ltd) |
| 18364 | Ibular 400mg Tablet (Lagap) |
| 3 | Aspirin 75mg dispersible tablets |
| 45216 | Ibuprofen 400mg Tablet (C P Pharmaceuticals Ltd) |
| 23878 | Nu-seals cardio ec 75mg Gastro-resistant tablet (Genus Pharmaceuticals Ltd) |
| 25643 | Surgam 300mg Sachets (Sanofi) |
| 23795 | Imbrilon 100mg Suppository (Berk Pharmaceuticals Ltd) |
| 1766 | Voltarol sr 75mg Modified-release tablet (Novartis Pharmaceuticals UK Ltd) |
| 21921 | Postmi ec 300mg Gastro-resistant tablet (Ashbourne Pharmaceuticals Ltd) |
| 919 | Indometacin 100mg suppositories |
| 2234 | Nabumetone 500mg tablets |
| 51474 | Aspirin 150mg suppositories (Martindale Pharmaceuticals Ltd) |
| 35890 | Nurofen 200mg caplets (Reckitt Benckiser Healthcare (UK) Ltd) |
| 14541 | Ponstan 50mg/5ml paediatric Liquid (Chemidex Pharma Ltd) |
| 32916 | Diclofenac 75mg Modified-release capsule (Sandoz Ltd) |
| 34091 | Diclofenac sodium 25mg gastro-resistant tablets (Sandoz Ltd) |
| 46940 | Ketoprofen 100mg capsules (Generics (UK) Ltd) |
| 17750 | Indomax 25mg Capsule (Ashbourne Pharmaceuticals Ltd) |
| 39317 | Naproxen 500mg tablets (Wockhardt UK Ltd) |
| 7141 | Ibugel Forte 10% gel (Dermal Laboratories Ltd) |
| 24086 | Ibuprofen 5% gel (Thornton & Ross Ltd) |
| 10711 | Tolectin 400mg Capsule (Cilag Pharmaceuticals Ltd) |
| 9637 | Keral 25mg tablets (A. Menarini Farmaceutica Internazionale SRL) |
| 27783 | Ibuprofen 400mg tablets sugar coated (Actavis UK Ltd) |
| 17704 | Platet 100mg Effervescent tablet (Roche Products Ltd) |
| 57545 | Voltarol 1% Emulgel (Dowelhurst Ltd) |
| 10325 | Dexibuprofen 300mg tablets |
| 807 | Naproxen 500mg tablets |
| 10678 | Fenopron 300 tablets (Typharm Ltd) |
| 5455 | Etodolac 600mg modified-release tablets |
| 21150 | Strefen 8.75mg lozenges (Reckitt Benckiser Healthcare (UK) Ltd) |
| 40 | Diclofenac sodium 50mg gastro-resistant tablets |
| 51284 | Arcoxia 60mg tablets (Sigma Pharmaceuticals Plc) |
| 37053 | Migrafen 200mg tablets (Chatfield Laboratories) |
| 15930 | Ibuprofen 5% / Levomenthol 3% gel |
| 296 | Ponstan Forte 500mg tablets (Chemidex Pharma Ltd) |
| 11550 | Nurofen Meltlets 200mg tablets (Reckitt Benckiser Healthcare (UK) Ltd) |
| 16221 | Diclozip 25mg gastro-resistant tablets (Ashbourne Pharmaceuticals Ltd) |
| 15104 | Naproxen 500mg Granules |
| 15201 | Volraman 50mg gastro-resistant tablets (LPC Medical (UK) Ltd) |
| 9465 | Diclotard 100 100mg Modified-release tablet (Galen Ltd) |
| 434 | Aspirin 300mg gastro-resistant tablets |
| 7516 | Aspirin 300mg effervescent tablets sugar free |
| 4298 | Nurofen 200mg Tablet (Crookes Healthcare Ltd) |
| 7535 | Nurofen 200mg Capsule (Crookes Healthcare Ltd) |
| 29524 | Ibumetin 600mg Tablet (Alfred Benzon (UK) Ltd) |
| 23121 | Arthroxen 500mg Tablet (C P Pharmaceuticals Ltd) |
| 56503 | Asasantin Retard capsules (Mawdsley-Brooks & Company Ltd) |
| 31956 | Aspirin 75mg gastro-resistant tablets (Kent Pharmaceuticals Ltd) |
| 40083 | Ibuprofen 200mg caplets (Galpharm International Ltd) |
| 7058 | Calprofen 100mg/5ml Oral suspension (McNeil Products Ltd) |
| 10033 | Etodolac 300mg capsules |
| 54304 | Naproxen 500mg tablets (Actavis UK Ltd) |
| 53816 | Aspirin 300mg dispersible tablets (Alliance Healthcare (Distribution) Ltd) |
| 19007 | Naprosyn 500mg Granules (Roche Products Ltd) |
| 15364 | Aspirin 150mg suppositories |
| 38992 | Flamrase 75mg SR tablets (Teva UK Ltd) |
| 19046 | Ibuprofen 400mg tablets (A A H Pharmaceuticals Ltd) |
| 34359 | Ibuprofen 400mg tablets (Vantage) |
| 29455 | Flexotard MR 100mg tablets (Pfizer Ltd) |
| 47937 | Aspirin 75mg dispersible tablets (Wockhardt UK Ltd) |
| 27484 | Piroxicam 20mg/1ml solution for injection ampoules |
| 46925 | Co-codaprin 8mg/400mg dispersible tablets (Actavis UK Ltd) |
| 38527 | Proflex Pain Relief 5% cream (Novartis Consumer Health UK Ltd) |
| 19975 | Parecoxib 40mg powder for injection |
| 560 | Diflunisal 250mg tablets |
| 17131 | Lederfen 300mg tablets (Mercury Pharma Group Ltd) |
| 45331 | Ibuprofen 200mg Tablet (Co-Pharma Ltd) |
| 126 | Ponstan 250mg capsules (Chemidex Pharma Ltd) |
| 1688 | Indocid 50mg capsules (Merck Sharp & Dohme Ltd) |
| 30391 | Contraflam 500mg Tablet (Berk Pharmaceuticals Ltd) |
| 39873 | Cuprofen Maximum Strength 400mg tablets (SSL International Plc) |
| 33357 | Pacifene 200mg tablets (Sussex Pharmaceutical Ltd) |
| 45145 | Ibuleve Speed Relief Max Strength 10% gel (Dendron Ltd) |
| 1469 | Meloxicam 15mg tablets |
| 54526 | Aspirin 300mg tablets (Alliance Healthcare (Distribution) Ltd) |
| 38493 | Anadin Joint Pain 200mg tablets (Pfizer Consumer Healthcare Ltd) |
| 16918 | Flurbiprofen 0.03% eye drops 0.4ml unit dose preservative free |
| 40336 | Orudis 50mg capsules (Sanofi) |
| 51561 | Aspirin 75mg gastro-resistant tablets (Zanza Laboratories Ltd) |
| 48062 | Ibuprofen 200mg Tablet (Wockhardt UK Ltd) |
| 41615 | Indometacin 50mg Capsule (Approved Prescription Services Ltd) |
| 34616 | Piroxicam 0.5% gel (A A H Pharmaceuticals Ltd) |
| 21419 | Seractil 300mg tablets (Genus Pharmaceuticals Ltd) |
| 54463 | Diclofenac 50mg Tablet (Approved Prescription Services Ltd) |
| 16222 | Diclozip 50mg gastro-resistant tablets (Ashbourne Pharmaceuticals Ltd) |
| 45643 | Aspirin 75mg Soluble tablet (Celltech Pharma Europe Ltd) |
| 46638 | Paracetamol 500mg / Ibuprofen 200mg tablets |
| 20385 | Arthrosin 500 tablets (Ashbourne Pharmaceuticals Ltd) |
| 5268 | Naproxen 500mg modified-release tablets |
| 9688 | Diclovol 75mg SR tablets (Generics (UK) Ltd) |
| 7520 | Anadin Extra soluble tablets (Pfizer Consumer Healthcare Ltd) |
| 15286 | Ketocid 200 modified-release capsules (Chiesi Ltd) |
| 26165 | Diclofenac sodium 50mg gastro-resistant tablets (A A H Pharmaceuticals Ltd) |
| 56039 | Ibuprofen 600mg tablets (Waymade Healthcare Plc) |
| 34769 | Naproxen 500mg tablets (A A H Pharmaceuticals Ltd) |
| 3958 | Diclofenac 25mg suppositories |
| 50813 | Ibuderm 5% gel (Dermal Laboratories Ltd) |
| 39461 | Solpadeine Migraine Ibuprofen & Codeine tablets (Omega Pharma Ltd) |
| 23593 | PostMI 75 dispersible tablets (Ashbourne Pharmaceuticals Ltd) |
| 20805 | Dicloflex 75mg SR tablets (Teva UK Ltd) |
| 17068 | Nurofen Maximum Strength 10% gel (Reckitt Benckiser Healthcare (UK) Ltd) |
| 754 | Mobic 7.5mg suppositories (Boehringer Ingelheim Ltd) |
| 10913 | Diclofenac 0.1% eye drops |
| 32728 | Askit oral powder sachets (Bayer Plc) |
| 8451 | Etodolac 200mg Tablet |
| 4320 | Naprosyn 125mg/5ml oral suspension (Roche Products Ltd) |
| 34796 | Aspirin 75mg Gastro-resistant tablet (Galen Ltd) |
| 40253 | Ibuprofen 600mg Tablet (Sovereign Medical Ltd) |
| 649 | Diclofenac sodium 25mg gastro-resistant tablets |

**Drug preparation algorithm**

Decisions used for glucocorticoids, disease-modifying antirheumatic drugs:

- 4: Implausible quantity: set to population average
- 9: Missing quantity: set to population average
- 15: Implausible ndd: set to population average
- 20: Missing ndd: set to population average
- 27: Clean duration: set to 6 months if > 6 months
- 34: Select stop date if multiple: If one available use it, if two available and equal use that date, if 2 available uses mean, if 3 available uses mean of closest 2 if within 30 days.
- 41: Missing stop dates: use individual mean if unavailable use population mean
- 43: Multiple prescriptions for same product on same day: use mean ndd and mean length
- 50: Overlapping prescriptions: move later to next available time
- 53: Sequential prescriptions with short gaps: change stop gap to start of next prescriptions if gap is <= 30 days.

Decisions used for NSAIDs:

- 4: Implausible quantity: set to population average
- 9: Missing quantity: set to population average
- 15: Implausible ndd: set to population average
- 20: Missing ndd: set to population average
- 27: Clean duration: set to 6 months if > 6 months
- 34: Select stop date if multiple: If one available use it, if two available and equal use that date, if 2 available uses mean, if 3 available uses mean of closest 2 if within 30 days.
- 41: Missing stop dates: use individual mean if unavailable use population mean
- 43: Multiple prescriptions for same product on same day: use mean ndd and mean length
- 49: Overlapping prescriptions: do nothing, allow to overlap
- 53: Sequential prescriptions with short gaps: change stop gap to start of next prescriptions if gap is <= 30 days.

**Supplementary table S1: Adjusted Cox proportional hazards model for Recent GC use with point estimates for confounders.**

|  |  |  | Hazard ratio (95% confidence interval) |
| --- | --- | --- | --- |
| Exposure | Recent GC use |  | 1.17 (1.10 to 1.24) |
| Confounders^$^ | Baseline age (per year) | | 1.03 (1.03 to 1.04) |
|  | Gender | | 1.05 (0.99 to 1.11) |
|  | Baseline Charlson comorbidity index | 0 | Reference |
|  |  | 1 | 0.99 (0.92 to 1.06) |
|  |  | 2 | 0.82 (0.72 to 0.93) |
|  |  | 3+ | 0.91 (0.75 to 1.10) |
|  | Baseline ever smoker | | 0.99 (0.94 to 1.05) |
|  | Methotrexate | | 1.02 (0.97 to 1.08) |
|  | Hydroxychloroquine | | 0.83 (0.76 to 0.90) |
|  | Sulfasalazine | | 1.17 (1.10 to 1.24) |
|  | Leflunomide | | 2.57 (2.31 to 2.87) |
|  | Other csDMARDs | | 1.29 (1.12 to 1.48) |
|  | NSAIDs | | 1.26 (1.20 to 1.33) |
|  | Baseline body mass index | | - 1. 1.04 to 1.06) |

^$^ The point estimates for confounders are provided for information, but should not be over interpreted as the study was designed to study glucocorticoids as an exposure. There may be other factors, not considered here, that may be important when exploring the confounder variables as exposures.

**Supplementary table S2: Unadjusted, age and gender adjusted and fully adjusted Cox proportional hazard models in cohort with linkage for IMD 2015 and HES outpatients data (N=5860).**

|  |  | Unadjusted  HR (95% CI) | Age and gender adjusted  HR (95% CI) | Fully adjusted HR (95% CI)* | Fully adjusted with additional adjustment for IMD and disease severity  HR (95% CI) |
| --- | --- | --- | --- | --- | --- |
| Recent GC use |  | 1.37 (1.22 to 1.55) | 1.19 (1.05 to 1.35) | 1.14 (1.01 to 1.29) | 1.14 (1.00 to 1.29) |
| Recent GC dose | No GC use | Reference | Reference | Reference | Reference |
|  | >0 – 4.9mg | 1.12 (0.88 to 1.43) | 0.94 (0.73 to 1.19) | 0.93 (0.73 to 1.19) | 0.93 (0.73 to 1.19) |
|  | 5mg – 7.4mg | 1.51 (1.16 to 1.98) | 1.25 (0.95 to 1.63) | 1.21 (0.92 to 1.58) | 1.20 (0.92 to 1.58) |
|  | 7.5mg – 14.9mg | 1.39 (1.18 to 1.64) | 1.23 (1.04 to 1.45) | 1.16 (0.98 to 1.38) | 1.16 (0.98 to 1.37) |
|  | 15mg and over | 1.59 (1.22 to 2.08) | 1.44 (1.10 to 1.87) | 1.35 (1.03 to 1.76) | 1.35 (1.03 to 1.77) |
| Cumulative dose | No GC use | Reference | Reference | Reference | Reference |
|  | >0-2.49g | 1.17 (1.01 to 1.35) | 1.07 (0.92 to 1.24) | 1.06 (0.91 to 1.23) | 1.05 (0.91 to 1.22) |
|  | 2.5g - 4.99g | 1.18 (0.99 to 1.39) | 1.05 (0.89 to 1.25) | 1.06 (0.89 to 1.26) | 1.06 (0.89 to 1.26) |
|  | 5g – 9.99g | 1.36 (1.12 to 1.63) | 1.17 (0.97 to 1.41) | 1.13 (0.93 to 1.36) | 1.12 (0.92 to 1.35) |
|  | 10g and over | 1.48 (1.20 to 1.83) | 1.26 (1.02 to 1.55) | 1.15 (0.93 to 1.43) | 1.15 (0.93 to 1.42) |

- * Adjusted for baseline age, gender, baseline BMI, Baseline ever smoking, Charlson comorbidity index, time-varying DMARD and NSAID use and IMD 2015 quintile.

**Supplementary table S3: Unadjusted, age and gender adjusted and fully adjusted Cox proportional hazard models where glucocorticoid exposure has a six month attribution window.**

|  |  | Unadjusted  HR (95% CI) | Age and gender adjusted  HR (95% CI) | Fully adjusted HR (95% CI)* |
| --- | --- | --- | --- | --- |
| Recent GC use |  | 1.37 (1.29 to 1.46) | 1.19 (1.12 to 1.26) | 1.12 (1.06 to 1.19) |
| Recent GC dose | No GC use | Reference | Reference | Reference |
|  | >0 – 4.9mg | 1.34 (1.20 to 1.50) | 1.13 (1.01 to 1.26) | 1.09 (0.98 to 1.23) |
|  | 5mg – 7.4mg | 1.37 (1.20 to 1.56) | 1.09 (0.96 to 1.25) | 1.05 (0.92 to 1.20) |
|  | 7.5mg – 14.9mg | 1.38 (1.27 to 1.50) | 1.22 (1.12 to 1.32) | 1.14 (1.04 to 1.24) |
|  | 15mg and over | 1.41 (1.24 to 1.60) | 1.29 (1.13 to 1.46) | 1.20 (1.06 to 1.37) |
| Cumulative dose | No GC use | Reference | Reference | Reference |
|  | >0-2.49g | 1.22 (1.11 to 1.33) | 1.08 (0.99 to 1.18) | 1.04 (0.95 to 1.14) |
|  | 2.5g - 4.99g | 1.12 (1.02 to 1.23) | 1.00 (0.91 to 1.10) | 0.96 (0.88 to 1.06) |
|  | 5g – 9.99g | 1.25 (1.15 to 1.37) | 1.12 (1.02 to 1.22) | 1.05 (0.97 to 1.15) |
|  | 10g and over | 1.33 (1.22 to 1.45) | 1.16 (1.07 to 1.27) | 1.07 (0.98 to 1.17) |

* Adjusted for baseline age, gender, baseline BMI, Baseline ever smoking, Charlson comorbidity index, time-varying DMARD and NSAID use

**Supplementary table S4: Unadjusted, age and gender adjusted and fully adjusted Cox proportional hazard models where glucocorticoid exposure has a one month attribution window**

|  |  | Unadjusted  HR (95% CI) | Age and gender adjusted  HR (95% CI) | Fully adjusted HR (95% CI)* |
| --- | --- | --- | --- | --- |
| Recent GC use |  | 1.48 (1.39 to 1.58) | 1.25 (1.17 to 1.33) | 1.19 (1.11 to 1.27) |
| Recent GC dose | No GC use | Reference | Reference | Reference |
|  | >0 – 4.9mg | 1.43 (1.27 to 1.61) | 1.19 (1.05 to 1.34) | 1.16 (1.02 to 1.31) |
|  | 5mg – 7.4mg | 1.38 (1.20 to 1.58) | 1.10 (0.95 to 1.26) | 1.05 (0.91 to 1.21) |
|  | 7.5mg – 14.9mg | 1.49 (1.36 to 1.63) | 1.28 (1.17 to 1.41) | 1.20 (1.09 to 1.32) |
|  | 15mg and over | 1.69 (1.45 to 1.97) | 1.50 (1.29 to 1.75) | 1.41 (1.20 to 1.64) |
| Cumulative dose | No GC use | Reference | Reference | Reference |
|  | >0-2.49g | 1.13 (1.05 to 1.21) | 1.04 (0.97 to 1.12) | 1.00 (0.93 to 1.07) |
|  | 2.5g - 4.99g | 1.24 (1.12 to 1.36) | 1.07 (0.97 to 1.18) | 1.02 (0.92 to 1.13) |
|  | 5g – 9.99g | 1.37 (1.24 to 1.52) | 1.17 (1.06 to 1.29) | 1.11 (1.00 to 1.23) |
|  | 10g and over | 1.41 (1.27 to 1.56) | 1.18 (1.06 to 1.31) | - 1. 0.97 to 1.20) |

* Adjusted for baseline age, gender, baseline BMI, Baseline ever smoking, Charlson comorbidity index, time-varying DMARD and NSAID use

**Supplementary table S5: Unadjusted, age and gender adjusted and fully adjusted Cox proportional hazard models for strict hypertension outcome definition (2002 cases of hypertension).**

|  |  | Unadjusted  HR (95% CI) | Age and gender adjusted  HR (95% CI) | Fully adjusted HR (95% CI)* |
| --- | --- | --- | --- | --- |
| Recent GC use |  | 1.34 (1.20 to 1.49) | 1.20 (1.08 to 1.34) | 1.13 (1.01 to 1.27) |
| Recent GC dose | No GC use | Reference | Reference | Reference |
|  | >0 – 4.9mg | 1.26 (1.03 to 1.55) | 1.11 (0.90 to 1.37) | 1.07 (0.87 to 1.32) |
|  | 5mg – 7.4mg | 1.39 (1.11 to 1.75) | 1.19 (0.95 to 1.50) | 1.12 (0.89 to 1.41) |
|  | 7.5mg – 14.9mg | 1.36 (1.17 to 1.59) | 1.24 (1.06 to 1.45) | 1.15 (0.99 to 1.35) |
|  | 15mg and over | 1.34 (1.03 to 1.74) | 1.25 (0.97 to 1.63) | 1.18 (0.91 to 1.54) |
| Cumulative dose | No GC use | Reference | Reference | Reference |
|  | >0-2.49g | 1.13 (0.98 to 1.31) | 1.08 (0.93 to 1.24) | 1.06 (0.92 to 1.23) |
|  | 2.5g - 4.99g | 1.10 (0.94 to 1.29) | 1.03 (0.88 to 1.20) | 1.00 (0.85 to 1.17) |
|  | 5g – 9.99g | 1.32 (1.13 to 1.54) | 1.21 (1.04 to 1.41) | 1.16 (0.99 to 1.36) |
|  | 10g and over | 1.33 (1.15 to 1.53) | 1.19 (1.03 to 1.38) | - 1. 0.97 to 1.29) |

- * Adjusted for baseline age, gender, baseline BMI, Baseline ever smoking, Charlson comorbidity index, time-varying DMARD and NSAID use

**Supplementary figure legend**

**Supplementary figure S1: Flowchart of cohort inclusion.** RA diagnosis criterion: either: 1) 1 or more RA Read code and at least one DMARD prescription without an alternative indication, 2) 2 or more RA Read codes with at least one strong code and no alternative diagnosis
