# Supplementary figures and images for: Glucocorticoid use is associated with an increased risk of hypertension
Source: Rheumatology (Oxford). 2020 Jun 27;60(1):132–9. doi: 10.1093/rheumatology/keaa209 (PMC7785301; doi:10.1093/rheumatology/keaa209)

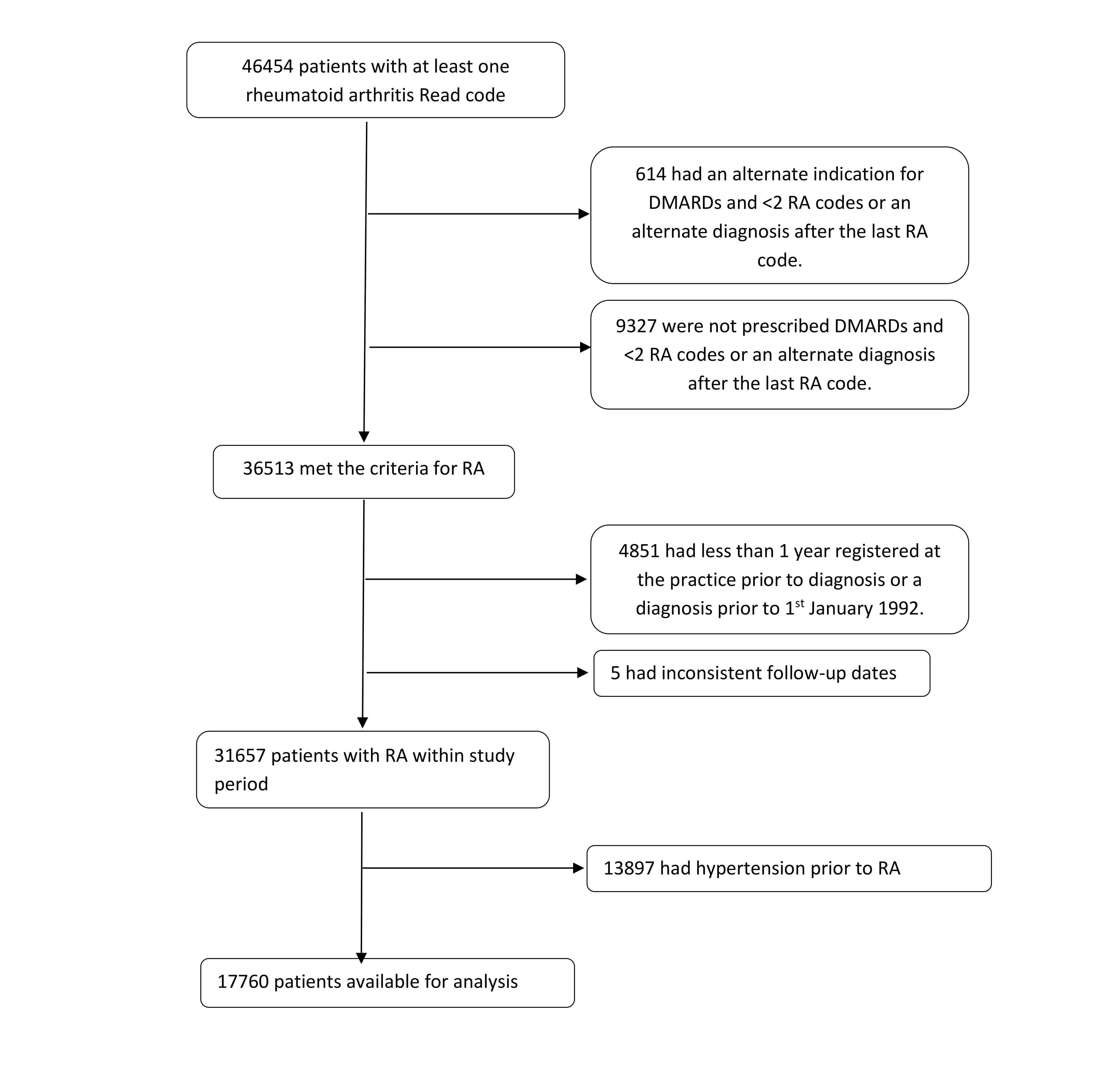

Supplement: keaa209_Supplementary_Data [file keaa209_supplementary_data.zip › keaa209-suppl_data/rhe-19-2175-File005.jpg]
